# Overweight-years and cancer risk: A prospective study of the association and comparison of predictive performance with body mass index (Atherosclerosis Risk in Communities Study)

**DOI:** 10.1002/ijc.34821

**Published:** 2023-12-24

**Authors:** Nadin K. Hawwash, Matthew Sperrin, Glen P. Martin, Corinne E. Joshu, Roberta Florido, Elizabeth A. Platz, Andrew G. Renehan

**Affiliations:** 1 Division of Cancer Sciences, School of Medical Sciences, Faculty of Biology, Medicine and Health, University of Manchester, Manchester, UK; 2 Cancer Research UK, Manchester Cancer Research Centre, Manchester, UK; 3 Centre for Health Informatics, Division of Informatics, Imaging and Data Sciences, School of Health Sciences, Faculty of Biology, Medicine and Health, University of Manchester, Manchester, UK; 4 Department of Epidemiology, Johns Hopkins Bloomberg School of Public Health, Baltimore, Maryland, USA; 5 Sidney Kimmel Comprehensive Cancer Center at Johns Hopkins, Baltimore, Maryland, USA; 6 Division of Cardiology, Johns Hopkins University, Baltimore, Maryland, USA; 7 National Institute for Health Research (NIHR) Manchester Biomedical Research Centre, Manchester, UK

**Keywords:** BMI, cancer, life-course, obesity, overweight

## Abstract

Excess body mass index (BMI) is associated with a higher risk of at least 13 cancers, but it is usually measured at a single time point. We tested whether the overweight-years metric, which incorporates exposure time to BMI ≥25 kg/m^2^, is associated with cancer risk and compared this with a single BMI measure. We used adulthood BMI readings in the Atherosclerosis Risk in Communities (ARIC) study to derive the overweight-years metric. We calculated associations between the metric and BMI and the risk of cancers using Cox proportional hazards models. Models that either included the metric or BMI were compared using Harrell’s C-statistic. We included 13,463 participants, with 3,876 first primary cancers over a mean of 19 years (SD 7) of cancer follow-up. Hazard ratios for obesity-related cancers per standard deviation overweight-years were 1.15 (95% CI: 1.05–1.25) in men and 1.14 (95% CI: 1.08–1.20) in women. The difference in the C-statistic between models that incorporated BMI, or the overweight-years metric was non-significant in men and women. Overweight-years was associated with the risk of obesity-related cancers but did not outperform a single BMI measure in association performance characteristics.

## 1 Introduction

Thirteen cancer sites are related to obesity, as stated in the 2016 International Agency for Research in Cancer (IARC) report, where sufficient strength of evidence supports a causal relationship between excess adiposity and cancer.^1^ This link is particularly concerning because of the rapidly rising prevalence of obesity worldwide.^2^ Yet, there has been little success in reducing the prevalence of obesity, such that there is a need to develop targeted and effective strategies to prevent obesity and intervene where overweight and obesity are already established.^3^ In turn, these strategies could prevent obesity-related cancers.^4^

To optimize and target obesity intervention programs, there is a need to better understand when and for how long exposure to excess adipose is most relevant to the development of obesity-related cancer. Most epidemiologic studies underpinning the evidence base that established the link between excess adiposity and cancer used a single BMI measure ascertained at a single point in time, typically in middle adulthood.

Here, we posit that a single BMI measure may not sufficiently capture the cumulative exposure and duration of exposure to excess adiposity over adulthood. As an alternative measure, the novel overweight-years metric (kg-years/m^2^) quantifies life course exposure to excess adiposity (BMI ≥25 kg/m^2^) by including both the degree of overweight in kg/m^2^ (the number of BMI units ≥25 kg/m^2^) and duration of overweight in years, similar to cigarette pack years.^5^ The obese-years metric includes both the degree and duration of obesity and similarly and may be used to quantify life course adiposity exposure due to obesity (Table S2). To date, overweight- and obese-year metrics have primarily been used in cardiovascular epidemiology^6,7^ but less so in cancer epidemiology.^8–11^

We hypothesise that overweight-years calculated across an individual’s adulthood life course improves the performance characteristics for associations with the obesity-cancer link instead of—or alongside—a single BMI measure both measured as fixed variables at the same time point at the same start of follow-up. Here, we used the Atherosclerosis Risk in Communities Study (ARIC) to (a) evaluate the association between the overweight-year metric at the start of follow-up and cancer incidence, including the components of the metric (degree and duration), and (b) compare the predictive performance of the overweight-year metric with that for BMI measured once also at the start of follow up using a linear scale. Our study is part of a larger ABACus 2 consortium project.^12^

## 2 Methods

### 2.1 Study population and data

The ARIC study recruited participants from 1987 to 1989 and followed them for cancer diagnosis until 2015. This prospective cohort study was conducted across four communities in the United States, namely Jackson, Mississippi; Washington County, Maryland; suburbs of Minneapolis, Minnesota; and Forsyth County, North Carolina and primarily investigated the causes and clinical outcomes of cardiovascular disease.^13^ Cancer outcomes were also collected, and so secondary analysis of the ARIC cohort was used to study cancer incidence. Around 16,000 participants were enrolled who were between the ages of 45 and 65. Participants attended Visit 1 and then three more visits each 3 years apart. Fifteen years after Visit 4 (1996–1998), they attended Visit 5 (2011–2013), and then more frequently Visits 6 through 8.^14^ Visit 9 has now been completed.

Participants needed at least three BMI measurements including a recall BMI reading at age 25 and a BMI reading measured at Visit 1 and Visit 2 to be included in our study. Participants were followed up until cancer diagnosis, death or administrative censoring on 31 December 2015. Participants were excluded if they (a) were over age 80 at Visit 2 given the potential survivorship bias beyond this age and reduction of BMI for the same body composition from skeletal muscle loss beyond this age,^15^ (b) prevalent cancer at Visit 2 as the population of interest was cancer-free individuals, (c) extreme BMI readings of ≤15 kg/m^2^ and >60 kg/m^2^, (d) missing cancer follow-up data or no cancer follow-up period and (e) participants who were not of Black or White race given the very small numbers in other racial groups.

### 2.2 Exposure

The period of exposure (age 25 through Visit 2) and follow-up (Visit 2 through 31 December 2015) in our study are depicted in Figure S1. Visit 2 was chosen as the study baseline given most participants would have three BMI readings by this point and a study baseline at any point beyond this may increase the risk of selection bias. Weight and height were measured by trained personnel using a standardized protocol across the four field centres at each study visit. BMI was calculated as weight in kilograms divided by the square of height in metres. Using yearly predicted BMI values, we calculated the overweight-years metrics by multiplying the prior degree of overweight (the number of BMI units ≥25 kg/m^2^) by the duration of that degree of overweight (the time in years between the prior and current observation). To include BMI readings at 25 kg/m^2^, we subtracted BMI by 24.9 to calculate the degree of overweight. An example calculation of the overweight-years metric is shown in Table S1. Total cumulative overweight-years were calculated as the sum of overweight-years from age 25 to Visit 2. Cumulative overweight degree and duration were calculated as the sum of prior overweight degrees and durations respectively (Table S1). Exposures of overweight-years and BMI per standard deviation and per 100 kg-years/m^2^ and BMI per 5 kg/m^2^ were analysed in line with prior literature.^8^ Baseline in this article refers to Visit 2; the time point by which a single BMI and overweight-years were calculated to.

### 2.3 Outcomes

Cancer diagnoses were ascertained by linkage to four state cancer registries supplemented with medical records and hospital discharge summaries.^16^ The primary outcome in our study was primary cancer incidence, which was separately studied by cancer sites (ie, obesity-related and non-obesity-related cancers). Obesity-related cancers included colorectal, gastric, oesophageal, thyroid, kidney, liver, pancreatic, multiple myeloma, gallbladder, meningioma, postmenopausal breast, endometrial and ovarian cancer. Postmenopausal breast cancer was defined by participants with a breast cancer diagnosis at age 55 or above given menopausal status was not repeatedly collected on each Visit. Cancers aside from those in the obesity-related cancer subgroup were listed as non-obesity-related cancers. Other outcomes of interest included cancer-specific sites of those with at least 10 events per candidate predictor parameter (EPP) where a candidate predictor is any covariate on the list of possible covariates to include in models.^17^ Consequently, the cancer-specific sites investigated were colorectal, kidney, lung, pancreas, prostate, and bladder cancer in men and women colorectal, postmenopausal breast cancer, endometrial, ovarian, kidney, lung and pancreatic cancer.

### 2.4 Covariates

We used self-reported covariates such as race, smoking, alcohol (g/day), and hormone replacement therapy (HRT) in women only from baseline, except education and race which were collected at Visit 1. We categorized race as ‘White’ and ‘Black’, smoking as ‘ever smokers’ and ‘never smokers’ and HRT as ‘ever HRT users’ and ‘never HRT users’. Pack-years of cigarette smoking, age of menarche and age of menopause are risk factors for some obesity-related cancers, but age of menarche and menopause were only collected at Visit 1; therefore, these covariates were assumed to remain constant till Visit 2 and were only adjusted for in a sensitivity analysis.

### 2.5 Statistical analysis

We defined the follow-up for each participant as the number of years from Visit 2 until either cancer diagnosis, administrative censoring, or death, whichever occurred first. The association between excess adiposity and cancer risk is known to vary by sex^18^; therefore, all analyses were separated by sex and only within-sex comparisons have been made.

To account for missing covariate data, missing values were imputed by multiple imputation with the following variables included in the predictor matrix ‘ever diagnosed with diabetes’, ‘ever diagnosed with cardiovascular disease’, ‘smoking’, ‘race’, ‘education’, ‘HRT (women only)’, ‘alcohol’, ‘cancer age’, ‘cancer incidence’.^18^ We imputed 10 datasets and checked the convergence of the imputation procedure. All analyses were completed on the 10 imputed datasets separately before pooling under Rubin’s rules.^19^

BMI was predicted per year from age 25 (the minimum age BMI was recalled in the ARIC study) until Visit 2 by a linear mixed effects model fitted to the observed BMI measures. This model included a random intercept, random slope and an interaction term between sex and a spline on age (with knots at ages 50, 60 and 70). Following the calculation of overweight-years exposure using predicted BMI measures, we calculated the cancer incidence rate, per 1000 person-years, across overweight-year groups of 0, >0–100, and >100 kg-years/m^2^, stratified by sex, race, smoking and HRT use (in women). Cox proportional hazards models were used to calculate the age- and multivariable-adjusted hazard ratios (HR) of cancer incidence per standard deviation (SD) of overweight-years, cumulative overweight degree, cumulative overweight duration, and BMI at Visit 2. Multivariable-adjusted models adjusted for baseline age (ie, the age at Visit 2), race, smoking, alcohol and HRT (in women). Age at baseline was adjusted for in our study given the age follow-up started varied for each participant and needed to be adjusted for. Schoenfeld residuals were used to test the Cox proportional hazards assumption. For continuous variables that violated the assumption, time-varying coefficients were included, and categorical variables were stratified.

Comparisons of the overweight-years metric and BMI (both as recorded at Visit 2) were made using Harrell’s C-statistic, identifying which metric had the higher performance characteristics of the association between excess adiposity and cancer incidence. Bootstrapping with 100 iterations was used to adjust the calculation of the C-statistic for in-sample optimism (Table S10).^20^ Differences between C-statistic values of each metric were compared. The Akaike information criterion (AIC) of each metric was also used to compare the models that included each of the exposure measures separately.

Further analysis with the obese-years metric using a threshold BMI of ≥30 kg/m^2^ was completed and included in Data S1. The analysis also included Cox proportional hazards models per 100 kg-years/m^2^, per 5-unit BMI, per 10-unit degree and 10-year duration on cancer incidence given the consistent use in the literature.^1,8^

Analysis was completed using R 4.1.2, and the following packages: magrittr,^21^ tidyverse,^22^ haven,^23^ purrr,^24^ lme4,^25^ survival,^26^ survminer,^27^ lubridate,^28^ kableExtra,^29^ gtsummary,^30^ splines,^31^ Ecdat,^32^ interactions,^33^ rms,^34^ ggplot2,^35^ ggpubr,^36^ lattice,^37^ arsenal^38^ and Hmisc.^39^ High Performance Computing was used to run models. We reported our study using the Strengthening the Reporting of Observational studies in Epidemiology (STROBE) guidelines (Table S3).^39^

### 2.6 Sensitivity analysis

A sensitivity analysis of associations per SD overweight with adjustment for smoking pack-years and height in men and smoking pack-years, height, age of menarche and age of menopause in women was completed as these are established risk factors for several obesity-related cancers. To test the robustness of our findings, the analysis described above was repeated on (a) individuals with at least three BMI readings using observed BMI readings only to establish if BMI predictions in the main analysis were reliable to use and (b) using BMI predicted from age 25 until Visit 2 of those with at least 1 BMI reading using a linear mixed-effects model with a random intercept, a random slope, a spline on age with knots at ages 50, 60, 70 and an interaction by sex. The subgroup with 1 BMI reading was modelled to identify any selection bias toward healthier individuals. Additionally, non-obesity-related cancers without prostate and lung cancers were analysed, given the potential detection bias associated with prostate cancer and lack of complete adjustment for confounding by smoking for lung cancer.^40,41^ This could result in a misleading impression of a ‘protective’ effect of obesity on non-obesity-related cancers. Additionally, in terms of lung cancer, disentangling the effect of smoking on body weight and associated cancer risk may be challenging due to the complexity of reverse causation and residual confounding.^41^

## 3 Results

**Figure 1:**
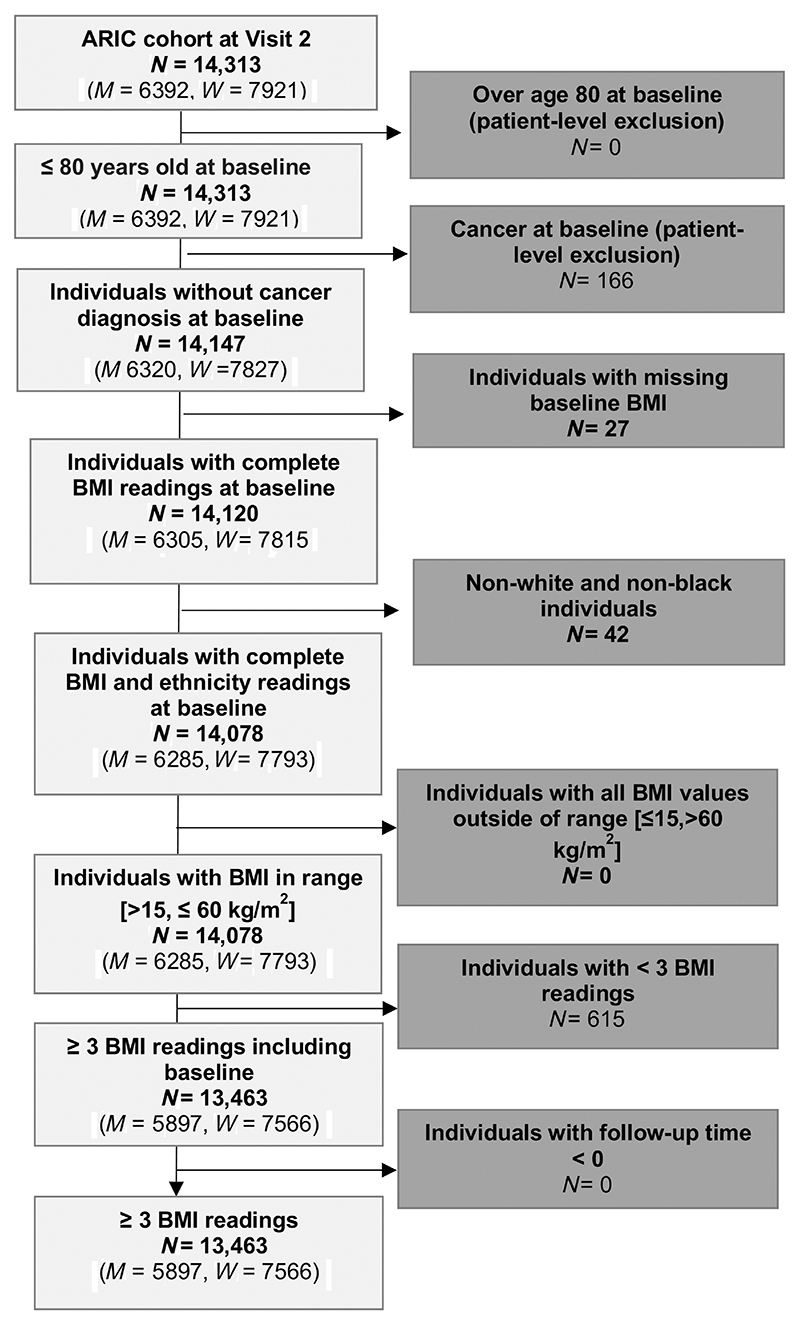
Flow diagram of individuals in the ARIC cohort. Baseline refers to Visit 2. Excluded individuals (dark grey). Included individuals (light grey). BMI-related exclusion criteria were observational level exclusions but led to individual exclusions if all BMI measurements were outside the range. ARIC, Atherosclerosis Risk in Communities (Study), BMI, body mass index; *N*, number of participants.

We included a total of 13,463 participants, comprised of 44% men and 56% women (Figure 1). Characteristics of the analytic cohort at baseline are described in Table 1. The mean age was 57 years (SD 6) for men and women with mean baseline BMIs of 27.7 kg/m^2^ (SD 4.3) and 28.2 kg/m^2^ (SD 6.1) respectively. 20% of men and 28% of women were Black. The mean overweight-years exposure was 49.93 (SD 64.75) for men and 84.47 (SD 100.46) for women. A total of 2,072 cancers were diagnosed in men and 1,804 were diagnosed in women over a mean follow-up period of 18 years (SD 8) in men and 20 years (SD 7) in women (Table S4). Analyses of the incidence rate of all cancers per 1000 person-years stratified by race, smoking and HRT in women showed that in all subgroups over 100 kg-years/m^2^ exposure had the highest cancer incidence for both men and women except for ever HRT users where >0–100 kg-years/m^2^ exposure had a higher cancer incidence than the subgroup with over 100 kg-years/m^2^ exposure as shown in Table S5.

### 3.1 Associations between overweight years and cancer

The multivariable-adjusted HR for obesity-related cancers per unit SD of overweight-years was 1.15 (95% CI: 1.05–1.25) for men and 1.14 (95% CI: 1.08–1.20) for women (Table 2). Significant positive associations per SD increment in overweight-years were also found for colorectal cancers in men with a HR 1.32 (95% CI: 1.18–1.47) and pancreatic, kidney and endometrial cancers in women with HRs of 1.33 (95% CI: 1.08–1.65), 1.29 (95% CI: 1.03–1.6) and 1.51 (95% CI: 1.29–1.76), respectively. Bladder cancer in men had a non-significant positive association per SD increment in overweight-years and BMI with HRs of 1.17 (95% CI: 0.95–1.45) and 1.18 (95% CI: 0.93–1.49), respectively. For pancreatic cancer in women, there was a significant positive association per SD overweight-years but a non-significant positive association per SD BMI with HRs of 1.33 (95% CI: 1.08–1.65) and 1.16 (95% CI: 0.89–1.52), respectively. For postmenopausal breast cancer in women, a significant positive association was found per SD increment in BMI, but the association was non-significant per SD overweight-years exposure with HRs of 1.11 (95% CI: 1.02–1.21) and 1.07 (95% CI: 0.99–1.16), respectively. Schoenfeld residuals for Cox proportional hazards models were independent of time and thus in agreement with the proportional hazard assumption after stratifying by race and using time-varying age. Further analysis with multivariable-adjustment including smoking pack-years and height in men and women and also age of menopause and age of menarche in women showed little variation in HRs per SD overweight-years (Table S6). Analysis per 100 overweight-years and per 5-unit BMI is shown in Table S7 and analysis per 10 kg/m^2^ degree of obesity and per 10-year duration of obesity is shown in Table S8. Analysis by AIC demonstrated little variance in AIC across metrics (Table S9).

There was a non-significant positive association for obesity-related cancers per SD increment in cumulative degree of overweight and a non-significant inverse association per SD cumulative duration of overweight in men with HRs of 1.10 (95% CI: 0.97–1.25) and 0.97 (95% CI: 0.85–1.09), respectively (Table 3). For all cancers in men, there was no evidence of a significant association per SD degree and duration except for colorectal cancer there was a positive association per SD cumulative degree of overweight [HR 1.29 (95% CI: 1.10–1.51)] but the association per SD cumulative duration of overweight was non-significant [HR 1.11 (95% CI: 0.92–1.33)]. For bladder cancer in men, there was a positive but non-significant HR per SD cumulative degree and duration of overweight of 1.12 (95% CI: 0.82–1.52) and 1.04 (95% CI: 0.77–1.40), respectively. Inverse associations were found per SD cumulative degree and duration of overweight for kidney and pancreatic cancer in men; however, for lung cancer, there was a positive non-significant association per SD cumulative degree of overweight and a non-significant inverse association per SD cumulative duration of overweight with HRs of 1.10 (95% CI: 0.95–1.28) and 0.98 (95% CI: 0.86–1.13), respectively (Table 3). For prostate cancer, there was no evidence of an association per SD cumulative degree of overweight but a non-significant positive association per SD cumulative duration of overweight with HRs of 1.00 (95% CI: 0.9–1.10) and 1.05 (95% CI: 0.96–1.14), respectively. For metastatic prostate cancer, associations were both positive and non-significant per SD cumulative degree and duration of overweight with HRs of 1.20 (95% CI: 0.87–1.67) and 1.20 (95% CI: 0.86–1.69), respectively.

In women, there was a non-significant positive association for obesity-related cancers combined per SD degree and duration of overweight, with HRs of 1.03 (95% CI: 0.94–1.12) and 1.03 (95% CI: 0.96–1.12), respectively (Table 3). For colorectal cancer in women, there was a non-significant inverse association per SD cumulative degree of overweight and a non-significant positive association per SD cumulative duration of overweight with HRs of 0.90 (95% CI: 0.72–1.13) and 1.03 (95% CI: 0.85–1.24), respectively. For pancreatic cancer in women, there was a significant positive association per SD cumulative degree of overweight and a non-significant positive association per SD cumulative duration of overweight with HRs of 1.49 (95% CI: 1.10–2.02) and 1.26 (95% CI: 0.89–1.79), respectively. For endometrial and postmenopausal breast cancer, there was no evidence of an association per SD cumulative degree of overweight but a positive non-significant associations per SD cumulative duration of overweight with HRs 1.04 (95% CI: 0.79–1.37) and 1.02 (95% CI: 0.91–1.13), respectively (Table 3).

### 3.2 Model performance characteristics

In men and women, overall, there were marginal differences between the C-statistic across the metrics (overweight-years, BMI, cumulative degree and duration of excess overweight indicating similar performance characteristics) (Tables 4 and S11). However, in men, for pancreatic cancer, the combined metric of overweight-years with BMI measured at a single time had a significantly higher C-statistic of 0.582 (95% CI: 0.536–0.632) than overweight-years or BMI alone with C-statistics of 0.542 (95% CI: 0.499–0.589) and 0.551 (95% CI: 0.506–0.599), respectively. In women, for obesity-related cancers, the C-statistic was significantly higher by a difference of 0.012 (95% CI: 0.001–0.024) for the combined overweight-years and BMI metric (C-statistic 0.573, 95% CI: 0.557–0.590) than the overweight-years metric separately (C-statistic 0.562, 95% CI: 0.551–0.573) (Table 4).

### 3.3 Associations between obese-years and cancer risk

To identify if the obese-years metric was more informative of the link between cumulative excess adiposity and cancer incidence than single BMI, the analysis was repeated using obese-years (Tables S12–S18). Multivariable-adjusted HRs per SD obese-years 1.15 (95% CI: 1.05–1.25) in men and women 1.11 (95% CI: 1.05–1.17) (Table S13). In men, a significant positive association was found per SD increment in obese-years for non-obesity-related cancers of 1.06 (95% CI: 1.01–1.12) but not per SD BMI [HR 1.01 (95% CI: 0.95–1.07)]. For non-obesity-related cancers excluding lung and prostate in men, significant positive associations were found per SD obese-years and BMI of 1.13 (95% CI: 1.04–1.22) and 1.12 (95% CI: 1.02–1.23), respectively. The only significant positive associations found per SD increment in obese-years were for colorectal cancer in men [HR 1.23 (95% CI: 1.11–1.37)] and pancreatic, kidney and endometrial cancer in women with HRs of 1.22 (95% CI: 1.03–1.46), 1.24 (95% CI: 1.05–1.48) and 1.36 (95% CI: 1.20–1.54), respectively (Table S13).

Analysis per SD increment in cumulative degree and duration of obesity in men showed significant positive associations for obesity-related cancers per SD degree of obesity but a non-significant association per SD duration of obesity with HRs of 1.11 (95% CI: 1.01–1.22) and 1.11 (95% CI: 0.99–1.23), respectively (Table S15). For lung cancer, men had a significant positive association per SD cumulative duration of obesity but a non-significant positive association per SD cumulative degree of obesity with HRs of 1.15 (95% CI: 1.01–1.29) and 1.10 (95% CI: 0.95–1.26), respectively. In women, for obesity-related cancers there was no evidence of an association per SD cumulative degree of obesity but a positive non-significant association per SD cumulative duration of obesity of 1.01 (95% CI: 0.94–1.09). In women, pancreatic cancer had a significant positive association per SD duration of obesity and a non-significant positive association per SD cumulative degree of obesity of 1.42 (95% CI: 1.08–1.89) and 1.26 (95% CI: 0.99–1.62), respectively. A non-significant inverse association was found for postmenopausal breast cancer per SD cumulative degree of obesity but not duration with HRs of 0.97 (95% CI: 0.87–1.09) and 1.03 (95% CI: 0.92–1.15), respectively (Table S15).

On analysis of the C-statistic of each metric, primarily there were no statistically significant differences in the concordances across all metrics for obesity exposure in men apart from for pancreatic cancer the combined obese-years with BMI was significantly higher than BMI as a metric alone with a difference in C-statistic of 0.032 (95% CI: 0.012–0.053). For combined obesity-related cancers in women, BMI had a significantly higher C-statistic than obese-years and a significantly higher C-statistic for combined obese-years and BMI as a metric than obese-years alone (Table S18).

### 3.4 Sensitivity analysis

Analysis of both metrics was repeated using the observed (rather than predicted) BMI measures from the subgroup with at least three BMI readings (Tables S19–S33) and the subgroup with at least 1 BMI reading (Tables S34–S48). Results in both sensitivity analyses using metrics derived from observed BMI and predicted BMI from participants with at least 1 BMI reading were largely similar to the main analysis. Comparison of concordance in both sensitivity analyses mainly showed non-significant differences in C-statistics between the measures overall (Tables S26 and S41). In men, there was a significantly higher C-statistic by 0.014 (95% CI: 0.003–0.025) for cumulative overweight duration than a cumulative overweight degree for lung cancer with a C-statistic of 0.739 (95% CI: 0.721–0.757) and 0.725 (95% CI: 0.707–0.743), respectively (Table S26). In women, non-obesity-related cancers combined both including and not including lung cancers had a significantly higher C-statistic for overweight duration than the degree of overweight. For colorectal and endometrial cancers in women, the C-statistic was significantly higher for BMI than in overweight-years (Table S26). For the subgroup with at least 1 BMI reading, bladder and lung cancer had a significantly higher C-statistic for the combined overweight-years and BMI metric than BMI alone in men and women (Table S41). For the analyses using obese-years, overall, there was no significant difference in predictive performance across all metrics in both sensitivity analyses (Tables S18 and S48). In both sensitivity analyses, endometrial cancer in women had a significantly higher C-statistic per SD cumulative degree than the duration of obesity (Tables S18 and S48).

## 4 Discussion

We found that the overweight-years metric is associated with obesity-related cancers, but not beyond its two components. Further, in most cases, the metric does not outperform BMI measured at the same time point in the association performance characteristics. However, for pancreatic cancer, we found that the combined overweight-years metric with BMI had a greater predictive performance than single BMI and overweight-years measures alone. In women, the combination of overweight-years with BMI outperformed the performance characteristics of overweight-years measured at the same time point for obesity-related cancers combined. Significant positive associations were found in men and women between overweight-years and cancer risk for obesity-related cancers combined. The strongest association for obesity-related cancers per SD overweight-years was for colorectal cancer in men and endometrial cancer in women. Variations in associations between overweight-years and cancer incidence were sex-specific and cancer site-specific which is line with a prior study by Renehan et al which used single BMI measurements.^42^ In line with our study, Renehan et al found strong associations for colon cancer in men and endometrial and kidney cancers in women.^42^ However, our study did not find a strong association for kidney cancer per SD overweight-years exposure for men unlike the strong association found by Renehan et al per 5-unit BMI. In men, for obesity-related cancers combined, there was a non-significant positive association per SD degree but a non-significant inverse association per SD duration of overweight, whereas in women there was a non-significant positive association both per standard degree and duration of overweight. Our findings support that prevention interventions should focus on minimising the degree of overweight in men and the degree and duration of overweight in women.

Exact mechanisms underlying the association between excess adiposity and cancer remain unknown and should be explored to identify causal links between the degree and duration of overweight and obesity and cancer incidence although possible explanations include chronic inflammation, insulin resistance and oxidative DNA damage.^1,43^ A possible explanation for the increased risk of endometrial cancer is the increased oestrogen levels associated with excess adiposity due to increased androstenedione production and oestrone involved in its production and the increased activity of the enzyme aromatase in peripheral adipose tissue.^42^ Contrary to the IARC 2016 report of over 1000 epidemiologic studies, which concluded inadequate evidence, we found that BMI and overweight-years had positive but non-significant HRs for bladder cancer in men and that the cumulative duration and degree of overweight contributed to bladder cancer risk.^1^ A previous study by Roswall et al which analysed 390,878 participants in the European Prospective Investigation into Cancer and Nutrition cohort found a positive association among men but not women between BMI and bladder cancer risk.^44^ Further research into analysis by race will be undertaken as part of the ABACus 2 consortium project, which may further explain this finding.^12^

Our study supports the existing literature that there is a significant association between the duration of obesity and pancreatic cancer in women.^45^ Elevated fasting blood glucose levels were associated with an increased risk of pancreatic cancer with a 14% increased risk for every 10 mg/mL increase in fasting blood glucose.^46^ Insulin resistance, insulin-like growth factor 1, elevated insulin levels and diabetes are also associated with increased cancer risk which potentially explains associations between cumulative duration of excess BMI and pancreatic cancer risk.^46,47^

To our knowledge, our study is the first to analyse overweight- and obese-years with cancer incidence stratified by men and women. Similar analyses were completed by Arnold et al using the Women’s Health Initiative (WHI) but only included women.^8^ Our finding that both degree and duration predict the obesity-cancer link in women was consistent with the WHI findings.^8^ The sensitivity analysis using at least 3 observed BMI measurements per participant was useful to validate whether results from the main analysis using predicted BMI measures from the same cohort were similar. Analysis using predicted BMI measures removed sensitivity to the ages that BMI was measured and accounted for measurement error. The sensitivity analysis with at least one BMI reading was used to analyse results whilst minimising selection bias—those who completed sufficient recalls may be systematically different to those who did not.

## 5 Strengths

A strength of our study was the use of repeated BMI data from a well-established longitudinal cohort study in the United States and the inclusion of both Black and White participants so findings may be generalisable to more populations compared to previous studies. Another strength was the inclusion of both men and women, and the stratification of the analysis by sex, given the established variability in BMI by sex.^42^ Additionally, a strength of our study was the formal comparison with a single BMI measure at a point in time in terms of predictive performance; furthermore, there was added strength in the analysis of the cumulative degree and duration metrics separately to identify and compare the related cancer risk associated with each metric as overweight- and obese-years metrics alone are composite measures and do not demonstrate whether degree or duration contribute to cancer risk. A further strength was the use of cancer registry data which has been previously shown to be more accurate than self-reported data.^48^

## 6 Limitations

One of the main limitations of our study was that there was only one single recalled weight measure in early adulthood at age 25 before Visit 1 (at which participants were in mid-adulthood between 45 and 65 years of age). BMI was not predicted before age 25 so adulthood BMI exposures in ages 18–25 were not accounted for; therefore, the cumulative degree and duration of overweight exposures may be underestimated. Additionally, excess BMI exposures in childhood were also not captured given the lack of BMI data available at those ages. Cancer registry linkage was last updated in 2015 which may have underestimated associations for late-onset cancers. A limitation of our study was the use of measured BMI data from 1987 to 1998 which may not be generalisable to the current population given the worldwide rise and earlier onset of obesity over the last four to five decades.^2^ Stratification by race did not take place in our study but race will be further analysed in the ABACus 2 project to identify effect modifiers of the obesity-cancer link.^12^ Another limitation of our study was that only one cohort was analysed and although the sample size was sufficient for the analysis provided, not all cancer types could be analysed, and we could not stratify by cancer site subgroups as there were low site-specific numbers of cancer events. It is important to note the potential of reverse causation and residual confounding by smoking as a limitation of our study in particular for lung cancer where an inverse association was found with BMI. Additionally, it is important to note the potential reverse causation for undiagnosed cancers at Visit 2 potentially causing a reduction in BMI in baseline and pre-baseline BMI measurements. Mendelian randomisation studies previously undertaken to circumvent these limitations found positive associations with BMI and lung cancer.^49^ Although exercise and red meat consumption are potential confounders of the excess BMI and cancer link, these variables were not adjusted for given they were not collected at Visit 2 in the ARIC study and are likely to change over time. Additionally, adjustment for too many variables in a model may lead to overfitting.

## 7 Unanswered Questions and Future Research

Our study outlines the importance of overweight degree and duration with proven associations with cancer incidence. Future work includes analysis of more recent prospective cohorts and the incorporation of weight-cycling through analysis of a cohort with more frequently collected data on BMI so variations in BMI can be incorporated in prediction models and potentially improve the predictive performance. Further study metrics similar to that of overweight-years but using waist circumference will be used in the ABACus 2 project and will be compared to single BMI and single WC measures.^12^ For instance, the use of waist circumference years metrics would particularly be useful to explore as for the same BMI of 25 kg/m^2^, there was an increase of waist circumference by 1.1 cm in men and by 4.9 cm in women over 30 years.^50^ Therefore, for the same BMI of an individual over adulthood, an individual may have a higher percentage of muscle mass at a younger age but a higher fat mass at an older age. Other measures that could be explored include body fatness percentage and magnetic resonance-determined fat measures.^51^ So, exploring metrics that consider the degree and duration of excess adiposity will help identify those with the best predictive performance of the association between excess adiposity and cancers. As with most observational studies, we could not fully exclude confounding effects despite adjusting for covariates. Future research could incorporate genetic instruments able to predict childhood and adulthood obesity in a study to assess the life course exposure to excess adiposity and cancer incidence.^52^

## 8 Conclusion

Adulthood obesity-related cancer changes quantified using overweight- or obese-year alongside a single BMI measure have shown to have similar performance characteristics to either measure alone. Therefore, due to its simplicity and lack of need for repeated BMI measures over adulthood, BMI should still be used as a measure of excess adiposity in cancer studies. Our findings confirm that overweight degree and duration are associated with cancer incidence, and both should be considered in prevention strategies. A future individual participant data meta-analysis across the ABACus 2 consortium will take place to confirm whether these findings are generalisable to other populations.

## Supplementary Material

Data S1. Supporting Information.

## Figures and Tables

**Table 1:** Characteristics^a^ of the analytic cohort at Visit 2, ARIC.

Characteristic	Men			Women		
	0—Overweight-years^a^, kg-years/m^2^ (*N* = 1169)	≤100—Overweight-years^a^, kg-years/m^2^ (*N* = 3068)	>100—Overweight-years^a^, kg-years/m^2^ (*N* = 1660)	0—Overweight-years^a^, kg-years/m^2^ (*N* = 1574)	≤100—Overweight-years^a^, kg-years/m^2^ (*N* = 3512)	>100—Overweight-years^a^, kg-years/m^2^ (*N* = 2480)
Age at baseline, years, mean (SD)	57(6)	57(6)	59(6)	56(6)	56(6)	58(6)
Height, m, mean (SD)	1.77 (0.07)	1.76 (0.06)	1.74 (0.06)	1.64 (0.06)	1.63 (0.06)	1.61 (0.06)
Follow-up, years, mean (SD)	18 (8)	18 (8)	16(8)	21(7)	20 (7)	19(7)
BMI at baseline, kg/m^2^, mean (SD)	23.0 (1.7)	27.4 (2.4)	31.7 (4.5)	22.4 (1.9)	27.0 (3.7)	33.0 (7)
Underweight, *n* (%)	24(2)	4(0)	0(0)	49 (3)	34(1)	9(0)
Normal weight, *n* (%)	1083 (93)	372 (12)	87 (5)	1429 (91)	892 (25)	219 (9)
Overweight, *n* (%)	62(5)	2277 (74)	503 (30)	96(6)	1858 (53)	553 (22)
Obese, *n* (%)	0(0)	415 (14)	1070 (64)	0(0)	728 (21)	1699 (69)
Total cumulative overweight degree, kg/m^2^ mean (SD)	0(0)	27 (20)	140 (68)	0(0)	43 (31)	209 (96)
Total cumulative overweight duration, years, mean (SD)	46 (4)	15 (8)	29(7)	43 (3)	18 (9)	31(6)
WC at baseline, cm, mean (SD)	89(6)	100 (7)	109 (12)	82 (8)	94 (12)	108 (17)
Missing	0	2	0	1	0	2
Ethnicity, *n* (%)						
White	921 (79)	2491 (81)	1295 (78)	1379 (88)	2535 (72)	1561 (63)
Black	248 (21)	577(19)	365 (22)	195 (12)	977 (28)	919 (37)
Smoking, *n* (%)						
Ever	890 (76)	2239 (73)	1220 (74)	879 (56)	1776 (51)	1090 (44)
Never	276 (24)	824 (27)	433 (26)	692 (44)	1733 (49)	1385 (56)
Missing	3	5	7	3	3	5
Alcohol consumption, units of alcohol per week, mean (SD)	5(10)	5(10)	4(8)	2(4)	1(3)	1(3)
Missing	5	8	7	3	3	5
HRT, *n* (%)						
Ever				608 (44)	1110 (37)	524 (26)
Never				761 (48)	1857 (53)	1491 (60)
Missing				205	545	465
Education, *n* (%)						
Graduate or professional school	161 (14)	414 (14)	187(11)	152 (10)	299 (9)	148 (6)
College	315 (27)	905 (30)	438 (26)	469 (30)	894 (25)	481 (19)
Vocational school	109 (9)	293 (10)	151(9)	136 (9)	260 (7)	200 (8)
Completed high school	307 (26)	847 (28)	454 (27)	613 (39)	1343 (38)	899 (36)
High school but no degree	137 (12)	344 (11)	221 (13)	143 (9)	500 (14)	456 (18)
Grade school or 0 years education	136 (12)	262 (9)	204 (12)	60(4)	213 (6)	291(12)
Missing	4	3	5	1	3	5

*Note*: Values in parentheses are percentages unless otherwise stated. Covariates are from the start of follow up at Visit 2 except for education and ethnicity collected at Visit 1 of the ARIC Study. Baseline refers to Visit 2. BMI is calculated from weight and height that was measured in standard way by trained technicians at each visit. Cumulative overweight-years is the cumulative sum overweight-year exposure. Total cumulative overweight degree is the cumulative sum of the overweight degree exposure. Total cumulative overweight duration is the cumulative sum of the overweight duration of exposure. Abbreviations: BMI, body mass index; HRT, hormone replacement therapy; *N,* number of participants; WC, waist circumference.

a Overweight-years (kg-years/m^2^) = prior degree of overweight (kg/m^2^) × duration of overweight (years).

**Table 2:** Hazard ratio of cancers per standard deviation of overweight-years at Visit 2 and BMI at Visit 2, ARIC.

Outcomes	Number of cancer events	Overweight-years, kg-years/m^2^ (per SD)		BMI, kg/m^2^ (perSD)	
		Age-adjusted HR^a^ (95% CI)	MV-adjusted HR (95% CI)	Age-adjusted HR (95% CI)	MV-adjusted HR (95% CI)
*Men*					
All cancers	2072	1.03 (0.98–1.07)	1.03 (0.98–1.07)	1.02 (0.98–1.07)	1.02 (0.98–1.07)
OBR-cancers	408	1.15(1.05–1.25)	1.15 (1.05–1.25)	1.16 (1.05–1.27)	1.15 (1.05–1.27)
NOBR-cancers	1664	1.00 (0.95–1.05)	1.00 (0.95–1.05)	0.99 (0.94–1.04)	0.99(0.94–1.04)
NOBR-cancers excluding lung and prostate	570	1.00 (0.92–1.09)	1.01 (0.93–1.10)	1.05 (0.96–1.14)	1.05 (0.97–1.15)
Specific cancer sites					
Colorectal	175	1.32(1.18–1.47)	1.32 (1.18–1.47)	1.29(1.12–1.48)	1.28(1.12–1.47)
Kidney	67	1.00 (0.77–1.29)	0.99 (0.77–1.28)	1.03 (0.80–1.32)	1.03 (0.80–1.32)
Bladder	69	1.16(0.94–1.43)	1.17 (0.95–1.45)	1.17 (0.93–1.48)	1.18(0.93–1.49)
Pancreas	63	0.96(0.74–1.26)	0.96 (0.74–1.26)	1.13 (0.89–1.45)	1.13 (0.89–1.45)
Lung	315	0.91(0.81–1.03)	0.92 (0.82–1.04)	0.79 (0.70–0.9)	0.81 (0.72–0.92)
Prostate	779	1.02 (0.95–1.10)	1.02 (0.95–1.09)	1.03 (0.96–1.11)	1.02 (0.95–1.10)
Metastatic prostate	53	1.14 (0.89–1.46)	1.13 (0.89–1.44)	1.04(0.79–1.37)	1.04(0.79–1.36)
*Women*					
All cancers	1804	1.07(1.02–1.12)	1.08 (1.04–1.14)	1.11 (1.06–1.16)	1.14(1.08–1.19)
OBR-cancers	1120	1.14(1.08–1.20)	1.14(1.08–1.20)	1.18 (1.12–1.25)	1.19 (1.12–1.26)
NOBR-cancers	684	0.96(0.88–1.03)	0.99 (0.92–1.07)	0.99 (0.91–1.07)	1.05 (0.97–1.14)
NOBR-cancers excluding lung	457	1.00 (0.91–1.10)	1.03 (0.93–1.13)	1.06 (0.97–1.17)	1.12 (1.01–1.23)
Specific cancer sites					
Colorectal	181	1.08 (0.94–1.24)	1.04(0.90–1.21)	1.19 (1.03–1.36)	1.14 (0.98–1.32)
Pancreas	55	1.41 (1.15,1.72)	1.33 (1.08–1.65)	1.29 (1.01–1.64)	1.16(0.89–1.52)
Kidney	58	1.33(1.08–1.64)	1.29 (1.03–1.6)	1.45 (1.17–1.81)	1.40(1.10–1.78)
Lung	228	0.87 (0.75–1.00)	0.93 (0.80–1.07)	0.83 (0.72–0.97)	0.92 (0.79–1.07)
Endometrial	109	1.44(1.24–1.67)	1.51 (1.29–1.76)	1.60 (1.38–1.86)	1.75 (1.49–2.06)
Ovarian	64	1.06 (0.83–1.36)	1.12 (0.87–1.43)	1.01 (0.79–1.30)	1.10 (0.85–1.42)
Post-menopausal breast cancer	546	1.06 (0.98–1.15)	1.07 (0.99–1.16)	1.09 (1.00–1.18)	1.11(1.02–1.21)

Abbreviations: BMI, body mass index; CI, confidence interval; HR, hazard ratio; HRT, hormone replacement therapy; MV, multivariable; NOBR, non-obesity related; OBR, obesity-related; SD, standard deviation.

a Multivariable adjustment for baseline age, ethnicity, alcohol, smoking and HRT (in women).

**Table 3:** Hazard ratios of cancers per standard deviation overweight degree and duration at Visit 2, in ARIC.

Outcomes	Number of cancer events	Degree of Overweight, kg/m^2^ (per SD)		Duration of Overweight, years (per SD)	
		Age-adjusted HR^a^ (95%CI)	MV-adjusted HR (95% CI)	Age-adjusted HR (95% CI)	MV-adjusted HR (95% CI)
*Men*					
All cancers	2072	1.03 (0.97–1.09)	1.02 (0.96–1.09)	0.98(0.93–1.03)	0.99(0.93–1.04)
OBR-cancers	408	1.10 (0.97–1.25)	1.10 (0.97–1.25)	0.96 (0.85–1.09)	0.97 (0.85–1.09)
NOBR-cancers	1664	1.00 (0.94–1.08)	1.00 (0.94–1.08)	0.98(0.93–1.04)	0.99(0.93–1.06)
NOBR-cancers excluding lung and prostate	570	0.95 (0.84–1.07)	0.95 (0.84–1.08)	0.94 (0.84–1.04)	0.94 (0.85–1.04)
Specific cancer sites					
Colorectal	175	1.29 (1.11–1.51)	1.29 (1.10–1.51)	1.10 (0.92–1.32)	1.11(0.92–1.33)
Kidney	67	0.96 (0.66–1.39)	0.95 (0.65–1.38)	0.8 (0.57–1.12)	0.79 (0.56–1.11)
Bladder	69	1.10 (0.81–1.50)	1.12 (0.82–1.52)	1.03 (0.77–1.39)	1.04(0.77–1.40)
Pancreas	63	0.75 (0.48–1.17)	0.75 (0.48–1.17)	0.95(0.69–1.31)	0.96(0.70–1.33)
Lung	315	1.10 (0.95–1.27)	1.10 (0.95–1.28)	0.96(0.84–1.10)	0.98(0.86–1.13)
Prostate	779	1.00 (0.91–1.11)	1.00 (0.9–1.10)	1.03 (0.95–1.13)	1.05 (0.96–1.14)
Metastatic prostate	53	1.22 (0.88–1.69)	1.20 (0.87–1.67)	1.18(0.85–1.65)	1.20 (0.86–1.69)
*Women*					
All cancers	1804	0.99 (0.92–1.06)	0.99 (0.92–1.06)	0.98(0.92–1.04)	0.98(0.93–1.04)
OBR-cancers	1120	1.03 (0.94–1.12)	1.03 (0.94–1.12)	1.03 (0.96–1.11)	1.03 (0.96–1.12)
NOBR-cancers	684	0.93 (0.83–1.04)	0.92 (0.82–1.04)	0.91(0.83–1.00)	0.92 (0.84–1.01)
NOBR-cancers excluding lung	457	0.97 (0.79–1.18)	0.96 (0.79–1.18)	0.89 (0.76–1.05)	0.90 (0.77–1.06)
Specific cancer sites					
Colorectal	181	0.89 (0.71–1.12)	0.90 (0.72–1.13)	1.03 (0.85–1.24)	1.03 (0.85–1.24)
Pancreas	55	1.48 (1.09–2.00)	1.49 (1.10–2.02)	1.27(0.89–1.79)	1.26(0.89–1.79)
Kidney	58	1.03 (0.72–1.49)	1.05 (0.73–1.51)	1.07 (0.76–1.52)	1.08 (0.76–1.53)
Lung	228	0.97 (0.79–1.18)	0.96 (0.79–1.18)	0.89 (0.76–1.05)	0.90 (0.77–1.06)
Endometrial	109	1.02 (0.77–1.33)	1.00 (0.76–1.32)	1.05 (0.8–1.38)	1.04(0.79–1.37)
Ovarian	64	1.12 (0.78–1.60)	1.10 (0.77–1.59)	1.15(0.83–1.59)	1.15(0.83–1.59)
Post-menopausal breast cancer	546	1.00 (0.88–1.14)	1.00 (0.88–1.13)	1.02 (0.91–1.13)	1.02 (0.91–1.13)

Abbreviations: BMI, body mass index; CI, confidence interval; HR, hazard ratio; MV, multivariable; NOBR, non-obesity related; OBR, obesity-related.

a Multivariable adjustment for baseline age, ethnicity, alcohol, smoking and hormone replacement therapy (in women). Degree of overweight is the cumulative sum of the number of BMI units ≥25 kg/m^2^ over the exposure period. Duration of overweight is the cumulative sum of the duration overweight (BMI ≥25 kg/m^2^) over the exposure period.

**Table 4:** Comparison of the overweight-years metric at Visit 2 and BMI at Visit 2 using Harrell’s C- statistic, ARIC.

Characteristic	Harrell’s C-statistic (95% CI)					
	Overweight-years (kg-years/m^2^)^a^	BMI (kg/m^2^)	Difference in C-statistic between BMI and overweight-years	Degree of overweight (kg/m^2^)	Duration of overweight (years)	Difference in c-statistic between duration and degree of overweight
*Men*						
All cancers	0.603 (0.592 to 0.613)	0.603 (0.592 to 0.614)	0.000 (–0.008 to 0.008)	0.603 (0.591 to 0.615)	0.601 (0.589 to 0.613)	–0.003 (–0.012 to 0.007)
OBR-cancers	0.591 (0.573 to 0.609)	0.591 (0.574 to 0.609)	0.000 (–0.011 to 0.012)	0.590 (0.573 to 0.608)	0.5848 (0.568 to 0.603)	–0.006 (–0.018 to 0.007)
NOBR-cancers	0.606 (0.595 to 0.617)	0.607 (0.595 to 0.618)	0.001 (–0.009 to 0.011)	0.608 (0.597 to 0.619)	0.605 (0.594 to 0.616)	–0.003 (–0.008 to 0.003)
NOBR-cancers excluding lung and prostate	0.590 (0.576 to 0.605)	0.590 (0.576 to 0.605)	–0.000 (–0.004 to 0.003)	0.590 (0.576 to 0.605)	0.591 (0.576 to 0.606)	0.000 (–0.002 to 0.002)
Specific cancer sites						
Colorectal	0.658 (0.632 to 0.686)	0.641 (0.615 to 0.669)	–0.017 (–0.036 to 0.003)	0.656 (0.629 to 0.683)	0.641 (0.615 to 0.668)	–0.014 (–0.034 to 0.005)
Pancreas	0.542 (0.499 to 0.589)	0.551 (0.506 to 0.599)	0.009 (–0.021 to 0.038)	0.540 (0.497 to 0.587)	0.538 (0.495 to 0.585)	–0.003 (–0.018 to 0.013)
Kidney	0.601 (0.551 to 0.655)	0.603 (0.554 to 0.656)	0.002 (–0.020 to 0.023)	0.601 (0.551 to 0.655)	0.622 (0.574 to 0.675)	0.021 (–0.016 to 0.058)
Lung	0.723 (0.705 to 0.741)	0.731 (0.713 to 0.750)	0.008 (–0.001 to 0.018)	0.723 (0.705 to 0.742)	0.727 (0.709 to 0.746)	0.004 (–0.001 to 0.009)
Prostate	0.606 (0.593 to 0.619)	0.605 (0.592 to 0.618)	–0.001 (–0.005 to 0.003)	0.606 (0.593 to 0.619)	0.605 (0.592 to 0.619)	–0.001 (–0.006 to 0.004)
Metastatic prostate	0.596 (0.544 to 0.654)	0.587 (0.538 to 0.640)	–0.010 (–0.048 to 0.028)	0.595 (0.543 to 0.653)	0.589 (0.534 to 0.648)	–0.007 (–0.031 to 0.017)
Bladder	0.677 (0.635 to 0.721)	0.678 (0.636 to 0.723)	0.001 (–0.022 to 0.025)	0.677 (0.635 to 0.721)	0.672 (0.632 to 0.715)	–0.005 (–0.019 to 0.009)
*Women*						
All cancers	0.578 (0.568 to 0.590)	0.584 (0.573 to 0.594)	0.005 (–0.005 to 0.015)	0.581 (0.572 to 0.591)	0.579 (0.568 to 0.590)	–0.002 (–0.015 to 0.011)
OBR-cancers	0.562 (0.551 to 0.573)	0.575 (0.564 to 0.586)	0.013 (–0.000 to 0.026)	0.563 (0.552 to 0.574)	0.563 (0.553 to 0.574)	–0.000 (–0.012 to 0.011)
NOBR-cancers	0.636 (0.622 to 0.651)	0.636 (0.622 to 0.651)	0.000 (–0.010 to 0.000)	0.635 (0.621 to 0.650)	0.634 (0.620 to 0.648)	–0.001 (–0.009 to 0.007)
NOBR-cancers excluding lung	0.592 (0.576 to 0.609)	0.598 (0.582 to 0.615)	0.006 (–0.004 to 0.016)	0.593 (0.576 to 0.610)	0.593 (0.576 to 0.609)	–0.000 (–0.005 to 0.005)
Specific cancer sites						
Colorectal	0.575 (0.549 to 0.601)	0.586 (0.561 to 0.612)	0.011 (–0.011 to 0.033)	0.575 (0.550 to 0.602)	0.581 (0.556 to 0.608)	0.006 (–0.011 to 0.023)
Pancreas	0.653 (0.600 to 0.710)	0.637 (0.584 to 0.694)	–0.016 (–0.047 to 0.015)	0.652 (0.600 to 0.709)	0.626 (0.579 to 0.678)	–0.026 (–0.058 to 0.006)
Kidney	0.652 (0.597 to 0.711)	0.663 (0.611 to 0.720)	0.012 (–0.025 to 0.048)	0.652 (0.598 to 0.712)	0.664 (0.615 to 0.717)	0.012 (–0.033 to 0.056)
Lung	0.754 (0.733 to 0.775)	0.753 (0.733 to 0.775)	–0.001 (–0.004 to 0.003)	0.754 (0.733 to 0.775)	0.753 (0.732 to 0.774)	–0.002 (–0.006 to 0.003)
Endometrial	0.647 (0.612 to 0.685)	0.669 (0.631 to 0.708)	0.021 (–0.015 to 0.057)	0.649 (0.614 to 0.687)	0.624 (0.592 to 0.657)	–0.026 (–0.055 to 0.004)
Ovarian	0.575 (0.534 to 0.620)	0.563 (0.523 to 0.606)	–0.012 (–0.047 to 0.023)	0.575 (0.533 to 0.619)	0.587 (0.547 to 0.630)	0.012 (–0.022 to 0.047)
Post-menopausal breast cancer	0.581 (0.566 to 0.596)	0.590 (0.576 to 0.605)	0.009 (–0.005 to 0.023)	0.582 (0.568 to 0.597)	0.580 (0.565 to 0.595)	–0.002 (–0.014 to 0.009)

Abbreviations: BMI, body mass index; Cl, confidence interval; MV, multivariable-adjusted; NOBR, non-obesity related; OBR, obesity-related; SE, standard error.

a Multivariable adjustment for baseline age, ethnicity, alcohol, smoking and hormone replacement therapy (in women).

## Data Availability

ARIC data can be requested via: https://sites.cscc.unc.edu/aric/ distribution-agreements. Further information is available from the corresponding author upon request.

## What’s new?

While obesity is associated with multiple cancer types, little is known about whether the duration of exposure to excess adipose impacts the development of obesity-related cancers. Here, the authors derived body mass index (BMI) cumulative exposure taking into account duration and degree of excess BMI. Comparison of the overweight-years metric to single BMI measures showed that cancer incidence is related to the cumulative degree and duration of overweight. Single BMI measures, however, were generally superior to the overweight-years metric in association performance characteristics. Nonetheless, the findings suggest that duration and degree of overweight should be considered in cancer prevention.
