# Supplementary material for: Overweight-years and cancer risk: A prospective study of the association and comparison of predictive performance with body mass index (Atherosclerosis Risk in Communities Study)
Source: Int J Cancer. Author manuscript; Available in PMC 2024 May 1. (PMC7615716; doi:10.1002/ijc.34821)
Supplement: Data S1. Supporting Information. [file EMS193018-supplement-Data_S1__Supporting_Information_.pdf]

Overweight-years and cancer risk: A prospective study of the association and comparison of predictive performance with body mass index (Atherosclerosis Risk in Communities Study)

Nadin K. Hawwash, Matthew Sperrin, Glen P. Martin, Corinne E. Joshu, Roberta Florido, Elizabeth A. Platz, Andrew G. Renehan

Table of contents

Table S1: Example calculation of overweight-years for one individual.

Table S2: Example calculation of obese-years for one individual.

Table S3: Strengthening the Reporting of Observational studies in Epidemiology (STROBE) guidelines checklist.

Table S4: Summary of the exposure metrics.

Table S5: Incidence of cancer (events/1000 Person-Years) according to overweight-years at Visit 2 and BMI at Visit 2 stratified by sex, race, smoking and HRT (women only) in the ARIC cohort.

Table S6: Hazard ratio of cancers per standard deviation of overweight-years at Visit 2 and BMI at Visit 2 with additional multivariable adjustments, ARIC.

Table S7: Hazard ratio of specific cancers per 100 overweight-years and per 5-unit (kg/m2) BMI at Visit 2 in the ARIC cohort.

Table S8: Hazard ratio of cancers by overweight degree and duration per 10 kg/m2 and per 10 years, respectively at Visit 2 in the ARIC cohort.

Table S9: Comparison of associations of overweight-years at Visit 2 and BMI at Visit 2 with cancer by Akaike information criterion in the ARIC cohort.

Table S10: Example of (a) apparent and (b) bootstrapped C-statistic performance estimates.

Table S11: Comparison of the overweight-years metric at Visit 2 and BMI at Visit 2 using Harrell's C-statistic, ARIC.

Table S12: Incidence of cancer (events/1000 Person-Years) according to obese-years at Visit 2 and BMI at Visit 2 by sex, race, smoking, HRT (women only) in the ARIC cohort.

Table S13: Hazard ratio of cancers by obese-years at Visit 2 and BMI at Visit 2 in ARIC.

Table S14: Hazard ratio of cancers by obese-years per 100 kg-years/m2at Visit 2 and BMI per 5 kg/m2 at Visit 2 in ARIC.

Table S15: Hazard ratio of cancers by obese degree and duration at Visit 2, ARIC.

Table S16: Hazard ratio of cancers by obesity degree and duration per 10 kg/m2 and per 10 years, respectively at Visit 2, ARIC.

Table S17: Comparison of associations of obese-years at Visit 2 and BMI at Visit 2 with cancer by Akaike information criterion in the ARIC cohort.

Table S18: Comparison of the obese-years metric at Visit 2 and BMI at Visit 2 using Harrell's C-statistic, ARIC.

Table S19: Summary of the exposure metrics.

Table S20: Incidence of cancer (events/1000 Person-Years) according to overweight years at Visit 2 and BMI at Visit 2 stratified by sex, race, smoking, HRT (women only) in the ARIC cohort.

Table S21: Hazard ratio of cancers per standard deviation of overweight-years at Visit 2 and BMI at Visit 2, ARIC.

Table S22: Hazard ratio of specific cancers per 100 overweight-years and per 5-unit (kg/m2) BMI in the ARIC cohort.

Table S23: Hazard ratios of cancers per standard deviation overweight degree and duration at Visit 2, in ARIC**.**

Table S24: Hazard ratio of cancers by overweight degree and duration per 10 kg/m2 and per 10 years, respectively at Visit 2, ARIC.

Table S25: Comparison of associations of overweight-years at Visit 2 and BMI at Visit 2 with cancer by Akaike information criterion in the ARIC cohort.

Table S26: Comparison of the overweight-years metric at Visit 2 and BMI at Visit 2 using Harrell's C-statistic, ARIC.

Table S27: Incidence of cancer (events/1000 Person-Years) according to obese-years at Visit 2 and BMI at Visit 2 by sex, race, smoking, HRT (women only) in the ARIC cohort.

Table S28: Hazard ratio of cancers by obese-years at Visit 2 and BMI at Visit 2 in ARIC.

Table S29: Hazard ratio of cancers by obese-years per 100 kg-years/m2at Visit 2 and BMI per 5 kg/m2 at Visit 2 in ARIC.

Table S30: Hazard ratio of cancers by obesity degree and duration at Visit 2, ARIC.

Table S31: Hazard ratio of cancers by obesity degree and duration per 10 kg/m2 and per 10 years, respectively at Visit 2, ARIC.

Table S32: Comparison of associations of obese-years at Visit 2 and BMI at Visit 2 with cancer by Akaike information criterion in the ARIC cohort.

Table S33: Comparison of the obese-years metric at Visit 2 and BMI at Visit 2 using Harrell's C-statistic, ARIC.

Table S34: Summary of the exposure metrics.

Table S35: Incidence of cancer (events/1000 Person-Years) according to overweight years at Visit 2 and BMI at Visit 2 stratified by sex, race, smoking, and HRT (women only) in the ARIC cohort.

Table S36: Hazard ratio of cancers per standard deviation of overweight-years at Visit 2 and BMI at Visit 2, ARIC.

Table S37: Hazard ratio of specific cancers per 100 overweight-years and per 5-unit (kg/m2) BMI in the ARIC cohort.

Table S38: Hazard ratios of cancers per standard deviation overweight degree and duration at Visit 2, in ARIC.

Table S39: Hazard ratio of cancers by overweight degree and duration per 10 kg/m2 and per 10 years, respectively at Visit 2, ARIC.

Table S40: Comparison of associations of overweight-years at Visit 2 and BMI at Visit 2 with cancer by Akaike information criterion in the ARIC cohort.

Table S41: Comparison of the overweight-years metric at Visit 2 and BMI at Visit 2 using Harrell's C-statistic, ARIC.

Table S42: Incidence of cancer (events/1000 Person-Years) according to obese-years at Visit 2 and BMI at Visit 2 by sex, race, smoking, HRT (women only) in the ARIC cohort.

Table S43: Hazard ratio of cancers by obese-years at Visit 2 and BMI at Visit 2 in ARIC.

Table S44: Hazard ratio of cancers by obese-years per 100 kg-years/m2 at Visit 2 and BMI per 5 kg/m2 at Visit 2 in ARIC

Table S45: Hazard ratio of cancers by obesity degree and duration at Visit 2, ARIC.

Table S46: Hazard ratio of cancers by obesity degree and duration per 10 kg/m2 and per 10 years, respectively at Visit 2, ARIC.

Table S47: Comparison of associations of obese-years at Visit 2 and BMI at Visit 2 with cancer by Akaike information criterion in the ARIC cohort.

Table S48: Comparison of obese-years at Visit 2 and BMI at Visit 2 using Harrell's C-statistic.

Figure S1: Diagram of the exposure and cancer follow-up period of this study.

Table S1: Example calculation of overweight-years for one individual.

Age (years)	Interval (years)	Predicted BMI measurements (kg/m^2)	Degree of overweight (kg/m^2)	Duration of overweight (years)	Overweight-years (kg-years/m^2)	Cumulative overweight years (kg-years/m^2)	Cumulative overweight degree (kg/m^2)	Cumulative overweight duration (years)
25	1.00	25.48	0.58	1.00	*NA*	0.00	0.58	1.00
26	1.00	25.79	0.89	1.00	0.58	0.58	1.47	2.00
27	1.00	25.96	1.06	1.00	0.89	1.47	2.53	3.00
28	1.00	27.05	2.15	1.00	1.06	2.53	4.68	4.00

Example calculation of overweight-years at age 27 = prior degree of overweight (25.79 – 24.90 = 0.89) x duration of overweight (1) = (0.89 x 1) = 0.89 overweight-years. Cumulative overweight-years is the cumulative sum overweight-year exposure. Cumulative overweight degree is the cumulative sum overweight degree exposure and overweight-duration is the cumulative sum overweight duration.

Abbreviation: BMI, body mass index.

NB: This is only a simplified example calculation of the metric and is only shown for ages 25 to 28 for ease of understanding. In this study BMI is predicted per year from age 25 and the metric is calculated from age 25 till Visit 2.

Table S2: Example calculation of obese-years for one individual.

Age (years)	Interval (years)	Predicted BMI measurements (kg/m^2)	Degree of obesity (kg/m^2)	Duration of obesity (years)	Obese-years (kg-years/m^2)	Cumulative obese-years(kg-years/m^2)	Cumulative degree of obesity (kg/m^2)	Cumulative duration of obesity (years)
25	1.00	27.37	0.00	0.00	*NA*	0.00	0.00	0.00
26	1.00	32.33	2.43	1.00	0.00	0.00	2.43	1.00
27	1.00	31.99	2.09	1.00	2.43	2.43	4.52	2.00
28	1.00	33.46	3.56	1.00	2.09	4.52	8.08	3.00

Example calculation of obese-years at age 27 = prior degree of obesity (32.33 – 29.90 = 2.43) x duration of obesity in years (1.00) = (2.43x1.00) = 2.43 obese-years. Cumulative obese-years is the cumulative sum obese-year exposure. Cumulative degree of obesity is the cumulative sum of the degree of obesity and cumulative duration of obesity is the cumulative sum duration of obesity.

Abbreviation: BMI, body mass index.

NB: This is only a simplified example calculation of the metric and is only shown for ages 25 to 28 for ease of understanding. In this study BMI is predicted per year from age 25 and the metric is calculated from age 25 till Visit 2.

Table S3: Strengthening the Reporting of Observational studies in Epidemiology (STROBE) guidelines checklist.

	Item No.	Recommendation	Page No.	Relevant text from manuscript
**Title and abstract**	1	(*a*) Indicate the study's design with a commonly used term in the title or the abstract	2	Title: " Overweight-years and cancer risk: A prospective study of the association and comparison of predictive performance with body mass index (Atherosclerosis Risk in Communities Study)"
		(*b*) Provide in the abstract an informative and balanced summary of what was done and what was found	3	"BMI) is associated with a higher risk of at least 13 cancers, but it is usually measured at a single time point. We tested whether the overweight-years metric, which incorporates exposure time to BMI ≥25 kg/m^2, is associated with cancer risk and compared this with a single BMI measure."
**Introduction**				
Background/rationale	2	Explain the scientific background and rationale for the investigation being reported	4	"To optimize and target obesity intervention programs, there is a need to better understand when and for how long exposure to excess adipose is most relevant to the development of obesity-related cancer.."
Objectives	3	State specific objectives, including any prespecified hypotheses	4	"Here, we used the Atherosclerosis Risk in Communities Study (ARIC) to i) evaluate the association between the overweight-year metric at the start of follow-up and cancer incidence, including the components of the metric (degree and duration), and ii) compare the predictive performance

				of the overweight-year metric with that for BMI measured once also at the start of follow up. This study is part of a larger ABACus 2 consortium project (12)."
**Methods**				
Study design	4	Present key elements of study design early in the paper	5	Section 2 METHODS: Study population and data
Setting	5	Describe the setting, locations, and relevant dates, including periods of recruitment, exposure, follow-up, and data collection	5-6	Under Methods section
Participants	6	(*a*) *Cohort study*—Give the eligibility criteria, and the sources and methods of selection of participants. Describe methods of follow-up<br>*Case-control study*—Give the eligibility criteria, and the sources and methods of case ascertainment and control selection. Give the rationale for the choice of cases and controls<br>*Cross-sectional study*—Give the eligibility criteria, and the sources and methods of selection of participants	5-7	
		(*b*) *Cohort study*—For matched studies, give matching criteria and number of exposed and unexposed<br>*Case-control study*—For matched studies, give matching criteria and the number of controls per case	NA	
Variables	7	Clearly define all outcomes, exposures, predictors, potential confounders, and effect modifiers. Give diagnostic criteria, if applicable	5,6	
Data sources/ measurement	8*	For each variable of interest, give sources of data and details of methods of assessment (measurement). Describe	*5*	**2 METHODS**:<br>**Study population and data**

		comparability of assessment methods if there is more than one group		**2.1 Exposure**
Bias	9	Describe any efforts to address potential sources of bias	8	“The subgroup with 1 BMI reading was modelled to identify any selection bias towards healthier individuals..”
Study size	10	Explain how the study size was arrived at	6	“Other outcomes of interest included cancer-specific sites of those with at least 10 events per candidate predictor parameter (EPP)”

Continued on next page

Quantitative variables	11	Explain how quantitative variables were handled in the analyses. If applicable, describe which groupings were chosen and why		"
Statistical methods	12	(*a*) Describe all statistical methods, including those used to control for confounding	6,7	Statistical analysis section
		(*b*) Describe any methods used to examine subgroups and interactions	8	Sensitivity analysis section
		(*c*) Explain how missing data were addressed	6,7	"To account for missing covariate data, missing values were imputed by multiple imputation"
		(*d*) *Cohort study*—If applicable, explain how loss to follow-up was addressed<br>*Case-control study*—If applicable, explain how matching of cases and controls was addressed<br>*Cross-sectional study*—If applicable, describe analytical methods taking account of sampling strategy	5	"Participants were followed up until cancer diagnosis, death, or administrative censoring on December 31, 2015."
		(*e*) Describe any sensitivity analyses		
**Results**				
Participants	13*	(a) Report numbers of individuals at each stage of study—eg numbers potentially eligible, examined for eligibility, confirmed eligible, included in the study, completing follow-up, and analysed	7	Figure 1
		(b) Give reasons for non-participation at each stage	7	Figure 1
		(c) Consider use of a flow diagram	7	Figure 1
Descriptive data	14*	(a) Give characteristics of study participants (eg demographic, clinical, social) and information on exposures and potential confounders	7	
		(b) Indicate number of participants with missing data for each variable of interest	7	Table 1
		(c) *Cohort study*—Summarise follow-up time (eg, average and total amount)	7	"A total of 2,072 cancers were diagnosed in men and 1,804 were diagnosed in women over a mean follow-up period of 18 years (SD 8) in men and 20 years (SD 7) in women (Table S3)."
Outcome data	15*	*Cohort study*—Report numbers of outcome events or summary measures over time	*8*	A total of 2,072 cancers were diagnosed in men and 1,804 were diagnosed in women over a

				mean follow-up period of 18 years (SD 8) in men and 20 years (SD 7) in women (Table S3).
		Case-control study—Report numbers in each exposure category, or summary measures of exposure		*NA*
		Cross-sectional study—Report numbers of outcome events or summary measures		*NA*
Main results	16	(*a*) Give unadjusted estimates and, if applicable, confounder-adjusted estimates and their precision (eg, 95% confidence interval). Make clear which confounders were adjusted for and why they were included	12,14	Table 2,3
		(*b*) Report category boundaries when continuous variables were categorized		Per standard deviation exposure was used.
		(*c*) If relevant, consider translating estimates of relative risk into absolute risk for a meaningful time period		Incidence rates compared in supplementary table.

Other analyses	17	Report other analyses done—eg analyses of subgroups and interactions, and sensitivity analyses	6	Under heading sensitivity analysis
**Discussion**				
Key results	18	Summarise key results with reference to study objectives	8-18	
Limitations	19	Discuss limitations of the study, taking into account sources of potential bias or imprecision. Discuss both direction and magnitude of any potential bias	20	**7 Limitations**
Interpretation	20	Give a cautious overall interpretation of results considering objectives, limitations, multiplicity of analyses, results from similar studies, and other relevant evidence	20,21	
Generalisability	21	Discuss the generalisability (external validity) of the study results	20	"A limitation of this study was the use of BMI data from 1987-1998 which may not be generalisable to the current population given the worldwide rise and earlier onset of obesity over the last 4-5 decades"
**Other information**				

Funding	22	Give the source of funding and the role of the funders for the present study and, if applicable, for the original study on which the present article is based	13	Under heading funding

*Give information separately for cases and controls in case-control studies and, if applicable, for exposed and unexposed groups in cohort and cross-sectional studies.

Note: An Explanation and Elaboration article discusses each checklist item and gives methodological background and published examples of transparent reporting. The STROBE checklist is best used in conjunction with this article (freely available on the Web sites of PLoS Medicine at http://www.plosmedicine.org/, Annals of Internal Medicine at http://www.annals.org/, and Epidemiology at http://www.epidem.com/). Information on the STROBE Initiative is available at www.strobe-statement.org.

<u>Analysis using BMI predicted from the at least 3 observed BMI readings subgroup</u>

a) **Analysis of overweight-years exposure**

Table S4: Summary of the exposure metrics.

Characteristic	Men
**Baseline BMI, (kg/m^2)**	27.70 (4.30)
**End of cancer follow up, (years)**	18.00 (8.00)
**Total cumulative overweight years, ($kg\text{-}years/m^2$)**	50.00 (65.00)
**Total cumulative overweight degree, (kg/m^2)**	53.00 (67.00)
**Total cumulative overweight duration, (years)**	16.00 (12.00)
**Total cumulative obese-years, ($kg\text{-}years/m^2$)**	8.00 (26.00)
**Total cumulative obese-degree, (kg/m^2)**	9.00 (27.00)
**Total cumulative obese-duration, (years)**	3.00 (7.00)
	**Women**
**Baseline BMI, (kg/m^2)**	28.20 (6.10)
**End of cancer follow up, (years)**	20.00 (7.00)
**Total cumulative overweight years, ($kg\text{-}years/m^2$)**	84.00 (100.00)
**Total cumulative overweight degree, (kg/m^2)**	89.00 (104.00)
**Total cumulative overweight duration, (years)**	19.00 (13.00)
**Total cumulative obese-years, ($kg\text{-}years/m^2$)**	24.00 (55.00)
**Total cumulative obese-degree, (kg/m^2)**	26.00 (57.00)
**Total cumulative obese-duration, (years)**	7.00 (10.00)

Mean (SD)

Baseline refers to Visit 2.

Abbreviations: N = number of participants; SD, standard deviation; BMI, body mass index

Table S5: Incidence of cancer (events/1000 Person-Years) according to overweight years at Visit 2 and BMI at Visit 2 stratified by gender, ethnicity, smoking, HRT (women only) in the ARIC cohort.

	Men											
	**0 overweight-years (kg-years/m^2)**			**>0-100 overweight-years (kg-years/m^2)**			**>100 overweight-years (kg-years/m^2)**			**Baseline BMI (kg /m^2)**		
	**N**	**PYFU**	**IR (95% CI)**	**N**	**PYFU**	**IR (95% CI)**	**N**	**PYFU**	**IR (95% CI)**	**N**	**PYFU**	**IR (95% CI)**
**Whole sample**	424	49885.85	8.50 (7.68, 9.32)	1277	163879.90	7.79 (7.36, 8.22)	371	40775.47	9.10 (8.16, 10.04)	2072.00	254541.30	8.14 (7.79, 8.49)
Ethnicity												
**White**	320	40252.53	7.95 (7.07, 8.83)	1008	133736.70	7.54 (7.07, 8.01)	279	32568.73	8.57 (7.55, 9.59)	1607.00	206558.00	7.78 (7.4, 8.16)
**Black**	104	9633.32	10.80 (8.67, 12.93)	269	30143.21	8.92 (7.84, 10.01)	92	8206.74	11.21 (8.85, 13.57)	465.00	47983.27	9.69 (8.80, 10.58)
**Smoking**												
**Ever**	338	37034.14	9.12 (8.14, 10.11)	957	118252.00	8.10 (7.58, 8.62)	270	29214.62	9.24 (8.12, 10.36)	1565.70	184500.80	8.49 (8.06, 8.91)
**Never**	86	12851.71	6.70 (5.24, 8.16)	319	45627.92	7.00 (6.22, 7.77)	101	11560.85	8.74 (6.99, 10.49)	506.30	70040.48	7.23 (6.59, 7.87)
	**Women**											
**Whole sample**	358	75731.92	4.73 (4.23, 5.22)	818	167807.30	4.87 (4.54, 5.21)	628	114853.40	5.47 (5.04, 5.90)	1804.00	358392.70	5.03 (4.80, 5.27)
**Ethnicity**												
**White**	314	66950.57	4.69 (4.16, 5.22)	617	121641.00	5.07 (4.67, 5.48)	405	73320.17	5.52 (4.98, 6.07)	1336.00	261911.80	5.10 (4.83, 5.38)
**Black**	44	8781.36	5.01 (3.47, 6.55)	201	46166.29	4.35 (3.74, 4.97)	223	41533.26	5.37 (4.65, 6.09)	468.00	96480.90	4.85 (4.41, 5.30)
**Smoking**												
**Ever**	228	41404.69	5.51 (4.78, 6.23)	452	82455.18	5.48 (4.97, 5.99)	304	50090.16	6.07 (5.37, 6.76)	983.80	173950.00	5.66 (5.30, 6.01)
**Never**	130	34327.23	3.79 (3.12, 4.45)	366	85352.15	4.29 (3.84, 4.73)	324	64763.26	5.01 (4.45, 5.56)	820.20	184442.60	4.45 (4.14, 4.75)
**HRT**												
**Ever**	155	33796.60	4.58 (3.84, 5.32)	325	63649.63	5.11 (4.55, 5.67)	148	31940.43	4.64 (3.87, 5.4)	628.00	129386.70	4.85 (4.47, 5.24)
**Never**	203	41935.33	4.85 (4.17, 5.52)	492	104157.70	4.73 (4.31, 5.15)	480	82912.99	5.79 (5.26, 6.31)	1176.00	229006.00	5.13 (4.84, 5.43)

Abbreviations: N, number of cancer events; PYFR, person-years of cancer follow-up; IR, incidence rate of all cancers; BMI, body mass index.

Table S6: Hazard ratio of cancers per standard deviation of overweight-years at Visit 2 and BMI at Visit 2 with additional multivariable adjustments, ARIC.

Outcomes	Number of cancer events	Overweight-years (per SD) (kg-years/m^2)		BMI (per SD) (kg/m^2)	
		Age-adjusted HR (95% CI)	MV-adjusted HR (95% CI)	Age-adjusted HR (95% CI)	MV-adjusted HR (95% CI)
**Men**					
All Cancers	2,072	1.03 (0.98,1.07)	1.04 (0.99,1.08)	1.02 (0.98,1.07)	1.02 (0.98,1.07)
OBR-cancers	408	1.15 (1.05,1.25)	1.17 (1.07,1.27)	1.16 (1.05,1.27)	1.15 (1.05,1.27)
NOBR-cancers	1,664	1.00 (0.95,1.05)	1.00 (0.95,1.05)	0.99 (0.94,1.04)	0.99 (0.94,1.05)
NOBR-cancers excluding lung and prostate	570	1.00 (0.92,1.09)	1.02 (0.93,1.11)	1.05 (0.96,1.14)	1.05 (0.96,1.14)
**Specific cancer sites**					
Colorectal	175	1.32 (1.18,1.47)	1.34 (1.19,1.50)	1.29 (1.12,1.48)	1.28 (1.12,1.47)
Kidney	67	1.00 (0.77,1.29)	1.00 (0.78,1.30)	1.03 (0.80,1.32)	1.03 (0.80,1.32)
Bladder	69	1.16 (0.94,1.43)	1.21 (0.97,1.50)	1.17 (0.93,1.48)	1.17 (0.92,1.49)
Pancreas	63	0.96 (0.74,1.26)	1.00 (0.76,1.31)	1.13 (0.89,1.45)	1.13 (0.89,1.45)
Lung	315	0.91 (0.81,1.03)	0.93 (0.82,1.05)	0.79 (0.70,0.90)	0.82 (0.72,0.93)
Prostate	779	1.02 (0.95,1.10)	1.02 (0.95,1.10)	1.03 (0.96,1.11)	1.02 (0.95,1.10)
Metastatic Prostate	53	1.14 (0.89,1.46)	1.12 (0.87,1.43)	1.04 (0.79,1.37)	1.03 (0.79,1.35)
**Women**					
All Cancers	1,804	1.07 (1.02,1.12)	1.11 (1.06,1.17)	1.11 (1.06,1.16)	1.13 (1.07,1.20)
OBR-cancers	1,120	1.14 (1.08,1.20)	1.20 (1.12,1.28)	1.18 (1.12,1.25)	1.19 (1.11,1.28)
NOBR-cancers	684	0.96 (0.88,1.03)	0.97 (0.89,1.07)	0.99 (0.91,1.07)	1.04 (0.94,1.15)
NOBR-cancers excluding lung	457	1.00 (0.91,1.10)	1.00 (0.9,1.12)	1.06 (0.97,1.17)	1.11 (0.99,1.24)
**Specific cancer sites**					
Colorectal	181	1.08 (0.94,1.24)	1.09 (0.93,1.29)	1.19 (1.03,1.36)	1.10 (0.92,1.32)
Pancreas	55	1.41 (1.15,1.72)	1.46 (1.15,1.85)	1.29 (1.01,1.64)	1.19 (0.87,1.62)
Kidney	58	1.33 (1.08,1.64)	1.34 (1.04,1.72)	1.45 (1.17,1.81)	1.42 (1.08,1.87)
Lung	228	0.87 (0.75,1.00)	0.91 (0.77,1.08)	0.83 (0.72,0.97)	0.89 (0.75,1.07)
Endometrial	109	1.44 (1.24,1.67)	1.57 (1.28,1.91)	1.60 (1.38,1.86)	1.70 (1.37,2.11)
Ovarian	64	1.06 (0.83,1.36)	1.09 (0.81,1.46)	1.01 (0.79,1.3)	1.03 (0.75,1.42)
Post-menopausal breast cancer	546	1.06 (0.98,1.15)	1.14 (1.04,1.26)	1.09 (1.00,1.18)	1.16 (1.04,1.29)

* Multivariable adjustment for baseline age, ethnicity, alcohol, smoking, pack-years and height in men and baseline age, ethnicity, alcohol, HRT, smoking, pack-years, height, age of menopause and age of menarche in women.

Abbreviations: OBR, obesity-related; NOBR, non-obesity related; CI, confidence interval; HR, hazard ratio; BMI, body mass index; MV, multivariable; SD, standard deviation; HRT, hormone replacement therapy.

Table S7: Hazard ratio of specific cancers per 100 overweight-years and per 5-unit (kg/m^2) BMI at Visit 2 in the ARIC cohort.

Outcomes	Men			
	Overweight-years (per 100 kg-years/m^2)		BMI (per 5 unit [kg/m^2])	
	Age-adjusted HR (95% CI)	MV-adjusted HR (95% CI)	Age-adjusted HR (95% CI)	MV-adjusted HR (95% CI)
All Cancers	1.04 (0.98,1.12)	1.04 (0.98,1.12)	1.03 (0.97,1.08)	1.03 (0.98,1.08)
OBR-cancers	1.24 (1.09,1.42)	1.24 (1.08,1.42)	1.19 (1.06,1.33)	1.18 (1.06,1.33)
NOBR-cancers	0.99 (0.92,1.07)	1.00 (0.92,1.08)	0.99 (0.93,1.05)	0.99 (0.93,1.05)
NOBR-cancers excluding lung and prostate	1.00 (0.88,1.15)	1.02 (0.89,1.16)	1.05 (0.95,1.17)	1.06 (0.96,1.18)
**Specific cancer sites**				
Colorectal	1.55 (1.30,1.84)	1.54 (1.30,1.84)	1.35 (1.15,1.59)	1.34 (1.14,1.58)
Kidney	1.00 (0.67,1.49)	0.99 (0.66,1.48)	1.04 (0.77,1.39)	1.03 (0.77,1.39)
Bladder	1.26 (0.91,1.75)	1.29 (0.92,1.8)	1.20 (0.91,1.59)	1.21 (0.92,1.60)
Pancreas	0.94 (0.62,1.43)	0.94 (0.62,1.43)	1.16 (0.87,1.55)	1.16 (0.87,1.55)
Lung	0.86 (0.72,1.04)	0.88 (0.73,1.07)	0.76 (0.65,0.88)	0.78 (0.67,0.90)
Prostate	1.04 (0.93,1.16)	1.02 (0.92,1.14)	1.04 (0.95,1.13)	1.03 (0.95,1.12)
Metastatic prostate	1.23 (0.84,1.81)	1.22 (0.83,1.78)	1.05 (0.76,1.44)	1.04 (0.76,1.43)

Women				
All Cancers	1.07 (1.02,1.12)	1.08 (1.04,1.14)	1.09 (1.05,1.13)	1.11 (1.07,1.16)
OBR-cancers	1.14 (1.08,1.20)	1.14 (1.08,1.20)	1.14 (1.09,1.20)	1.15 (1.10,1.21)
NOBR-cancers	0.96 (0.88,1.03)	0.99 (0.92,1.08)	0.99 (0.93,1.05)	1.04 (0.97,1.11)
NOBR-cancers excluding lung	1.00 (0.91,1.10)	1.03 (0.93,1.13)	1.05 (0.98,1.14)	1.09 (1.01,1.19)
**Specific cancer sites**				
Colorectal	1.08 (0.94,1.24)	1.04 (0.90,1.21)	1.15 (1.03,1.29)	1.11 (0.98,1.26)
Pancreas	1.41 (1.15,1.73)	1.33 (1.08,1.65)	1.23 (1.01,1.50)	1.13 (0.91,1.41)
Kidney	1.33 (1.08,1.64)	1.29 (1.03,1.6)	1.36 (1.14,1.63)	1.32 (1.09,1.60)
Lung	0.87 (0.75,1.00)	0.93 (0.80,1.07)	0.86 (0.76,0.97)	0.93 (0.82,1.06)
Endometrial	1.44 (1.25,1.68)	1.51 (1.29,1.76)	1.47 (1.30,1.66)	1.59 (1.39,1.81)
Ovarian	1.06 (0.83,1.36)	1.12 (0.87,1.43)	1.01 (0.82,1.24)	1.08 (0.87,1.34)
Post-menopausal breast cancer	1.06 (0.98,1.15)	1.07 (0.99,1.17)	1.07 (1.00,1.14)	1.09 (1.01,1.17)

* Multivariable adjustment for baseline age, ethnicity, alcohol, smoking and HRT (in women).
Abbreviations: OBR, obesity-related; NOBR, non-obesity related; CI, confidence interval; HR, hazard ratio; BMI, body mass index; MV, multivariable.

Table S8: Hazard ratio of cancers by overweight degree and duration per 10 units and per 10 years, respectively at Visit 2 in the ARIC cohort.

Outcome	Degree of Overweight (per 10 kg/m^2)		Duration of Overweight (per 10 years)	
	Age-adjusted HR (95% CI)	MV-adjusted HR (95% CI)	Age-adjusted HR (95% CI)	MV-adjusted HR (95% CI)
**Men**				
All Cancers	1.00 (0.99,1.01)	1.00 (0.99,1.01)	0.98 (0.94,1.03)	0.99 (0.94,1.04)
OBR-cancers	1.01 (1.00,1.03)	1.01 (1.00,1.03)	0.97 (0.87,1.07)	0.97 (0.87,1.08)
NOBR-cancers	1.00 (0.99,1.01)	1.00 (0.99,1.01)	0.99 (0.94,1.04)	0.99 (0.94,1.05)
NOBR-cancers excluding lung and prostate	0.99 (0.97,1.01)	0.99 (0.97,1.01)	0.95 (0.87,1.04)	0.95 (0.87,1.04)
**Specific cancer sites**				
Colorectal	1.04 (1.02,1.06)	1.04 (1.02,1.06)	1.08 (0.93,1.26)	1.09 (0.93,1.27)
Kidney	0.99 (0.94,1.05)	0.99 (0.94,1.05)	0.82 (0.62,1.10)	0.82 (0.61,1.09)
Bladder	1.01 (0.97,1.06)	1.02 (0.97,1.07)	1.03 (0.80,1.32)	1.03 (0.80,1.33)
Pancreas	0.96 (0.89,1.02)	0.96 (0.89,1.02)	0.96 (0.73,1.26)	0.97 (0.74,1.27)
Lung	1.01 (0.99,1.04)	1.01 (0.99,1.04)	0.97 (0.86,1.09)	0.98 (0.88,1.10)
Prostate	1.00 (0.99,1.02)	1.00 (0.98,1.01)	1.03 (0.95,1.11)	1.04 (0.96,1.12)
Metastatic Prostate	1.03 (0.98,1.08)	1.03 (0.98,1.08)	1.15 (0.87,1.53)	1.17 (0.88,1.56)
**Women**				
All Cancers	1.01 (0.99,1.02)	1.00 (0.99,1.02)	1.10 (0.98,1.24)	1.07 (0.95,1.21)
OBR-cancers	1.04 (1.02,1.05)	1.04 (1.03,1.06)	1.34 (1.12,1.59)	1.37 (1.15,1.63)
NOBR-cancers	1.03 (1.01,1.05)	1.03 (1.00,1.05)	1.26 (1.01,1.57)	1.22 (0.97,1.53)
NOBR-cancers excluding lung	0.99 (0.97,1.00)	0.99 (0.98,1.01)	0.87 (0.79,0.97)	0.92 (0.83,1.01)
**Specific cancer sites**				
Colorectal	1.01 (0.98,1.03)	1.01 (0.99,1.04)	1.08 (0.88,1.32)	1.12 (0.91,1.38)
Pancreas	1.03 (1.01,1.05)	1.03 (1.01,1.05)	1.28 (1.02,1.60)	1.21 (0.96,1.53)
Kidney	1.01 (1.00,1.01)	1.01 (1.00,1.02)	1.05 (0.98,1.12)	1.06 (0.98,1.13)
Lung	1.01 (0.99,1.02)	1.00 (0.99,1.02)	1.10 (0.98,1.24)	1.07 (0.95,1.21)
Endometrial	1.04 (1.02,1.05)	1.04 (1.03,1.06)	1.34 (1.12,1.59)	1.37 (1.15,1.63)
Ovarian	1.03 (1.01,1.05)	1.03 (1.00,1.05)	1.26 (1.01,1.57)	1.22 (0.97,1.53)
Post-menopausal breast cancer	0.99 (0.97,1.00)	0.99 (0.98,1.01)	0.87 (0.79,0.97)	0.92 (0.83,1.01)

* Multivariable adjustment for baseline age, ethnicity, alcohol, smoking and HRT (in women).
* Degree of overweight is the cumulative sum of the number of BMI units ≥ 25 kg/m^2 over the exposure period.
* Duration of overweight is the cumulative sum of the duration overweight (BMI ≥ 25 kg/m^2) over the exposure period.
Abbreviations: OBR, obesity-related; NOBR, non-obesity related; CI, confidence interval; HR, hazard ratio; BMI, body mass index; MV, multivariable.

Table S9: Comparison of associations of overweight-years at Visit 2 and BMI at Visit 2 with cancer by Akaike information criterion in the ARIC cohort.

AIC					
**Characteristic**	**MV-adjusted overweight-years**	**MV-adjusted BMI**	**MV-adjusted overweight - years with BMI**	**MV-adjusted degree of overweight**	**MV-adjusted duration of overweight**
**Men**					
All cancers	31897.32	31897.79	31899.25	31897.21	31898.85
OBR-cancers	6281.44	6281.91	6282.03	6280.99	6288.82
NOBR-cancers	25617.02	25616.96	25618.95	25617.02	25616.91
NOBR-cancers excluding lung and prostate	8884.30	8882.97	8884.33	8884.27	8884.29
**Specific cancer sites**					
Colorectal	2682.07	2690.34	2683.73	2681.82	2694.30
Kidney	1047.23	1047.18	1049.10	1047.23	1046.24
Bladder	1057.84	1058.08	1059.59	1057.81	1058.92
Pancreas	963.37	962.51	962.57	963.41	963.31
Lung	4723.45	4713.66	4714.27	4723.24	4719.93
Prostate	11892.10	11891.86	11893.85	11892.10	11890.86
Metastatic Prostate	828.18	829.02	829.91	828.24	828.04
**Women**					
All cancers	29086.20	29072.77	29072.89	29086.73	29093.55
OBR-cancers	18079.48	18069.79	18069.63	18080.40	18086.57
NOBR-cancers	10944.31	10944.14	10942.91	10945.45	10944.82
NOBR-cancers excluding lung	7365.58	7362.58	7360.83	7366.94	7367.22
**Specific cancer sites**					
Colorectal	2894.98	2893.84	2894.07	2896.14	2895.26
Pancreas	861.80	868.32	863.02	863.51	866.64
Kidney	917.93	917.37	917.42	919.64	921.31
Lung	3517.46	3518.52	3519.21	3518.68	3516.88
Endometrial	1771.70	1754.04	1754.72	1770.73	1781.54
Ovarian	1055.50	1057.08	1057.46	1056.81	1056.37
Post-menopausal breast cancer	8843.97	8842.84	8843.55	8845.33	8845.67

* Multivariable adjustment for baseline age, ethnicity, alcohol, smoking and HRT (in women).
Abbreviations: SE, standard error; OBR, obesity-related; NOBR, non-obesity related; BMI, body mass index; AIC, Akaike information criterion; MV, multivariable.

Table S10: Example of (a) apparent and (b) bootstrapped C-statistic performance estimates.

(A)

Characteristic	MV-adjusted overweight-years	MV-adjusted BMI	Difference in c-statistic between BMI and overweight-years (95% CI)	MV-adjusted degree	MV-adjusted duration	Difference in c-statistic between MV-adjusted duration and MV-adjusted degree (95% CI)
All cancers	0.6037 (0.5894,0.6183)	0.6012 (0.5933,0.6092)	-0.0025 (-0.0179,0.0130)	0.6013 (0.5907,0.6122)	0.6022 (0.5737,0.6321)	0.0009 (-0.0101,0.0119)
OBR-cancers	0.5872 (0.566,0.6092)	0.5945 (0.5733,0.6166)	0.0073 (-0.0421,0.0567)	0.5874 (0.562,0.6139)	0.5857 (0.5615,0.611)	-0.0016 (-0.0341,0.0309)
NOBR-cancers	0.6067 (0.5978,0.6158)	0.6054 (0.5965,0.6145)	-0.0013 (-0.0101,0.0075)	0.6068 (0.5979,0.6158)	0.6054 (0.5961,0.6149)	-0.0013 (-0.0102,0.0075)

(B)

Characteristic	MV-adjusted overweight-years	MV-adjusted BMI	Difference in c-statistic between BMI and overweight-years (95% CI)	MV-adjusted degree	MV-adjusted duration	Difference in c-statistic between MV-adjusted duration and MV-adjusted degree (95% CI)
All cancers	0.6025 (0.5918,0.6134)	0.6028 (0.5919,0.614)	0.0004 (-0.0075,0.0082)	0.6032 (0.5914,0.6153)	0.6007 (0.5891,0.6125)	-0.0025 (-0.0115,0.0065)
OBR-cancers	0.5908 (0.5734,0.6087)	0.5912 (0.5740,0.6088)	0.0004 (-0.0111,0.0118)	0.5904 (0.5732,0.6081)	0.5848 (0.5677,0.6025)	-0.0056 (-0.0178,0.0067)
NOBR-cancers	0.6057 (0.5945,0.6171)	0.6066 (0.5953,0.6182)	0.0009 (-0.0089,0.0107)	0.6077 (0.5972,0.6185)	0.6049 (0.5937,0.6163)	-0.0028 (-0.0082,0.0026)

Table S11: Comparison of the overweight-years metric at Visit 2 and BMI at Visit 2 using Harrell's C-statistic, ARIC.

Characteristic	MV-adjusted overweight-years	MV-adjusted BMI	MV-adjusted overweight -years with BMI	Difference in c-statistic between MV-adjusted overweight-years with BMI combined compared with overweight-years (95% CI)	Difference in c-statistic between MV-adjusted overweight-years with BMI combined and MV-adjusted BMI (95% CI)
All cancers	0.603 (0.592,0.613)	0.603 (0.592,0.614)	0.600 (0.590,0.612)	-0.001 (-0.008,0.006)	-0.001 (-0.008,0.005)
OBR-cancers	0.591 (0.573,0.609)	0.591 (0.57,0.609)	0.590 (0.572,0.608)	0.002 (-0.007,0.010)	0.001 (-0.006,0.008)
NOBR-cancers	0.606 (0.595,0.617)	0.607 (0.595,0.618)	0.607 (0.583,0.633)	0.001 (-0.009,0.012)	0.001 (-0.009,0.010)
NOBR-cancers excluding lung and prostate	0.590 (0.576,0.605)	0.590 (0.576,0.605)	0.591 (0.576,0.606)	0.000 (-0.005,0.006)	0.001 (-0.003,0.004)
	Specific Cancer Sites				
Colorectal	0.658 (0.632,0.686)	0.641 (0.615,0.669)	0.658 (0.632,0.685)	-0.001 (-0.015,0.012)	0.016 (-0.003,0.034)
Pancreas	0.542 (0.499,0.589)	0.551 (0.506,0.599)	0.582 (0.536,0.632)	0.040 (0.002,0.078)	0.031 (0.008,0.054)
Kidney	0.601 (0.551,0.655)	0.603 (0.554,0.656)	0.611 (0.561,0.664)	0.009 (-0.020,0.039)	0.008 (-0.008,0.023)
Lung	0.723 (0.705, 0.741)	0.731 (0.713,0.750)	0.733 (0.714,0.752)	0.010 (-0.001,0.021)	0.002 (-0.002,0.005)
Prostate	0.606 (0.593,0.619)	0.605 (0.592,0.618)	0.604 (0.591,0.618)	-0.001 (-0.005,0.003)	0.000 (-0.002,0.002)
Metastatic prostate	0.596 (0.544,0.654)	0.587 (0.538,0.640)	0.603 (0.550,0.662)	0.007 (-0.008,0.022)	0.017 (-0.025,0.058)
Bladder	0.677 (0.635,0.721)	0.678 (0.636,0.723)	0.680 (0.638,0.724)	0.003 (-0.007,0.012)	0.002 (-0.015,0.018)
	Women				
All cancers	0.579 (0.568,0.590)	0.584 (0.573,0.594)	0.584 (0.541,0.630)	0.005 (-0.004,0.015)	0.000 (-0.007,0.007)
OBR-cancers	0.562 (0.551,0.573)	0.575 (0.564,0.586)	0.573 (0.557,0.590)	0.012 (0.001,0.024)	-0.001 (-0.008,0.006)
NOBR-cancers	0.636 (0.622,0.651)	0.636 (0.622,0.651)	0.638 (0.623,0.652)	0.000 (-0.010,0.011)	0.000 (-0.016,0.017)
NOBR-cancers excluding lung	0.592 (0.576,0.609)	0.598 (0.582,0.615)	0.603 (0.586,0.620)	0.009 (-0.004,0.022)	0.003 (-0.006,0.012)
	Specific Cancer Sites				
Colorectal	0.575 (0.549,0.601)	0.586 (0.561,0.612)	0.582 (0.557,0.609)	0.008 (-0.023,0.038)	-0.004 (-0.019,0.012)
Pancreas	0.653 (0.600,0.710)	0.637 (0.584,0.694)	0.658 (0.608,0.713)	0.005 (-0.015,0.025)	0.0212 (-0.0212,0.064)
Kidney	0.652 (0.597,0.711)	0.663 (0.611,0.720)	0.663 (0.611,0.720)	0.012 (-0.020,0.043)	0.000 (-0.006,0.007)
Lung	0.754 (0.733,0.775)	0.753 (0.733,0.775)	0.754 (0.733,0.775)	-0.001 (-0.004,0.002)	-0.000 (-0.002,0.002)
Endometrial	0.647 (0.612,0.685)	0.669 (0.631,0.708)	0.669 (0.631,0.708)	0.0211 (-0.015,0.057)	0.000 (-0.000,0.000)
Ovarian	0.575 (0.534,0.620)	0.563 (0.52,0.606)	0.574 (0.533,0.618)	-0.001 (-0.008,0.006)	0.011 (-0.018,0.400)
Post-menopausal breast cancer	0.581 (0.566,0.596)	0.590 (0.576,0.605)	0.589 (0.574,0.604)	0.009 (-0.004,0.022)	-0.000 (-0.005,0.004)

Abbreviations: SE, standard error; OBR, obesity-related; NOBR, non-obesity related; BMI, body mass index; CI, confidence interval; MV, multivariable-adjusted.
Key: Green – significant difference in C-statistic.

a) Analysis of obese-years exposure

Table S12: Incidence of cancer (events/1000 Person-Years) according to obese-years at Visit 2 and BMI at Visit 2 by gender, ethnicity, smoking, HRT (women only) in the ARIC cohort.

	0 obese-years (kg-years/m²)			>0-100 obese-years (kg-years/m²)			>100 obese-years (kg-years/m²)			BMI (kg/m²)		
	**N**	**PYFU**	**IR (95% CI)**	**N**	**PYFU**	**IR (95% CI)**	**N**	**PYFU**	**IR (95% CI)**	**N**	**PYFU**	**IR (95% CI)**
**Men**												
**Whole sample**	1486	185441.50	8.01 (7.60, 8.42)	559	65531.38	8.53 (7.82, 9.25)	27	3568.35	7.57 (4.56, 10.58)	2072	254541.30	8.14 (7.79, 8.49)
**Ethnicity**												
**White**	1176	152792.40	7.70 (7.25, 8.14)	413	51034.90	8.09 (7.30, 8.88)	18	2730.68	6.59 (3.34, 9.85)	1607	206558.00	7.78 (7.40, 8.16)
**Black**	310	32649.12	9.49 (8.42, 10.57)	146	14496.48	10.07 (8.4, 11.74)	9	837.67	10.74 (3.00, 18.49)	465	47983.27	9.69 (8.80, 10.58)
**Smoking**												
**Ever**	1135	135153.30	8.40 (7.90, 8.89)	416	47177.03	8.82 (7.96, 9.68)	15	2170.44	6.91 (3.15, 10.68)	1566	184500.80	8.49 (8.06, 8.91)
**Never**	351	50288.23	6.99 (6.24, 7.73)	143	18354.34	7.79 (6.49, 9.10)	12	1397.91	8.59 (3.30, 13.87)	506	70040.48	7.23 (6.59, 7.87)
**Women**												
**Whole sample**	1009	207699.10	4.86 (4.66, 14.38)	625	123055.10	5.08 (4.68, 5.48)	170	27638.49	6.15 (5.21, 7.09)	1804	358392.70	5.03 (4.80, 5.27)
**Ethnicity**												
**White**	818	166274.50	4.92 (4.58, 5.26)	420	81206.63	5.17 (4.67, 5.67)	98	14430.61	6.79 (5.41, 8.17)	1336	261911.80	5.10 (4.83, 5.38)
**Black**	191	41424.53	4.61 (3.94, 5.28)	205	41848.49	4.90 (4.22, 5.58)	72	13207.88	5.45 (4.15, 6.75)	468	96480.90	4.85 (4.41, 5.30)
**Smoking**												
**Ever**	596	106474.60	5.60 (5.14, 6.05)	310	56253.63	5.51 (4.88, 6.13)	78	11221.82	6.95 (5.36, 8.54)	984	173950.00	5.66 (5.30, 6.01)
**Never**	413	101224.50	4.08 (3.68, 4.48)	315	66801.48	4.72 (4.19, 5.25)	92	16416.67	5.60 (4.43, 6.78)	820	184442.60	4.45 (4.14, 4.75)
**HRT**												
**Ever**	421	84467.88	4.99 (4.50, 5.47)	180	39102.38	4.60 (3.92, 5.29)	27	5816.41	4.66 (2.81, 6.51)	628	129386.70	4.85 (4.47, 5.24)
**Never**	588	123231.20	4.77 (4.38, 5.16)	445	83952.73	5.30 (4.80, 5.80)	143	21822.08	6.55 (5.45, 7.65)	1176	229006.00	5.13 (4.84, 5.43)

Abbreviations: N, number of events; PYFR, person-years of follow-up; IR, incidence rate of all cancers; BMI, body mass index; CI, confidence interval.

Table S13: Hazard ratio of cancers by obese-years at Visit 2 and BMI at Visit 2 in ARIC.

Outcome	**Obese-years, (kg-years/m^2) (per SD)**		**BMI (kg/m^2) (per SD)**	
	**Age-adjusted HR (95% CI)**	**MV-adjusted HR (95% CI)**	**Age-adjusted HR (95% CI)**	**MV-adjusted HR (95% CI)**
	**Men**			
All Cancers	1.08 (1.04,1.13)	1.08 (1.03,1.13)	1.03 (0.98,1.08)	1.03 (0.98,1.08)
OBR-cancers	1.15 (1.06,1.26)	1.15 (1.05,1.25)	1.14 (1.02,1.27)	1.14 (1.02,1.27)
NOBR-cancers	1.06 (1.01,1.12)	1.06 (1.01,1.12)	1.00 (0.95,1.06)	1.01 (0.95,1.07)
NOBR-cancers excluding lung and prostate	1.12 (1.03,1.21)	1.13 (1.04,1.22)	1.12 (1.02,1.23)	1.12 (1.02,1.23)
**Specific cancer sites**				
Colorectal	1.24 (1.12,1.38)	1.23 (1.11,1.37)	1.27 (1.08,1.49)	1.27 (1.08,1.49)
Kidney	1.01 (0.76,1.34)	1.01 (0.76,1.34)	0.97 (0.74,1.27)	0.97 (0.74,1.28)
Bladder	1.06 (0.82,1.38)	1.06 (0.82,1.39)	1.18 (0.90,1.53)	1.16 (0.89,1.51)
Pancreas	1.02 (0.75,1.39)	1.01 (0.74,1.38)	1.17 (0.88,1.55)	1.16 (0.87,1.54)
Lung	1.00 (0.87,1.15)	1.00 (0.87,1.15)	0.76 (0.66,0.89)	0.77 (0.66,0.89)
Prostate	1.04 (0.96,1.13)	1.03 (0.95,1.11)	1.01 (0.93,1.10)	1.01 (0.93,1.10)
Metastatic Prostate	0.95 (0.64,1.40)	0.93 (0.63,1.37)	0.95 (0.67,1.35)	0.96 (0.68,1.35)
**Women**				
All Cancers	1.07 (1.02,1.11)	1.08 (1.03,1.13)	1.11 (1.06,1.16)	1.14 (1.08,1.19)
OBR-cancers	1.11 (1.06,1.17)	1.11 (1.05,1.17)	1.18 (1.12,1.25)	1.19 (1.12,1.26)
NOBR-cancers	0.99 (0.91,1.07)	1.02 (0.94,1.10)	0.99 (0.91,1.07)	1.05 (0.97,1.14)
NOBR-cancers excluding lung	1.03 (0.94,1.12)	1.05 (0.96,1.15)	1.06 (0.97,1.17)	1.12 (1.01,1.23)
**Specific cancer sites**				
Colorectal	1.05 (0.91,1.20)	1.02 (0.88,1.17)	1.19 (1.03,1.36)	1.14 (0.98,1.32)
Pancreas	1.28 (1.08,1.52)	1.22 (1.03,1.46)	1.29 (1.01,1.64)	1.16 (0.89,1.52)
Kidney	1.28 (1.08,1.51)	1.24 (1.05,1.48)	1.45 (1.17,1.81)	1.40 (1.10,1.78)
Lung	0.89 (0.76,1.04)	0.94 (0.81,1.11)	0.83 (0.72,0.97)	0.92 (0.79,1.07)
Endometrial	1.31 (1.16,1.47)	1.36 (1.20,1.54)	1.60 (1.38,1.86)	1.75 (1.49,2.06)
Ovarian	1.07 (0.86,1.35)	1.12 (0.89,1.41)	1.01 (0.79,1.30)	1.10 (0.85,1.42)
Post-menopausal breast cancer	1.04 (0.96,1.13)	1.05 (0.97,1.14)	1.09 (1.00,1.18)	1.11 (1.02,1.21)

* Multivariable adjustment for baseline age, ethnicity, alcohol, smoking and HRT (in women).
Abbreviations: OBR, obesity-related; NOBR, non-obesity related; CI, confidence interval; HR, hazard ratio; BMI, body mass index; MV, multivariable; SD, standard deviation.

Table S14: Hazard ratio of cancers by obese-years per 100 units at Visit 2 and BMI per 5 units (kg/m^2) at Visit 2 in ARIC.

Outcomes	Obese-years (per 100 kg-years/m^2)		BMI (per 5 unit [kg/m^2])	
	Age-adjusted HR (95% CI)	MV-adjusted HR (95% CI)	Age-adjusted HR (95% CI)	MV-adjusted HR (95% CI)
	**Men**			
All Cancers	1.16 (0.99,1.37)	1.16 (0.98,1.36)	1.03 (0.97,1.08)	1.03 (0.98,1.08)
OBR-cancers	1.70 (1.29,2.24)	1.68 (1.28,2.22)	1.19 (1.06,1.33)	1.18 (1.06,1.33)
NOBR-cancers	1.01 (0.83,1.23)	1.01 (0.82,1.23)	0.99 (0.93,1.05)	0.99 (0.93,1.05)
NOBR-cancers excluding lung and prostate	1.07 (0.77,1.49)	1.10 (0.79,1.52)	1.05 (0.95,1.17)	1.06 (0.96,1.18)
**Specific cancer sites**				
Colorectal	2.20 (1.58,3.06)	2.19 (1.57,3.06)	1.35 (1.15,1.59)	1.34 (1.14,1.58)
Kidney	1.30 (0.55,3.06)	1.26 (0.54,2.94)	1.04 (0.77,1.39)	1.03 (0.77,1.39)
Bladder	1.37 (0.62,3.06)	1.49 (0.65,3.41)	1.20 (0.91,1.59)	1.21 (0.92,1.60)
Pancreas	0.62 (0.15,2.53)	0.61 (0.15,2.51)	1.16 (0.87,1.55)	1.16 (0.87,1.55)
Lung	0.78 (0.46,1.33)	0.82 (0.47,1.42)	0.76 (0.65,0.88)	0.78 (0.67,0.90)
Prostate	1.06 (0.80,1.40)	1.01 (0.76,1.34)	1.04 (0.95,1.13)	1.03 (0.95,1.12)
Metastatic Prostate	1.52 (0.67,3.48)	1.46 (0.63,3.37)	1.05 (0.76,1.44)	1.04 (0.76,1.43)
	**Women**			
All Cancers	1.13 (1.05,1.22)	1.15 (1.06,1.25)	1.09 (1.05,1.13)	1.11 (1.07,1.16)
OBR-cancers	1.22 (1.11,1.34)	1.21 (1.10,1.33)	1.14 (1.09,1.20)	1.15 (1.10,1.21)
NOBR-cancers	0.97 (0.84,1.13)	1.03 (0.89,1.20)	0.99 (0.93,1.05)	1.04 (0.97,1.11)
NOBR-cancers excluding lung	1.05 (0.89,1.24)	1.09 (0.92,1.29)	1.05 (0.98,1.14)	1.09 (1.01,1.19)
**Specific cancer sites**				
Colorectal	1.09 (0.85,1.41)	1.03 (0.79,1.34)	1.15 (1.03,1.29)	1.11 (0.98,1.26)
Pancreas	1.59 (1.16,2.16)	1.46 (1.05,2.02)	1.23 (1.01,1.50)	1.13 (0.91,1.41)
Kidney	1.58 (1.16,2.15)	1.50 (1.09,2.07)	1.36 (1.14,1.63)	1.32 (1.09,1.6)
Lung	0.8 (0.60,1.08)	0.90 (0.67,1.21)	0.86 (0.76,0.97)	0.93 (0.82,1.06)
Endometrial	1.65 (1.33,2.05)	1.76 (1.40,2.22)	1.47 (1.30,1.66)	1.59 (1.39,1.81)
Ovarian	1.08 (0.93,1.26)	1.10 (0.94,1.27)	1.07 (1.00,1.14)	1.09 (1.01,1.17)
Post-menopausal breast cancer	1.09 (0.85,1.41)	1.03 (0.79,1.34)	1.15 (1.03,1.29)	1.11 (0.98,1.26)

* Multivariable adjustment for baseline age, ethnicity, alcohol, smoking and HRT (in women).
Abbreviations: OBR, obesity-related; NOBR, non-obesity related; CI, confidence interval; HR, hazard ratio; BMI, body mass index; MV, multivariable.

Table S15: Hazard ratio of cancers by obese degree and duration at Visit 2, ARIC.

Outcome	Degree of Obesity, kg/m^2 (per SD)		Duration of Obesity, years (per SD)	
	Age-adjusted HR (95% CI)	MV-adjusted HR (95% CI)	Age-adjusted HR (95% CI)	MV-adjusted HR (95% CI)
	**Men**			
All Cancers	1.04 (0.99,1.10)	1.04 (0.99,1.09)	1.06 (1.01,1.11)	1.05 (1.00,1.11)
OBR-cancers	1.12 (1.02,1.22)	1.11 (1.01,1.22)	1.11 (1.00,1.23)	1.11 (0.99,1.23)
NOBR-cancers	1.01 (0.96,1.08)	1.01 (0.95,1.07)	1.04 (0.98,1.11)	1.04 (0.98,1.10)
NOBR-cancers excluding lung and prostate	0.99 (0.89,1.10)	0.99 (0.9,1.10)	1.01 (0.91,1.11)	1.01 (0.91,1.12)
**Specific cancer sites**				
Colorectal	1.17 (1.04,1.31)	1.17 (1.04,1.31)	1.22 (1.06,1.40)	1.22 (1.06,1.40)
Kidney	1.08 (0.83,1.41)	1.07 (0.83,1.39)	1.12 (0.84,1.48)	1.11 (0.84,1.47)
Bladder	0.98 (0.74,1.31)	1.00 (0.75,1.35)	1.09 (0.84,1.42)	1.10 (0.85,1.43)
Pancreas	0.72 (0.44,1.17)	0.71 (0.44,1.16)	0.85 (0.58,1.23)	0.84 (0.58,1.22)
Lung	1.08 (0.95,1.23)	1.10 (0.95,1.26)	1.15 (1.02,1.30)	1.15 (1.01,1.29)
Prostate	1.00 (0.91,1.09)	0.98 (0.90,1.08)	1.02 (0.94,1.12)	1.01 (0.93,1.10)
Metastatic Prostate	1.13 (0.87,1.47)	1.11 (0.86,1.45)	1.13 (0.83,1.54)	1.11 (0.82,1.52)
	**Women**			
All Cancers	1.00 (0.95,1.07)	1.00 (0.94,1.06)	1.00 (0.94,1.07)	1.00 (0.94,1.06)
OBR-cancers	1.01 (0.93,1.08)	1.00 (0.93,1.08)	1.01 (0.94,1.09)	1.01 (0.94,1.09)
NOBR-cancers	0.99 (0.9,1.10)	0.98 (0.88,1.09)	0.99 (0.9,1.09)	0.99 (0.89,1.09)
NOBR-cancers excluding lung	0.98 (0.86,1.11)	0.97 (0.85,1.10)	0.96 (0.85,1.09)	0.96 (0.85,1.08)
**Specific cancer sites**				
Colorectal	0.88 (0.71,1.08)	0.89 (0.72,1.09)	0.89 (0.73,1.09)	0.9 (0.74,1.10)
Pancreas	1.25 (0.97,1.60)	1.26 (0.99,1.62)	1.40 (1.06,1.86)	1.42 (1.08,1.89)
Kidney	1.06 (0.80,1.41)	1.07 (0.81,1.42)	1.03 (0.75,1.41)	1.04 (0.76,1.44)
Lung	1.00 (0.83,1.22)	1.00 (0.82,1.21)	1.06 (0.89,1.25)	1.05 (0.89,1.25)
Endometrial	0.95 (0.76,1.19)	0.95 (0.75,1.19)	0.98 (0.77,1.26)	0.96 (0.74,1.23)
Ovarian	1.12 (0.83,1.52)	1.11 (0.81,1.51)	0.88 (0.62,1.27)	0.87 (0.60,1.25)
Post-menopausal breast cancer	0.98 (0.87,1.10)	0.97 (0.87,1.09)	1.03 (0.93,1.15)	1.03 (0.92,1.15)

* Multivariable adjustment for baseline age, ethnicity, alcohol, smoking and HRT (in women).
* Degree of obesity is the cumulative sum of the number of BMI units ≥ 30 kg/m^2 over the exposure period.
* Duration of obesity is the cumulative sum of the duration overweight (BMI ≥ 30 kg/m^2) over the exposure period.
Abbreviations: OBR, obesity-related; NOBR, non-obesity related; CI, confidence interval; HR, hazard ratio; BMI, body mass index; MV, multivariable; SD, standard deviation.

Table S16: Hazard ratio of cancers by obesity degree and duration per 10 units and per 10 years, respectively at Visit 2, ARIC.

Outcome	Degree of Obesity per 10 kg/m^2)		Duration of Obesity (per 10 years)	
	Age-adjusted HR (95% CI)	MV-adjusted HR (95% CI)	Age-adjusted HR (95% CI)	MV-adjusted HR (95% CI)
	**Men**			
All Cancers	1.02 (1.00,1.03)	1.01 (1.00,1.03)	1.09 (1.01,1.17)	1.08 (1.00,1.17)
OBR-cancers	1.04 (1.01,1.08)	1.04 (1.01,1.08)	1.16 (0.99,1.36)	1.16 (0.99,1.36)
NOBR-cancers	1.01 (0.98,1.03)	1.00 (0.98,1.03)	1.07 (0.98,1.16)	1.06 (0.97,1.16)
NOBR-cancers excluding lung and prostate	1.00 (0.96,1.04)	1.00 (0.96,1.04)	1.01 (0.87,1.17)	1.02 (0.88,1.19)
**Specific cancer sites**				
Colorectal	1.06 (1.01,1.11)	1.06 (1.01,1.11)	1.34 (1.09,1.65)	1.34 (1.09,1.64)
Kidney	1.03 (0.93,1.14)	1.03 (0.93,1.13)	1.18 (0.78,1.79)	1.17 (0.77,1.77)
Bladder	0.99 (0.89,1.11)	1.00 (0.90,1.12)	1.14 (0.77,1.69)	1.16 (0.78,1.71)
Pancreas	0.88 (0.74,1.06)	0.88 (0.73,1.06)	0.78 (0.45,1.35)	0.78 (0.45,1.35)
Lung	1.03 (0.98,1.08)	1.03 (0.98,1.09)	1.23 (1.02,1.47)	1.22 (1.02,1.47)
Prostate	1.00 (0.97,1.03)	0.99 (0.96,1.03)	1.04 (0.91,1.18)	1.02 (0.89,1.16)
Metastatic Prostate	1.05 (0.95,1.15)	1.04 (0.94,1.15)	1.20 (0.76,1.89)	1.17 (0.74,1.85)
**Women**				
All Cancers	1.01 (1.00,1.02)	1.01 (1.01,1.02)	1.07 (1.02,1.12)	1.08 (1.03,1.13)
OBR-cancers	1.00 (0.98,1.01)	1.00 (0.99,1.02)	0.99 (0.92,1.06)	1.02 (0.95,1.10)
NOBR-cancers	1.02 (1.01,1.03)	1.02 (1.01,1.03)	1.12 (1.06,1.19)	1.12 (1.06,1.18)
NOBR-cancers excluding lung	1.00 (0.94,1.07)	1.00 (0.94,1.08)	0.80 (0.52,1.24)	0.80 (0.51,1.25)
**Specific cancer sites**				
Colorectal	1.01 (0.99,1.03)	1.00 (0.98,1.03)	1.05 (0.92,1.21)	1.02 (0.89,1.18)
Pancreas	1.05 (1.03,1.07)	1.06 (1.04,1.08)	1.39 (1.18,1.64)	1.43 (1.21,1.68)
Kidney	1.05 (1.02,1.08)	1.04 (1.01,1.07)	1.3 (1.04,1.62)	1.26 (1.01,1.58)
Lung	0.98 (0.95,1.01)	0.99 (0.96,1.02)	0.93 (0.81,1.06)	0.98 (0.86,1.13)
Endometrial	1.01 (0.99,1.02)	1.01 (0.99,1.03)	1.02 (0.93,1.11)	1.04 (0.95,1.14)
Ovarian	1.01 (0.97,1.05)	1.02 (0.98,1.06)	0.94 (0.73,1.23)	0.98 (0.75,1.28)
Post-menopausal breast cancer	1.05 (1.01,1.08)	1.04 (1.00,1.07)	1.40 (1.14,1.74)	1.34 (1.08,1.67)

* Multivariable adjustment for baseline age, ethnicity, alcohol, smoking and HRT (in women).
* Degree of obese is the cumulative sum of the number of BMI units ≥ 30 kg/m^2 over the exposure period.
* Duration of obese is the cumulative sum of the duration overweight (BMI ≥ 30 kg/m^2) over the exposure period.

Abbreviations: OBR, obesity-related; NOBR, non-obesity related; CI, confidence interval; HR, hazard ratio; BMI, body mass index; MV, multivariable.

Table S17: Comparison of associations of obese-years at Visit 2 and BMI at Visit 2 with cancer by Akaike information criterion in the ARIC cohort.

AIC					
**Characteristic**	**MV-adjusted obese-years**	**MV-adjusted BMI**	**MV-adjusted obese -years with BMI**	**MV-adjusted degree of obesity**	**MV-adjusted duration of obesity**
**Men**					
All cancers	31896.05	31897.79	31898.03	31895.70	31893.93
OBR-cancers	6279.58	6281.91	6279.91	6278.71	6280.17
NOBR-cancers	25617.02	25616.96	25618.89	25617.02	25616.29
NOBR-cancers excluding lung and prostate	8884.06	8882.97	8884.95	8884.00	8883.48
**Specific cancer sites**					
Colorectal	2687.33	2690.34	2687.10	2686.81	2684.83
Kidney	1046.97	1047.18	1048.97	1046.94	1046.76
Bladder	1059.09	1058.08	1060.06	1059.20	1057.97
Pancreas	962.88	962.51	961.69	963.06	963.44
Lung	4724.65	4713.66	4714.52	4724.63	4725.14
Prostate	11892.28	11891.86	11893.73	11892.28	11891.93
Metastatic prostate	828.43	829.02	830.38	828.52	828.63
**Women**					
All cancers	29088.34	29072.77	29074.77	29087.52	29087.80
OBR-cancers	18086.59	18069.79	18071.77	18085.79	18085.72
NOBR-cancers	10945.26	10944.14	10946.02	10945.21	10945.18
NOBR-cancers excluding lung	7366.33	7362.58	7364.32	7366.20	7366.58
Colorectal	2896.49	2893.84	2894.50	2896.46	2896.45
Pancreas	865.48	868.32	867.39	865.60	863.23
Kidney	919.61	917.37	919.13	919.45	920.62
Lung	3519.19	3518.52	3520.51	3519.20	3519.67
Endometrial	1778.00	1754.04	1755.77	1776.50	1778.87
Ovarian	1056.74	1057.08	1058.72	1056.71	1057.55
Post-menopausal breast cancer	8846.67	8842.84	8844.68	8846.63	8844.48

* Multivariable adjustment for baseline age, ethnicity, alcohol, smoking and HRT (in women).
Abbreviations: SE, standard error; OBR, obesity-related; NOBR, non-obesity related; BMI, body mass index; AIC, Akaike information criterion; MV, multivariable.

Table S18: Comparison of the obese-years metric at Visit 2 and BMI at Visit 2 using Harrell's C-statistic, ARIC

Harrell's C-statistic									
**Characteristic**	**MV-adjusted obese-years**	**MV-adjusted BMI**	**Difference in c-statistic between BMI and obese-years (95% CI)**	**MV-adjusted obese-years with BMI**	**Difference in c-statistic between MV-adjusted obese-years with BMI combined compared with obese-years (95% CI)**	**Difference in c-statistic between MV-adjusted obese-years with BMI combined and MV-adjusted BMI (95% CI)**	**MV-adjusted cumulative obese degree**	**MV-adjusted cumulative obese duration**	**Difference in c-statistic between MV-adjusted duration and MV-adjusted degree (95% CI)**
**Men**									
All cancers	0.604 (0.593, 0.616)	0.599 (0.588, 0.610)	-0.006 (-0.019, 0.008)	0.602 (0.591, 0.613)	-0.002 (-0.010, 0.006)	0.003 (-0.008, 0.014)	0.605 (0.594, 0.616)	0.604 (0.593, 0.616)	-0.000 (-0.011, 0.012)
OBR-cancers	0.593 (0.575, 0.612)	0.591 (0.574, 0.608)	-0.003 (-0.016, 0.011)	0.632 (0.614, 0.651)	0.003 (-0.006, 0.012)	0.005 (-0.003, 0.014)	0.595 (0.577, 0.613)	0.597 (0.579, 0.615)	0.002 (-0.006, 0.010)
NOBR-cancers	0.607 (0.597, 0.618)	0.605 (0.594, 0.617)	-0.002 (-0.013, 0.009)	0.608 (0.546, 0.676)	0.000 (-0.009, 0.009)	0.002 (-0.007, 0.010)	0.606 (0.595, 0.618)	0.606 (0.594, 0.618)	0.000 (-0.007, 0.007)
NOBR-cancers excluding lung and prostate	0.590 (0.576, 0.605)	0.590 (0.576, 0.605)	0.000 (-0.003, 0.004)	0.590 (0.576, 0.605)	0.000 (-0.004, 0.004)	0.000 (-0.001, 0.001)	0.590 (0.576, 0.605)	0.590 (0.576, 0.605)	0.000 (-0.002, 0.002)
**Specific cancer sites**									
Colorectal	0.656 (0.628, 0.685)	0.642 (0.616, 0.669)	-0.014 (-0.037, 0.008)	0.656 (0.629, 0.684)	-0.002 (-0.014, 0.011)	0.0123 (-0.002, 0.027)	0.658 (0.630, 0.688)	0.664 (0.637, 0.693)	0.006 (-0.01, 0.022)
Kidney	0.611 (0.561, 0.666)	0.603 (0.554, 0.656)	-0.008 (-0.024, 0.007)	0.612 (0.562, 0.667)	0.001 (-0.003, 0.005)	0.009 (-0.009, 0.027)	0.612 (0.561, 0.666)	0.604 (0.554, 0.658)	-0.008 (-0.017, 0.001)
Bladder	0.679 (0.638, 0.724)	0.678 (0.636, 0.723)	-0.001 (-0.028, 0.026)	0.680 (0.638, 0.725)	0.000 (-0.024, 0.024)	0.002 (-0.003, 0.006)	0.679 (0.638, 0.723)	0.677 (0.635, 0.721)	-0.003 (-0.013, 0.008)
Pancreas	0.547 (0.503, 0.594)	0.551 (0.506, 0.599)	0.004 (-0.029, 0.037)	0.583 (0.536, 0.634)	0.036 (-0.007, 0.080)	0.032 (0.012, 0.053)	0.545 (0.502, 0.592)	0.538 (0.495, 0.585)	-0.007 (-0.015, 0.001)
Lung	0.722 (0.704, 0.740)	0.731 (0.713, 0.750)	0.010 (-0.001, 0.020)	0.733 (0.714, 0.753)	0.011 (-0.001, 0.023)	0.001 (-0.001, 0.004)	0.722 (0.704, 0.740)	0.721 (0.703, 0.739)	-0.001 (-0.002, 0.001)
Prostate	0.605 (0.592, 0.619)	0.606 (0.593, 0.619)	0.006 (-0.004, 0.005)	0.603 (0.590, 0.617)	-0.001 (-0.006, 0.004)	-0.001 (-0.006, 0.003)	0.605 (0.592, 0.619)	0.606 (0.593, 0.620)	0.001 (-0.004, 0.006)
Metastatic prostate	0.595 (0.546, 0.649)	0.587 (0.538, 0.640)	-0.008 (-0.035, 0.018)	0.599 (0.549, 0.653)	0.004 (-0.008, 0.015)	0.012 (-0.021, 0.045)	0.594 (0.544, 0.647)	0.594 (0.544, 0.649)	0.000 (-0.016, 0.016)
**Women**									
All cancers	0.578 (0.567, 0.588)	0.583 (0.573, 0.594)	0.006 (-0.006, 0.017)	0.586 (0.541, 0.634)	0.008 (-0.005, 0.020)	0.002 (-0.006, 0.010)	0.578 (0.567, 0.588)	0.578 (0.568, 0.589)	0.001 (-0.008, 0.009)
OBR-cancers	0.552 (0.542, 0.563)	0.573 (0.563, 0.584)	0.021 (0.007, 0.035)	0.675 (0.658, 0.692)	0.021 (0.005, 0.037)	-0.000 (-0.007, 0.006)	0.552 (0.541, 0.562)	0.555 (0.545, 0.566)	0.003 (-0.007, 0.014)
NOBR-cancers	0.634 (0.620, 0.649)	0.635 (0.621, 0.650)	0.001 (-0.008, 0.010)	0.638 (0.623, 0.653)	0.002 (-0.007, 0.018)	0.001 (-0.003, 0.006)	0.635 (0.621, 0.650)	0.635 (0.621, 0.649)	-0.000 (-0.008, 0.007)
NOBR-cancers excluding lung	0.593 (0.576, 0.610)	0.598 (0.582, 0.615)	0.005 (-0.004, 0.015)	0.595 (0.578, 0.613)	0.007 (-0.004, 0.018)	0.002 (-0.004, 0.008)	0.592 (0.576, 0.610)	0.594 (0.577, 0.610)	0.001 (-0.012, 0.014)
**Specific cancer sites**									
Colorectal	0.568 (0.542, 0.595)	0.586 (0.561, 0.612)	0.018 (-0.008, 0.045)	0.585 (0.559, 0.613)	0.017 (-0.018, 0.052)	-0.001 (-0.020, 0.018)	0.569 (0.543, 0.595)	0.569 (0.544, 0.596)	0.001 (-0.003, 0.005)

Pancreas	0.647 (0.594, 0.705)	0.637 (0.584, 0.694)	-0.010 (-0.032, 0.011)	0.648 (0.595, 0.705)	0.001 (-0.007, 0.008)	0.011 (-0.015, 0.037)	0.648 (0.594, 0.706)	0.654 (0.601, 0.710)	0.006 (-0.018, 0.030)
Kidney	0.652 (0.595, 0.715)	0.663 (0.611, 0.720)	0.011 (-0.033, 0.055)	0.664 (0.611, 0.722)	0.012 (-0.024, 0.048)	0.001 (-0.009, 0.011)	0.653 (0.596, 0.716)	0.641 (0.585, 0.702)	-0.013 (-0.032, 0.006)
Lung	0.754 (0.734, 0.775)	0.753 (0.732, 0.775)	-0.001 (-0.005, 0.003)	0.754 (0.733, 0.776)	-0.001 (-0.005, 0.003)	0.000 (-0.001, 0.002)	0.754 (0.733, 0.775)	0.753 (0.733, 0.774)	-0.000 (-0.003, 0.002)
Endometrial	0.637 (0.600, 0.677)	0.669 (0.631, 0.709)	0.031 (-0.013, 0.075)	0.668 (0.630, 0.707)	0.031 (-0.016, 0.077)	-0.001 (-0.005, 0.003)	0.639 (0.602, 0.679)	0.624 (0.588, 0.662)	-0.015 (-0.030, -0.001)
Ovarian	0.568 (0.526, 0.613)	0.563 (0.523, 0.606)	-0.005 (-0.037, 0.027)	0.569 (0.528, 0.613)	0.001 (-0.009, 0.011)	0.006 (-0.019, 0.031)	0.567 (0.526, 0.612)	0.559 (0.519, 0.602)	-0.008 (-0.042, 0.0253)
Post-menopausal breast cancer	0.577 (0.562, 0.592)	0.590 (0.575, 0.605)	0.013 (-0.004, 0.030)	0.589 (0.574, 0.604)	0.013 (-0.005, 0.0316)	0.000 (-0.003, 0.003)	0.577 (0.563, 0.592)	0.583 (0.568, 0.598)	0.006 (-0.006, 0.017)

* Multivariable adjustment for baseline age, ethnicity, alcohol, smoking and HRT (in women).

Abbreviations: SE, standard error; OBR, obesity-related; NOBR, non-obesity related; BMI, body mass index; AIC, Akaike information criterion.

Key: Green – significant difference in C-statistic.

Analysis using observed BMI from the at least 3 observed BMI readings subgroup

a) **Analysis of overweight-years exposure**

Table S19: Summary of the exposure metrics.

Characteristic	Men
Baseline BMI, (kg/m^2)	27.70 (4.30)
End of cancer follow up, (years)	18.00 (8.00)
Total cumulative overweight years, ($kg\text{-}years/m^2$)	34.00 (69.00)
Total cumulative overweight degree, (kg/m^2)	7.00 (7.00)
Total cumulative overweight duration, (years)	24.00 (15.00)
Total cumulative obese-years, ($kg\text{-}years/m^2$)	8.00 (35.00)
Total cumulative obese-degree, (kg/m^2)	1.80 (4.40)
Total cumulative obese-duration, (years)	8.00 (14.00)
	**Women**
Baseline BMI, (kg/m^2)	28.20 (6.10)
End of cancer follow up, (years)	20.00 (7.00)
Total cumulative overweight years, ($kg\text{-}years/m^2$)	77.00 (118.00)
Total cumulative overweight degree, (kg/m^2)	10.00 (11.00)
Total cumulative overweight duration, (years)	20.00 (16.00)
Total cumulative obese-years, ($kg\text{-}years/m^2$)	27.00 (71.00)
Total cumulative obese-degree, (kg/m^2)	4.10 (7.50)
Total cumulative obese-duration, (years)	9.00 (15.00)

Table S20: Incidence of cancer (events/1000 Person-Years) according to overweight years at Visit 2 and BMI at Visit 2 stratified by gender, ethnicity, smoking, HRT (women only) in the ARIC cohort.

	**0 overweight-years (kg-years/m²)**			**>0-100 overweight-years (kg-years/m²)**			**>100 overweight-years (kg-years/m²)**			**Baseline BMI (kg/m²)**		
	**N**	**PYFU**	**IR (95% CI)**	**N**	**PYFU**	**IR (95% CI)**	**N**	**PYFU**	**IR (95% CI)**	**N**	**PYFU**	**IR (95% CI)**
	**Men**											
**Whole sample**	449	89813.17	5 (4.53, 5.47)	1414	287464.3	4.92 (4.66, 5.18)	209	40409.81	5.17 (4.46, 5.89)	2072	417687.3	4.96 (4.75, 5.18)
**Ethnicity**												
**White**	344	73151.65	4.7 (4.20, 5.21)	1106	234588.80	4.71 (4.43, 4.99)	157	33049.5	4.75 (3.99, 5.51)	1607	340790	4.72 (4.48, 4.95)
**Black**	105	16661.52	6.30 (5.06, 7.54)	308	52875.45	5.83 (5.16, 6.49)	52	7360.301	7.06 (5.07, 9.06)	465	76897.27	6.05 (5.49, 6.6)
**Smoking**												
**Ever**	351	65813.76	5.33 (4.77, 5.90)	1062	206757.90	5.14 (4.82, 5.45)	153	28643.96	5.34 (4.48, 6.21)	1565.7	301215.6	5.2 (4.94, 5.46)
**Never**	98	23999.40	4.09 (3.26, 4.92)	352.2	80706.43	4.36 (3.90, 4.83)	56	11765.84	4.76 (3.47, 6.05)	506.3	116471.7	4.35 (3.96, 4.73)
	**Women**											
**Whole sample**	395	140287.30	2.82 (2.53, 3.10)	950	306506.10	0.10 (-0.10, 0.30)	459	161009.4	2.85 (2.59, 3.11)	1804	607802.7	2.97 (2.83, 3.11)
**Ethnicity**												
**White**	344	123367.60	2.79 (2.49, 3.09)	640	199581.70	0.13 (-0.12, 0.38)	352	122488.5	2.87 (2.57, 3.18)	1336	445437.8	3 (2.84, 3.16)
**Black**	51	16919.65	3.01 (2.15, 3.87)	310	106924.40	0.17 (-0.16, 0.49)	107	38520.9	2.78 (2.24, 3.32)	468	162364.9	2.88 (2.62, 3.15)
**Smoking**												
**Ever**	252	75348.88	3.34 (2.92, 3.76)	491	141890.00	0.16 (-0.15, 0.47)	241	75640.71	3.18 (2.77, 3.59)	983.8	292879.6	3.36 (3.15, 3.57)
**Never**	143	64938.38	2.20 (1.83, 2.57)	459	164616.00	0.13 (-0.13, 0.39)	218	85368.64	2.56 (2.21, 2.9)	820.2	314923	2.6 (2.42, 2.78)
**HRT**												
**Ever**	173	62376.59	2.78 (2.36, 3.2)	318	103018.90	0.18 (-0.17, 0.52)	137	55829.58	2.45 (2.03, 2.87)	628.1	221225.1	2.84 (2.61, 3.06)
**Never**	222	77910.68	2.85 (2.46, 3.23)	632	203487.20	0.12 (-0.12, 0.37)	322	105179.8	3.06 (2.72, 3.4)	1175.9	386577.6	3.04 (2.87, 3.22)

Abbreviations: N, number of events; PYFR, person-years of follow-up; IR, incidence rate of all cancers; HRT, hormone replacement therapy.

Table S21: Hazard ratio of cancers per standard deviation of overweight-years at Visit 2 and BMI at Visit 2, ARIC.

Outcome	Overweight-years, (kg-years/m^2) (per SD)		BMI (kg/m^2) (per SD)	
	Age-adjusted HR (95% CI)	MV-adjusted HR (95% CI)	Age-adjusted HR (95% CI)	MV-adjusted HR (95% CI)
	**Men**			
All Cancers	1.01 (0.97,1.05)	1.01 (0.97,1.05)	1.02 (0.98,1.07)	1.02 (0.98,1.07)
OBR-cancers	1.07 (0.98,1.16)	1.07 (0.98,1.15)	1.16 (1.05,1.27)	1.15 (1.05,1.27)
NOBR-cancers	0.99 (0.94,1.04)	0.99 (0.95,1.04)	0.99 (0.94,1.04)	0.99 (0.95,1.04)
NOBR-cancers excluding lung and prostate	0.94 (0.86,1.03)	0.95 (0.87,1.04)	1.05 (0.96,1.14)	1.05 (0.97,1.14)
**Specific cancer sites**				
Colorectal	1.19 (1.08,1.30)	1.18 (1.08,1.29)	1.28 (1.12,1.47)	1.28 (1.12,1.47)
Kidney	0.99 (0.77,1.26)	0.98 (0.77,1.25)	1.03 (0.81,1.32)	1.03 (0.81,1.32)
Bladder	1.12 (0.94,1.33)	1.13 (0.95,1.35)	1.17 (0.93,1.47)	1.17 (0.93,1.48)
Pancreas	0.84 (0.60,1.17)	0.84 (0.61,1.17)	1.13 (0.89,1.45)	1.13 (0.89,1.45)
Lung	1.01 (0.92,1.12)	1.03 (0.93,1.14)	0.80 (0.70,0.90)	0.81 (0.72,0.92)
Prostate	1.01 (0.94,1.08)	1.00 (0.94,1.07)	1.03 (0.96,1.11)	1.02 (0.95,1.10)
Metastatic prostate	1.19 (0.99,1.42)	1.18 (0.99,1.41)	1.04 (0.79,1.36)	1.04 (0.79,1.35)
	**Women**			
All Cancers	0.99 (0.95,1.04)	1.00 (0.95,1.04)	1.11 (1.06,1.16)	1.14 (1.08,1.19)
OBR-cancers	1.04 (0.98,1.10)	1.04 (0.98,1.10)	1.18 (1.11,1.24)	1.19 (1.12,1.26)
NOBR-cancers	0.92 (0.85,1.00)	0.93 (0.86,1.00)	0.99 (0.91,1.07)	1.05 (0.97,1.14)
NOBR-cancers excluding lung	0.92 (0.84,1.01)	0.92 (0.84,1.02)	1.06 (0.97,1.17)	1.12 (1.01,1.23)
**Specific cancer sites**				
Colorectal	0.96 (0.82,1.11)	0.96 (0.83,1.11)	1.19 (1.03,1.36)	1.14 (0.98,1.32)
Pancreas	1.28 (1.07,1.54)	1.28 (1.06,1.54)	1.28 (1.01,1.64)	1.16 (0.89,1.52)
Kidney	1.11 (0.89,1.39)	1.12 (0.89,1.40)	1.45 (1.17,1.81)	1.40 (1.10,1.77)
Lung	0.92 (0.80,1.06)	0.93 (0.81,1.07)	0.83 (0.72,0.96)	0.92 (0.79,1.07)
Endometrial	1.09 (0.92,1.3)	1.09 (0.91,1.30)	1.60 (1.38,1.86)	1.75 (1.49,2.05)
Ovarian	1.08 (0.86,1.36)	1.08 (0.86,1.36)	1.01 (0.79,1.30)	1.10 (0.85,1.42)
Post-menopausal breast cancer	1.00 (0.92,1.09)	1.00 (0.92,1.09)	1.08 (1.00,1.18)	1.11 (1.02,1.21)

* Multivariable adjustment for baseline age, ethnicity, alcohol, smoking and HRT (in women).
Abbreviations: OBR, obesity-related; NOBR, non-obesity related; CI, confidence interval; HR, hazard ratio; BMI, body mass index; MV, multivariable; SD, standard deviation.

Table S22: Hazard ratio of specific cancers per 100 overweight-years and per 5-unit (kg/m^2) BMI in the ARIC cohort.

Outcomes*	Overweight-years (per 100 (kg-years/m^2))		BMI (per 5 unit [kg/m^2])	
	Age-adjusted HR (95% CI)	MV-adjusted HR (95% CI)	Age-adjusted HR (95% CI)	MV-adjusted HR (95% CI)
	Men			
All Cancers	1.01 (0.95,1.07)	1.01 (0.95,1.07)	1.03 (0.97,1.08)	1.03 (0.98,1.08)
OBR-cancers	1.10 (0.98,1.24)	1.10 (0.98,1.24)	1.19 (1.06,1.33)	1.19 (1.06,1.33)
NOBR-cancers	0.98 (0.92,1.05)	0.99 (0.92,1.06)	0.99 (0.93,1.05)	0.99 (0.94,1.05)
NOBR-cancers excluding lung and prostate	0.92 (0.81,1.05)	0.93 (0.81,1.06)	1.05 (0.95,1.17)	1.06 (0.96,1.18)
**Specific cancer sites**				
Colorectal	1.29 (1.13,1.47)	1.28 (1.12,1.47)	1.35 (1.15,1.59)	1.34 (1.14,1.58)
Kidney	0.98 (0.68,1.42)	0.97 (0.67,1.40)	1.04 (0.77,1.39)	1.04 (0.77,1.39)
Bladder	1.18 (0.91,1.52)	1.20 (0.92,1.56)	1.2 (0.91,1.59)	1.21 (0.92,1.60)
Pancreas	0.77 (0.47,1.26)	0.78 (0.48,1.26)	1.16 (0.87,1.56)	1.16 (0.87,1.55)
Lung	1.02 (0.88,1.18)	1.04 (0.90,1.21)	0.76 (0.66,0.88)	0.78 (0.67,0.90)
Prostate	1.01 (0.92,1.12)	1.00 (0.91,1.11)	1.04 (0.95,1.13)	1.03 (0.95,1.12)
Metastatic Prostate	1.29 (0.99,1.68)	1.27 (0.98,1.66)	1.05 (0.76,1.45)	1.04 (0.76,1.43)

Women				
All Cancers	0.99 (0.96,1.03)	1 (0.96,1.04)	1.09 (1.05,1.13)	1.11 (1.07,1.16)
OBR-cancers	1.03 (0.98,1.08)	1.03 (0.98,1.08)	1.14 (1.09,1.20)	1.15 (1.10,1.21)
NOBR-cancers	0.93 (0.87,1.00)	0.94 (0.88,1.00)	0.99 (0.93,1.05)	1.04 (0.97,1.11)
NOBR-cancers excluding lung	0.93 (0.86,1.01)	0.93 (0.86,1.02)	1.05 (0.98,1.14)	1.09 (1.01,1.19)
**Specific cancer sites**				
Colorectal	0.96 (0.85,1.09)	0.96 (0.85,1.10)	1.15 (1.03,1.29)	1.11 (0.98,1.26)
Pancreas	1.24 (1.06,1.44)	1.23 (1.05,1.44)	1.23 (1.01,1.50)	1.13 (0.91,1.41)
Kidney	1.10 (0.91,1.32)	1.10 (0.91,1.33)	1.36 (1.14,1.63)	1.32 (1.09,1.60)
Lung	0.93 (0.83,1.05)	0.94 (0.84,1.06)	0.86 (0.76,0.97)	0.93 (0.82,1.06)
Endometrial	1.08 (0.93,1.25)	1.08 (0.93,1.25)	1.47 (1.30,1.67)	1.59 (1.39,1.81)
Ovarian	1.07 (0.88,1.30)	1.07 (0.88,1.30)	1.01 (0.82,1.24)	1.08 (0.87,1.34)
Post-menopausal breast cancer	1.00 (0.93,1.07)	1.00 (0.93,1.07)	1.07 (1.00,1.14)	1.09 (1.01,1.17)

* Multivariable adjustment for baseline age, ethnicity, alcohol, smoking and HRT (in women).
Abbreviations: OBR, obesity-related; NOBR, non-obesity related; CI, confidence interval; HR, hazard ratio; BMI, body mass index; MV, multivariable.

Table S23: Hazard ratios of cancers per standard deviation overweight degree and duration at Visit 2, in ARIC.

Outcome	Degree of Overweight (kg /m^2) (per SD)		Duration of Overweight (years) (per SD)	
	**Age-adjusted HR (95% CI)**	**MV-adjusted HR (95% CI)**	**Age-adjusted HR (95% CI)**	**MV-adjusted HR (95% CI)**
**Men**				
All Cancers	1.11 (1.01,1.23)	1.09 (0.99,1.21)	0.95 (0.90,1.01)	0.96 (0.90,1.01)
OBR-cancers	1.33 (1.09,1.63)	1.32 (1.08,1.62)	0.86 (0.76,0.97)	0.86 (0.76,0.98)
NOBR-cancers	1.05 (0.94,1.18)	1.03 (0.92,1.16)	0.98 (0.92,1.04)	0.98 (0.92,1.05)
NOBR-cancers excluding lung and prostate	0.95 (0.77,1.16)	0.96 (0.78,1.18)	1.04 (0.93,1.16)	1.04 (0.93,1.15)
**Specific cancer sites**				
Colorectal	1.66 (1.27,2.17)	1.64 (1.25,2.16)	0.83 (0.69,0.99)	0.83 (0.69,1.00)
Kidney	1.02 (0.57,1.81)	1.02 (0.57,1.81)	0.84 (0.61,1.14)	0.84 (0.61,1.14)
Bladder	1.13 (0.65,1.94)	1.15 (0.67,1.98)	1.11 (0.80,1.52)	1.09 (0.79,1.51)
Pancreas	0.87 (0.46,1.66)	0.84 (0.44,1.61)	1.13 (0.80,1.59)	1.14 (0.81,1.61)
Lung	1.35 (1.08,1.70)	1.32 (1.05,1.67)	0.82 (0.72,0.94)	0.82 (0.72,0.94)
Prostate	0.99 (0.83,1.17)	0.95 (0.80,1.13)	1.03 (0.94,1.12)	1.04 (0.95,1.14)
Metastatic Prostate	1.47 (0.88,2.45)	1.41 (0.84,2.38)	0.97 (0.68,1.39)	0.99 (0.69,1.41)
**Women**				
All Cancers	1.00 (0.90,1.10)	0.99 (0.89,1.10)	1.03 (0.97,1.10)	1.05 (0.98,1.11)
OBR-cancers	1.02 (0.90,1.17)	1.02 (0.9,1.16)	1.06 (0.98,1.15)	1.06 (0.98,1.16)
NOBR-cancers	0.94 (0.79,1.11)	0.93 (0.78,1.1)	1.01 (0.91,1.11)	1.03 (0.93,1.14)
NOBR-cancers excluding lung	0.89 (0.72,1.11)	0.89 (0.72,1.1)	1.05 (0.93,1.19)	1.07 (0.94,1.21)
**Specific cancer sites**				
Colorectal	0.84 (0.59,1.18)	0.85 (0.60,1.20)	1.13 (0.92,1.38)	1.10 (0.90,1.35)
Pancreas	1.69 (1.03,2.78)	1.71 (1.05,2.78)	1.05 (0.74,1.51)	1.02 (0.71,1.46)
Kidney	1.08 (0.61,1.90)	1.09 (0.62,1.92)	0.89 (0.63,1.28)	0.88 (0.61,1.25)
Lung	1.00 (0.75,1.33)	0.98 (0.73,1.32)	0.95 (0.79,1.13)	0.98 (0.82,1.17)
Endometrial	1.01 (0.66,1.53)	1.01 (0.66,1.55)	1.03 (0.77,1.36)	1.06 (0.79,1.40)
Ovarian	1.36 (0.83,2.23)	1.35 (0.82,2.23)	1.20 (0.84,1.70)	1.22 (0.86,1.73)
Post-menopausal breast cancer	0.94 (0.77,1.13)	0.94 (0.77,1.13)	1.07 (0.96,1.21)	1.08 (0.96,1.22)

* Multivariable adjustment for baseline age, ethnicity, alcohol, smoking and HRT (in women).
* Degree of overweight is the cumulative sum of the number of BMI units ≥ 25 kg/m^2 over the exposure period.
* Duration of overweight is the cumulative sum of the duration overweight (BMI ≥ 25 kg/m^2) over the exposure period.

Abbreviations: OBR, obesity-related; NOBR, non-obesity related; CI, confidence interval; HR, hazard ratio; BMI, body mass index; MV, multivariable; SD, standard deviation.

Table S24: Hazard ratio of cancers by overweight degree and duration per 10 units and per 10 years, respectively at Visit 2, ARIC.

Outcome	Degree of Overweight (per 10 (kg/m^2))		Duration of Overweight (per 10 years)	
	Age-adjusted HR (95% CI)	MV-adjusted HR (95% CI)	Age-adjusted HR (95% CI)	MV-adjusted HR (95% CI)
**Men**				
All Cancers	1.16 (1.01,1.32)	1.13 (0.99,1.29)	0.97 (0.93,1.00)	0.97 (0.93,1.01)
OBR-cancers	1.48 (1.12,1.94)	1.46 (1.11,1.92)	0.90 (0.83,0.98)	0.91 (0.83,0.98)
NOBR-cancers	1.07 (0.92,1.25)	1.05 (0.90,1.22)	0.99 (0.94,1.03)	0.99 (0.95,1.03)
NOBR-cancers excluding lung and prostate	0.93 (0.70,1.23)	0.94 (0.71,1.25)	1.03 (0.95,1.1)	1.02 (0.95,1.10)
**Specific cancer sites**				
Colorectal	1.99 (1.38,2.86)	1.96 (1.36,2.83)	0.88 (0.78,1.00)	0.88 (0.78,1.00)
Kidney	1.02 (0.47,2.23)	1.02 (0.47,2.23)	0.89 (0.72,1.09)	0.89 (0.72,1.10)
Bladder	1.18 (0.56,2.46)	1.21 (0.58,2.52)	1.07 (0.86,1.33)	1.06 (0.85,1.32)
Pancreas	0.83 (0.35,1.98)	0.79 (0.33,1.9)	1.08 (0.86,1.37)	1.09 (0.87,1.38)
Lung	1.50 (1.11,2.05)	1.46 (1.07,2.00)	0.88 (0.80,0.96)	0.87 (0.80,0.96)
Prostate	0.99 (0.78,1.24)	0.93 (0.74,1.18)	1.02 (0.96,1.08)	1.03 (0.96,1.09)
Metastatic Prostate	1.68 (0.84,3.38)	1.59 (0.78,3.24)	0.98 (0.77,1.25)	0.99 (0.78,1.26)
**Women**				
All Cancers	1.00 (0.91,1.10)	0.99 (0.90,1.09)	1.02 (0.98,1.06)	1.03 (0.99,1.07)
OBR-cancers	1.02 (0.91,1.15)	1.02 (0.91,1.15)	1.04 (0.99,1.09)	1.04 (0.99,1.10)
NOBR-cancers	0.94 (0.80,1.10)	0.93 (0.80,1.09)	1 (0.94,1.07)	1.02 (0.96,1.09)
NOBR-cancers excluding lung	0.9 (0.74,1.10)	0.90 (0.74,1.09)	1.03 (0.95,1.12)	1.04 (0.96,1.13)
**Specific cancer sites**				
Colorectal	0.85 (0.62,1.17)	0.86 (0.63,1.18)	1.08 (0.95,1.23)	1.06 (0.93,1.21)
Pancreas	1.62 (1.03,2.56)	1.63 (1.04,2.56)	1.03 (0.82,1.30)	1.01 (0.80,1.27)
Kidney	1.07 (0.64,1.81)	1.08 (0.64,1.82)	0.93 (0.74,1.17)	0.92 (0.73,1.15)
Lung	1.00 (0.76,1.30)	0.98 (0.75,1.29)	0.97 (0.86,1.08)	0.99 (0.88,1.11)
Endometrial	1.01 (0.68,1.48)	1.01 (0.68,1.49)	1.02 (0.85,1.22)	1.04 (0.86,1.24)
Ovarian	1.33 (0.84,2.09)	1.32 (0.83,2.09)	1.12 (0.9,1.40)	1.13 (0.91,1.42)
Post-menopausal breast cancer	0.94 (0.79,1.12)	0.94 (0.79,1.12)	1.05 (0.97,1.13)	1.05 (0.98,1.13)

* Multivariable adjustment for baseline age, ethnicity, alcohol, smoking and HRT (in women).
* Degree of overweight is the cumulative sum of the number of BMI units ≥ 25 kg/m^2 over the exposure period.
* Duration of overweight is the cumulative sum of the duration overweight (BMI ≥ 25 kg/m^2) over the exposure period.
Abbreviations: OBR, obesity-related; NOBR, non-obesity related; CI, confidence interval; HR, hazard ratio; BMI, body mass index; MV, multivariable; SD, standard deviation.

Table S25: Comparison of associations of overweight-years at Visit 2 and BMI at Visit 2 with cancer by Akaike information criterion in the ARIC cohort.

	AIC				
**Characteristic**	**MV-adjusted overweight-years**	**MV-adjusted BMI**	**MV-adjusted overweight - years with BMI**	**MV-adjusted degree of overweight**	**MV-adjusted duration of overweight**
**Men**					
All cancers	31890.20	31889.19	31891.15	31887.41	31890.02
OBR-cancers	6287.36	6281.17	6281.92	6275.88	6289.52
NOBR-cancers	25610.82	25610.91	25612.77	25610.97	25610.65
NOBR-cancers excluding lung and prostate	8887.04	8887.07	8887.17	8887.66	8886.96
**Specific cancer sites**					
Colorectal	2690.94	2689.65	2683.27	2681.36	2701.00
Kidney	1050.09	1050.06	1052.02	1050.06	1049.62
Bladder	1061.22	1060.99	1061.80	1060.76	1061.25
Pancreas	959.15	959.44	959.68	959.96	958.97
Lung	4726.92	4715.70	4716.90	4723.73	4708.23
Prostate	11880.39	11879.95	11881.95	11880.27	11879.31
Metastatic Prostate	828.99	831.44	830.98	830.93	831.50
**Women**					
All cancers	29100.13	29073.64	29074.88	29080.04	29081.61
OBR-cancers	18100.31	18070.94	18072.67	18075.69	18080.61
NOBR-cancers	10944.52	10947.02	10944.58	10948.00	10946.92
NOBR-cancers excluding lung	7369.77	7367.77	7365.99	7370.56	7367.77
**Specific cancer sites**					
Colorectal	2901.67	2899.27	2900.64	2900.86	2898.93
Pancreas	865.50	869.93	866.79	867.55	870.61
Kidney	924.76	918.60	920.25	919.21	924.75
Lung	3518.54	3518.45	3519.57	3518.65	3518.84
Endometrial	1793.51	1753.27	1755.27	1759.60	1778.16
Ovarian	1062.46	1062.42	1064.06	1061.55	1061.21
Post-menopausal breast cancer	8842.04	8836.87	8838.81	8839.16	8835.98

* Multivariable adjustment for baseline age, ethnicity, alcohol, smoking and HRT (in women).
Abbreviations: SE, standard error; OBR, obesity-related; NOBR, non-obesity related; BMI, body mass index; AIC, Akaike information criterion; MV, multivariable.

Table S26: Comparison of the overweight-years metric at Visit 2 and BMI at Visit 2 using Harrell's C-statistic, ARIC

	Harrell's C-statistic (95% CI)								
**Characteristic**	**MV-adjusted overweight-years**	**MV-adjusted BMI**	**Difference in c-statistic between BMI and overweight-years**	**MV-adjusted overweight -years with BMI**	**Difference in c-statistic between MV-adjusted overweight-years with BMI combined compared with overweight-years**	**Difference in c-statistic between MV-adjusted overweight-years with BMI combined and MV-adjusted BMI**	**MV-adjusted cumulative overweight degree**	**MV-adjusted cumulative overweight duration**	**Difference in c-statistic between MV-adjusted duration and MV-adjusted degree**
**Men**									
All cancers	0.602 (0.593, 0.612)	0.602 (0.592, 0.611)	-0.001 (-0.009, 0.007)	0.603 (0.594, 0.612)	0.001 (-0.004, 0.0063)	0.002 (-0.006, 0.010)	0.603 (0.594, 0.613)	0.602 (0.593, 0.612)	-0.001 (-0.008, 0.006)
OBR-cancers	0.584 (0.567, 0.602)	0.593 (0.576, 0.611)	0.009 (-0.009, 0.027)	0.590 (0.573, 0.607)	0.008 (-0.008, 0.025)	-0.001 (-0.009, 0.0075)	0.597 (0.580, 0.614)	0.582 (0.565, 0.600)	-0.014 (-0.035, 0.006)
NOBR-cancers	0.608 (0.599, 0.618)	0.609 (0.600, 0.619)	0.008 (-0.004, 0.006)	0.610 (0.601, 0.620)	0.000 (-0.006, 0.007)	-0.000 (-0.007, 0.006)	0.609 (0.599, 0.619)	0.609 (0.599, 0.618)	-0.000 (-0.007, 0.006)
NOBR-cancers excluding lung and prostate	0.595 (0.580, 0.610)	0.5927 (0.5779, 0.6078)	-0.002 (-0.009, 0.005)	0.594 (0.579, 0.609)	-0.001 (-0.006, 0.004)	0.002 (-0.004, 0.007)	0.593 (0.578, 0.608)	0.594 (0.579, 0.609)	0.001 (-0.003, 0.006)
**Specific cancer sites**									
Colorectal	0.654 (0.626, 0.683)	0.6506 (0.6241, 0.6782)	-0.004 (-0.037, 0.030)	0.660 (0.633, 0.687)	0.004 (-0.022, 0.031)	0.008 (-0.009, 0.024)	0.661 (0.634, 0.689)	0.644 (0.617, 0.672)	-0.017 (-0.048, 0.014)
Kidney	0.609 (0.559, 0.664)	0.6099 (0.5597, 0.6646)	0.001 (-0.010, 0.012)	0.612 (0.562, 0.666)	0.003 (-0.008, 0.013)	0.002 (-0.004, 0.008)	0.607 (0.557, 0.662)	0.612 (0.562, 0.666)	0.004 (-0.021, 0.030)
Bladder	0.702 (0.661, 0.746)	0.7000 (0.6574, 0.7454)	-0.002 (-0.027, 0.023)	0.706 (0.663, 0.751)	0.004 (-0.013, 0.021)	0.006 (-0.012, 0.024)	0.703 (0.661, 0.749)	0.694 (0.652, 0.739)	-0.009 (-0.030, 0.012)
Pancreas	0.604 (0.558, 0.654)	0.5991 (0.5520, 0.6502)	-0.005 (-0.034, 0.025)	0.622 (0.574, 0.673)	0.018 (-0.010, 0.046)	0.023 (0.010, 0.036)	0.587 (0.541, 0.638)	0.609 (0.562, 0.660)	0.021 (-0.000, 0.043)
Lung	0.722 (0.704, 0.740)	0.7317 (0.7135, 0.7505)	0.010 (-0.003, 0.023)	0.733 (0.715, 0.752)	0.012 (-0.002, 0.024)	0.001 (-0.001, 0.004)	0.725 (0.707, 0.743)	0.739 (0.721, 0.757)	0.014 (0.0032, 0.0246)
Prostate	0.614 (0.601, 0.627)	0.6141 (0.6011, 0.6273)	0.000 (-0.004, 0.004)	0.613 (0.600, 0.627)	0.000 (-0.003, 0.003)	0.000 (-0.003, 0.003)	0.614 (0.601, 0.627)	0.614 (0.601, 0.628)	0.000 (-0.004, 0.004)
Metastatic Prostate	0.640 (0.588, 0.695)	0.6187 (0.5720, 0.6693)	-0.021 (-0.057, 0.016)	0.638 (0.589, 0.695)	-0.001 (-0.003, 0.001)	0.020 (-0.017, 0.057)	0.621 (0.573, 0.673)	0.619 (0.572, 0.669)	-0.002 (-0.023, 0.019)
**Women**									
All cancers	0.576 (0.566, 0.585)	0.5840 (0.5745, 0.5936)	0.008 (-0.002, 0.019)	0.584 (0.574, 0.594)	0.009 (-0.003, 0.020)	0.001 (-0.004, 0.005)	0.582 (0.572, 0.591)	0.585 (0.575, 0.595)	0.004 (-0.004, 0.012)
OBR-cancers	0.550 (0.540, 0.561)	0.5751 (0.5641, 0.5863)	0.025 (0.009, 0.041)	0.568 (0.553, 0.585)	0.025 (0.007, 0.042)	-0.000 (-0.008, 0.008)	0.570 (0.559, 0.581)	0.574 (0.563, 0.585)	0.004 (-0.009, 0.017)
NOBR-cancers	0.640 (0.626, 0.654)	0.6393 (0.6258, 0.6531)	-0.000 (-0.005, 0.004)	0.641 (0.627, 0.655)	0.001 (-0.001, 0.004)	0.002 (-0.002, 0.005)	0.639 (0.625, 0.652)	0.641 (0.628, 0.655)	0.003 (0.001, 0.004)
NOBR-cancers excluding lung	0.594 (0.578, 0.611)	0.5985 (0.5827, 0.6148)	0.005 (-0.010, 0.019)	0.602 (0.586, 0.619)	0.008 (-0.003, 0.019)	0.004 (-0.006, 0.013)	0.594 (0.578, 0.610)	0.605 (0.589, 0.621)	0.011 (0.001, 0.021)
**Specific cancer sites**									
Colorectal	0.552 (0.527, 0.579)	0.5915 (0.5660, 0.6182)	0.039 (0.001, 0.077)	0.587 (0.562, 0.614)	0.035 (0.001, 0.068)	-0.005 (-0.020, 0.011)	0.583 (0.558, 0.609)	0.583 (0.559, 0.609)	0.000 (-0.026, 0.027)
Pancreas	0.656 (0.611, 0.705)	0.6479 (0.5984, 0.7014)	-0.008 (-0.068, 0.052)	0.670 (0.622, 0.723)	0.014 (-0.021, 0.049)	0.023 (-0.018, 0.063)	0.661 (0.611, 0.715)	0.635 (0.590, 0.682)	-0.026 (-0.071, 0.019)
Kidney	0.646 (0.592, 0.705)	0.6675 (0.6124, 0.7275)	0.021 (-0.031, 0.073)	0.667 (0.612, 0.727)	0.021 (-0.027, 0.068)	-0.001 (-0.011, 0.010)	0.664 (0.607, 0.726)	0.640 (0.589, 0.696)	-0.023 (-0.065, 0.019)

Lung	0.756 (0.7352, 0.7763)	0.7547 (0.7341, 0.7759)	-0.001 (-0.007, 0.006)	0.756 (0.736, 0.777)	0.001 (-0.004, 0.005)	0.001 (-0.003, 0.005)	0.755 (0.735, 0.777)	0.753 (0.732, 0.774)	-0.003 (-0.006, 0.001)
Endometrial	0.601 (0.571, 0.633)	0.6825 (0.6455, 0.7216)	0.082 (0.030, 0.133)	0.683 (0.646, 0.722)	0.082 (0.030, 0.133)	0.000 (-0.000, 0.000)	0.681 (0.645, 0.719)	0.653 (0.618, 0.689)	-0.028 (-0.066, 0.009)
Ovarian	0.593 (0.552, 0.637)	0.5799 (0.5385, 0.6245)	-0.013 (-0.052, 0.026)	0.593 (0.552, 0.637)	0.000 (-0.020, 0.012)	0.013 (-0.014, 0.040)	0.587 (0.546, 0.631)	0.611 (0.570, 0.655)	0.024 (-0.014, 0.062)
Post-menopausal breast cancer	0.594 (0.579, 0.609)	0.6036 (0.5893, 0.6183)	0.010 (-0.004, 0.024)	0.602 (0.588, 0.617)	0.009 (-0.004, 0.023)	-0.001 (-0.004, 0.003)	0.597 (0.583, 0.612)	0.607 (0.593, 0.623)	0.011 (-0.001, 0.022)

* Multivariable adjustment for baseline age, ethnicity, alcohol, smoking and HRT (in women).

Abbreviations: SE, standard error; OBR, obesity-related; NOBR, non-obesity related; BMI, body mass index; AIC, Akaike information criterion.

Key: Green – significant difference in C-statistic.

a) Analysis of obese-years exposure

Table S27: Incidence of cancer (events/1000 Person-Years) according to obese-years at Visit 2 and BMI at Visit 2 by gender, ethnicity, smoking, HRT (women only) in the ARIC cohort.

	**0 obese-years (kg-years/m^2)**			**>0-100 obese-years (kg-years/m^2)**			**>100 obese-years (kg-years/m^2)**			**Baseline BMI (kg /m^2)**		
	**N**	**PYFU**	**IR (95% CI)**	**N**	**PYFU**	**IR (95% CI)**	**N**	**PYFU**	**IR (95% CI)**	**N**	**PYFU**	**IR (95% CI)**
**Men**												
**Whole sample**	1496	308521.30	4.85 (4.60, 5.10)	529	100832.70	5.25 (4.79, 5.70)	47	8333.27	5.64 (3.96, 7.32)	2072	417687.30	4.96 (4.75 ,5.18)
**Ethnicity**												
**White**	1183	255895.50	4.62 (4.36, 4.89)	389	78673.83	4.94 (4.45, 5.44)	35	6220.64	5.63 (3.60, 7.58)	1607	340790.00	4.72 (4.48 ,4.95)
**Black**	313	52625.79	5.95 (5.28, 6.62)	140	22158.85	6.32 (5.25, 7.39)	12	2112.63	5.68 (2.19, 9.17)	465	76897.27	6.05 (5.49, 6.60)
**Smoking**												
**Ever**	1139	222600.30	5.12 (4.82, 5.41)	396	72700.11	5.45 (4.90, 5.99)	31	5915.17	5.24 (3.30, 7.18)	1566	301215.60	5.20 (4.94, 5.46)
**Never**	357	85921.00	4.16 (3.72, 4.60)	133	28132.57	4.73 (3.91, 5.55)	16	2418.17	6.62 (3.14, 10.10)	506	116471.70	4.35 (3.96, 4.73)
**Women**												
**Whole sample**	1021	355285.60	2.87 (-2.76, 8.51)	627	199385.30	0.13 (-0.12, 0.38)	156	53131.84	2.94 (2.47, 3.41)	1804	607802.70	2.97 (2.83, 3.11)
**Ethnicity**												
**White**	824	282519.60	2.92 (2.72, 3.12)	400	122894.10	0.16 (-0.16, 0.49)	112	40024.10	2.80 (2.27, 3.33)	1336	445437.80	3.00 (2.84 ,3.16)
**Black**	197	72765.97	2.71 (2.32, 3.09)	227	76491.20	0.20 (-0.19, 0.59)	44	13107.73	3.36 (2.32, 4.39)	468	162364.90	2.88 (2.62 ,3.15)
**Smoking**												
**Ever**	596	179604.30	3.32 (3.05, 3.59)	306	88804.67	0.20 (-0.19, 0.59)	82	24470.69	3.35 (2.60, 4.10)	984	292879.60	3.36 (3.15, 3.57)
**Never**	425	175681.30	2.42 (2.19, 2.65)	321	110580.60	0.16 (-0.16, 0.49)	74	28661.15	2.58 (1.97, 3.19)	820	314923.00	2.60 (2.42 ,2.78)
**HRT**												
**Ever**	428	144959.10	2.95 (2.67, 3.24)	163	59089.39	0.22 (-0.21, 0.65)	37	17176.60	2.15 (1.43, 2.88)	628	221225.10	2.84 (2.61 ,3.06)
**Never**	593	210326.50	2.82 (2.59, 3.05)	464	140295.90	0.16 (-0.15, 0.46)	119	35955.24	3.31 (2.70, 3.92)	1176	386577.60	3.04 (2.87, 3.22)

* Multivariable adjustment for baseline age, ethnicity, alcohol, smoking and HRT (in women).

Abbreviations: N, number of events; PYFR, person-years of follow-up; IR, incidence rate of all cancers; BMI, body mass index

Table S28: Hazard ratio of cancers by obese-years at Visit 2 and BMI at Visit 2 in ARIC.

Outcomes	Obese-years (kg-years/m^2) (per SD)		BMI (kg /m^2) (SD)	
	Age-adjusted HR (95% CI)	MV-adjusted HR (95% CI)	Age-adjusted HR (95% CI)	MV-adjusted HR (95% CI)
**Men**				
All Cancers	1.01 (0.97,1.05)	1.01 (0.97,1.05)	1.02 (0.98,1.07)	1.02 (0.98,1.07)
OBR-cancers	1.06 (0.98,1.13)	1.05 (0.98,1.13)	1.16 (1.05,1.27)	1.15 (1.05,1.27)
NOBR-cancers	0.99 (0.94,1.04)	0.99 (0.94,1.04)	0.99 (0.94,1.04)	0.99 (0.95,1.04)
NOBR-cancers excluding lung and prostate	0.99 (0.91,1.07)	0.99 (0.92,1.08)	1.05 (0.96,1.14)	1.05 (0.97,1.14)
**Specific cancer sites**				
Colorectal	1.12 (1.04,1.20)	1.11 (1.04,1.20)	1.28 (1.12,1.47)	1.28 (1.12,1.47)
Kidney	1.04 (0.86,1.26)	1.03 (0.85,1.25)	1.03 (0.81,1.32)	1.03 (0.81,1.32)
Bladder	1.1 (0.97,1.25)	1.12 (0.98,1.28)	1.17 (0.93,1.47)	1.17 (0.93,1.48)
Pancreas	0.79 (0.47,1.32)	0.78 (0.47,1.32)	1.13 (0.89,1.45)	1.13 (0.89,1.45)
Lung	0.98 (0.88,1.1)	0.99 (0.89,1.11)	0.80 (0.70,0.90)	0.81 (0.72,0.92)
Prostate	0.99 (0.93,1.06)	0.98 (0.92,1.06)	1.03 (0.96,1.11)	1.02 (0.95,1.10)
Metastatic Prostate	1.13 (0.98,1.29)	1.12 (0.97,1.29)	1.04 (0.79,1.36)	1.04 (0.79,1.35)

**Women**				
All Cancers	1.01 (0.97,1.06)	1.01 (0.97,1.06)	1.11 (1.06,1.16)	1.14 (1.08,1.19)
OBR-cancers	1.04 (0.99,1.10)	1.04 (0.99,1.10)	1.18 (1.11,1.24)	1.19 (1.12,1.26)
NOBR-cancers	0.96 (0.89,1.04)	0.97 (0.90,1.05)	0.99 (0.91,1.07)	1.05 (0.97,1.14)
NOBR-cancers excluding lung	0.97 (0.88,1.06)	0.97 (0.88,1.07)	1.06 (0.97,1.17)	1.12 (1.01,1.23)
**Specific cancer sites**				
Colorectal	0.95 (0.81,1.11)	0.95 (0.81,1.11)	1.19 (1.03,1.36)	1.14 (0.98,1.32)
Pancreas	1.21 (1.05,1.40)	1.21 (1.04,1.40)	1.28 (1.01,1.64)	1.16 (0.89,1.52)
Kidney	1.11 (0.92,1.35)	1.11 (0.92,1.35)	1.45 (1.17,1.81)	1.4 (1.10,1.77)
Lung	0.95 (0.83,1.09)	0.97 (0.84,1.11)	0.83 (0.72,0.96)	0.92 (0.79,1.07)
Endometrial	1.06 (0.9,1.26)	1.06 (0.9,1.26)	1.60 (1.38,1.86)	1.75 (1.49,2.05)
Ovarian	1.08 (0.88,1.33)	1.09 (0.88,1.33)	1.01 (0.79,1.30)	1.1 (0.85,1.42)
Post-menopausal breast cancer	1.02 (0.94,1.10)	1.02 (0.94,1.10)	1.08 (1.00,1.18)	1.11 (1.02,1.21)

* Multivariable adjustment for baseline age, ethnicity, alcohol, smoking and HRT (in women).
Abbreviations: OBR, obesity-related; NOBR, non-obesity related; CI, confidence interval; HR, hazard ratio; BMI, body mass index; MV, multivariable; SD, standard deviation; BMI, body mass index.

Table S29: Hazard ratio of cancers by obese-years per 100 units at Visit 2 and BMI per 5 units (kg/m^2) at Visit 2 in ARIC.

Outcomes	Obese-years (per 100 (kg-years/m^2))		BMI (per 5 unit [kg/m^2])	
	Age-adjusted HR (95% CI)	MV-adjusted HR (95% CI)	Age-adjusted HR (95% CI)	MV-adjusted HR (95% CI)
**Men**				
All Cancers	1.02 (0.91,1.15)	1.02 (0.9,1.14)	1.03 (0.97,1.08)	1.03 (0.98,1.08)
OBR-cancers	1.17 (0.96,1.44)	1.16 (0.95,1.43)	1.19 (1.06,1.33)	1.19 (1.06,1.33)
NOBR-cancers	0.97 (0.84,1.12)	0.97 (0.84,1.12)	0.99 (0.93,1.05)	0.99 (0.94,1.05)
NOBR-cancers excluding lung and prostate	0.97 (0.76,1.23)	0.98 (0.78,1.25)	1.05 (0.95,1.17)	1.06 (0.96,1.18)
**Specific cancer sites**				
Colorectal	1.39 (1.12,1.71)	1.37 (1.11,1.70)	1.35 (1.15,1.59)	1.34 (1.14,1.58)
Kidney	1.12 (0.63,2.01)	1.10 (0.62,1.96)	1.04 (0.77,1.39)	1.04 (0.77,1.39)
Bladder	1.34 (0.92,1.95)	1.40 (0.94,2.08)	1.20 (0.91,1.59)	1.21 (0.92,1.60)
Pancreas	0.50 (0.11,2.30)	0.49 (0.10,2.27)	1.16 (0.87,1.56)	1.16 (0.87,1.55)
Lung	0.95 (0.69,1.32)	0.98 (0.71,1.37)	0.76 (0.66,0.88)	0.78 (0.67,0.90)
Prostate	0.98 (0.80,1.21)	0.95 (0.78,1.17)	1.04 (0.95,1.13)	1.03 (0.95,1.12)
Metastatic prostate cancer	1.43 (0.95,2.15)	1.40 (0.93,2.11)	1.05 (0.76,1.45)	1.04 (0.76,1.43)

**Women**				
All Cancers	1.02 (0.96,1.08)	1.02 (0.96,1.09)	1.09 (1.05,1.13)	1.11 (1.07,1.16)
OBR-cancers	1.06 (0.98,1.14)	1.06 (0.98,1.14)	1.14 (1.09,1.20)	1.15 (1.10,1.21)
NOBR-cancers	0.95 (0.85,1.06)	0.96 (0.86,1.07)	0.99 (0.93,1.05)	1.04 (0.97,1.11)
NOBR-cancers excluding lung	0.95 (0.83,1.09)	0.96 (0.84,1.10)	1.05 (0.98,1.14)	1.09 (1.01,1.19)
**Specific cancer sites**				
Colorectal	0.93 (0.74,1.16)	0.93 (0.74,1.16)	1.15 (1.03,1.29)	1.11 (0.98,1.26)
Pancreas	1.31 (1.07,1.62)	1.31 (1.06,1.62)	1.23 (1.01,1.50)	1.13 (0.91,1.41)
Kidney	1.16 (0.89,1.53)	1.17 (0.88,1.54)	1.36 (1.14,1.63)	1.32 (1.09,1.60)
Lung	0.94 (0.77,1.14)	0.95 (0.78,1.16)	0.86 (0.76,0.97)	0.93 (0.82,1.06)
Endometrial	1.09 (0.86,1.38)	1.09 (0.86,1.38)	1.47 (1.30,1.67)	1.59 (1.39,1.81)
Ovarian	1.02 (0.91,1.15)	1.02 (0.91,1.15)	1.07 (1.00,1.14)	1.09 (1.01,1.17)
Post-menopausal breast	0.93 (0.74,1.16)	0.93 (0.74,1.16)	1.15 (1.03,1.29)	1.11 (0.98,1.26)

* Multivariable adjustment for baseline age, ethnicity, alcohol, smoking and HRT (in women).
Abbreviations: OBR, obesity-related; NOBR, non-obesity related; CI, confidence interval; HR, hazard ratio; BMI, body mass index; MV, multivariable.

Table S30: Hazard ratio of cancers by obesity degree and duration at Visit 2, ARIC.

Outcome	Degree of Obesity (kg /m^2) (per SD)		Duration of Obesity (years) (per SD)	
	Age-adjusted HR (95% CI)	MV-adjusted HR (95% CI)	Age-adjusted HR (95% CI)	MV-adjusted HR (95% CI)
**Men**				
All Cancers	1.10 (1.04,1.18)	1.09 (1.02,1.16)	1.06 (1.00,1.13)	1.05 (0.99,1.11)
OBR-cancers	1.23 (1.09,1.40)	1.22 (1.08,1.38)	1.08 (0.95,1.23)	1.08 (0.95,1.23)
NOBR-cancers	1.06 (0.98,1.14)	1.04 (0.97,1.13)	1.06 (0.99,1.13)	1.04 (0.98,1.12)
NOBR-cancers excluding lung and prostate	1.01 (0.89,1.15)	1.01 (0.89,1.15)	1.00 (0.89,1.12)	1.00 (0.89,1.12)
**Specific cancer sites**				
Colorectal	1.26 (1.06,1.51)	1.25 (1.05,1.50)	1.05 (0.87,1.27)	1.05 (0.87,1.26)
Kidney	1.17 (0.85,1.63)	1.18 (0.85,1.63)	0.80 (0.55,1.17)	0.81 (0.56,1.17)
Bladder	0.79 (0.52,1.19)	0.80 (0.53,1.21)	0.98 (0.71,1.35)	0.98 (0.71,1.36)
Pancreas	0.91 (0.61,1.36)	0.90 (0.60,1.33)	1.41 (1.02,1.95)	1.39 (1.00,1.92)
Lung	1.25 (1.06,1.47)	1.23 (1.04,1.46)	1.21 (1.03,1.41)	1.17 (1.00,1.36)
Prostate	1.01 (0.91,1.13)	0.99 (0.88,1.10)	1.05 (0.95,1.15)	1.03 (0.93,1.13)
Metastatic prostate cancer	1.10 (0.75,1.61)	1.07 (0.73,1.56)	0.90 (0.61,1.33)	0.88 (0.59,1.30)
**Women**				
All Cancers	1.02 (0.94,1.10)	1.01 (0.93,1.09)	1.01 (0.95,1.08)	1.02 (0.95,1.09)
OBR-cancers	1.00 (0.91,1.10)	1.00 (0.91,1.10)	1.01 (0.93,1.11)	1.02 (0.93,1.11)
NOBR-cancers	1.03 (0.91,1.17)	1.01 (0.89,1.15)	1.02 (0.91,1.14)	1.02 (0.91,1.14)
NOBR-cancers excluding lung	0.99 (0.85,1.16)	0.98 (0.84,1.15)	1.03 (0.90,1.18)	1.03 (0.90,1.18)
**Specific cancer sites**				
Colorectal	0.87 (0.67,1.12)	0.88 (0.69,1.14)	1.07 (0.87,1.32)	1.07 (0.87,1.32)
Pancreas	1.30 (0.88,1.92)	1.33 (0.91,1.95)	1.00 (0.69,1.45)	0.99 (0.68,1.44)
Kidney	1.07 (0.72,1.59)	1.08 (0.73,1.61)	0.99 (0.69,1.42)	0.99 (0.70,1.42)
Lung	1.07 (0.85,1.35)	1.04 (0.82,1.32)	1.02 (0.83,1.25)	1.01 (0.82,1.24)
Endometrial	1.01 (0.76,1.35)	1.01 (0.75,1.36)	0.90 (0.68,1.18)	0.90 (0.68,1.18)
Ovarian	1.25 (0.85,1.83)	1.23 (0.83,1.82)	0.83 (0.55,1.23)	0.82 (0.55,1.23)
Post-menopausal breast cancer	0.92 (0.80,1.07)	0.92 (0.79,1.06)	1.05 (0.93,1.19)	1.05 (0.93,1.19)

* Multivariable adjustment for baseline age, ethnicity, alcohol, smoking and HRT (in women).
* Degree of obese is the cumulative sum of the number of BMI units ≥ 30 kg/m^2 over the exposure period.
* Duration of obese is the cumulative sum of the duration overweight (BMI ≥ 30 kg/m^2) over the exposure period.
Abbreviations: OBR, obesity-related; NOBR, non-obesity related; CI, confidence interval; HR, hazard ratio; BMI, body mass index; MV, multivariable; SD, standard deviation.

Table S31: Hazard ratio of cancers by obesity degree and duration per 10 units and per 10 years, respectively at Visit 2, ARIC.

Outcome	Degree of obesity (per 10 (kg /m^2)		Duration of obesity (years) (per 10 years)	
	Age-adjusted HR (95% CI)	MV-adjusted HR (95% CI)	Age-adjusted HR (95% CI)	MV-adjusted HR (95% CI)
**Men**				
All Cancers	1.26 (1.09,1.46)	1.22 (1.05,1.42)	1.05 (1.00,1.09)	1.04 (0.99,1.08)
OBR-cancers	1.63 (1.23,2.18)	1.6 (1.20,2.14)	1.06 (0.96,1.17)	1.06 (0.96,1.16)
NOBR-cancers	1.15 (0.96,1.36)	1.11 (0.93,1.32)	1.04 (0.99,1.10)	1.03 (0.98,1.09)
NOBR-cancers excluding lung and prostate	1.02 (0.76,1.38)	1.04 (0.77,1.40)	1.00 (0.92,1.09)	1.00 (0.92,1.09)
**Specific cancer sites**				
Colorectal	1.72 (1.13,2.61)	1.69 (1.11,2.57)	1.04 (0.90,1.20)	1.04 (0.90,1.19)
Kidney	1.46 (0.68,3.11)	1.46 (0.68,3.14)	0.85 (0.64,1.12)	0.85 (0.64,1.13)
Bladder	0.57 (0.22,1.50)	0.59 (0.22,1.56)	0.99 (0.77,1.26)	0.99 (0.78,1.26)
Pancreas	0.8 (0.32,2.03)	0.78 (0.31,1.95)	1.29 (1.01,1.65)	1.28 (1.00,1.63)
Lung	1.68 (1.14,2.47)	1.63 (1.10,2.42)	1.15 (1.03,1.29)	1.13 (1.00,1.26)
Prostate	1.03 (0.80,1.32)	0.97 (0.75,1.25)	1.03 (0.96,1.11)	1.02 (0.95,1.10)
Metastatic prostate cancer	1.24 (0.51,3.02)	1.17 (0.48,2.84)	0.92 (0.69,1.24)	0.91 (0.68,1.22)
**Women**				
All Cancers	1.03 (0.92,1.14)	1.01 (0.91,1.12)	1.01 (0.96,1.06)	1.01 (0.96,1.06)
OBR-cancers	1.00 (0.88,1.14)	1.00 (0.88,1.14)	1.01 (0.95,1.07)	1.01 (0.95,1.07)
NOBR-cancers	1.04 (0.87,1.24)	1.01 (0.85,1.21)	1.01 (0.94,1.10)	1.01 (0.94,1.10)
NOBR-cancers excluding lung	0.99 (0.80,1.23)	0.97 (0.79,1.20)	1.02 (0.93,1.12)	1.02 (0.93,1.12)
**Specific cancer sites**				
Colorectal	0.82 (0.59,1.16)	0.85 (0.60,1.19)	1.05 (0.91,1.22)	1.05 (0.91,1.21)
Pancreas	1.42 (0.84,2.40)	1.47 (0.87,2.46)	1.00 (0.77,1.29)	0.99 (0.77,1.29)
Kidney	1.10 (0.64,1.87)	1.11 (0.65,1.90)	0.99 (0.78,1.27)	0.99 (0.78,1.27)
Lung	1.10 (0.80,1.50)	1.05 (0.77,1.45)	1.01 (0.88,1.17)	1.01 (0.87,1.16)
Endometrial	1.01 (0.69,1.50)	1.01 (0.68,1.51)	0.93 (0.77,1.12)	0.93 (0.76,1.12)
Ovarian	1.34 (0.80,2.25)	1.32 (0.78,2.24)	0.88 (0.66,1.16)	0.87 (0.66,1.16)
Post-menopausal breast cancer	0.89 (0.73,1.09)	0.89 (0.73,1.08)	1.04 (0.95,1.13)	1.04 (0.95,1.13)

* Multivariable adjustment for baseline age, ethnicity, alcohol, smoking and HRT (in women).
* Degree of obese is the cumulative sum of the number of BMI units ≥ 30 kg/m^2 over the exposure period.
* Duration of obese is the cumulative sum of the duration overweight (BMI ≥ 30 kg/m^2) over the exposure period.

Abbreviations: OBR, obesity-related; NOBR, non-obesity related; CI, confidence interval; HR, hazard ratio; BMI, body mass index; MV, multivariable; SD, standard deviation.

Table S32: Comparison of associations of obese-years at Visit 2 and BMI at Visit 2 with cancer by Akaike information criterion in the ARIC cohort.

	AIC				
**Characteristic**	**MV-adjusted obese-years**	**MV-adjusted BMI**	**MV-adjusted obese -years with BMI**	**MV-adjusted degree of obesity**	**MV-adjusted duration of obesity**
**Men**					
All cancers	31890.24	31889.19	31891.16	31883.92	31886.70
OBR-cancers	6287.79	6281.17	6282.08	6272.13	6281.39
NOBR-cancers	25610.78	25610.91	25612.73	25610.63	25610.49
NOBR-cancers excluding lung and prostate	8888.44	8887.07	8889.01	8887.40	8887.74
**Specific cancer sites**					
Colorectal	2695.54	2689.65	2687.02	2684.27	2693.42
Kidney	1050.02	1050.06	1051.97	1049.48	1049.71
Bladder	1060.80	1060.99	1061.30	1062.67	1061.98
Pancreas	959.01	959.44	959.39	960.28	956.17
Lung	4727.17	4715.7	4717.68	4726.76	4726.36
Prostate	11880.20	11879.95	11881.67	11880.29	11879.67
Metastatic prostate cancer	829.80	831.44	831.78	831.34	831.43
**Women**					
All cancers	29099.75	29073.64	29075.64	29081.88	29083.42
OBR-cancers	18099.79	18070.94	18072.39	18081.35	18082.94
NOBR-cancers	10947.68	10947.02	10948.13	10947.38	10947.22
NOBR-cancers excluding lung	7372.08	7367.77	7368.93	7370.03	7369.00
**Specific cancer sites**					
Colorectal	2901.51	2899.27	2900.41	2901.36	2899.32
Pancreas	866.63	869.93	867.86	868.29	870.54
Kidney	924.64	918.60	920.18	919.57	922.10
Lung	3519.38	3518.45	3520.28	3519.27	3519.11
Endometrial	1793.95	1753.27	1755.17	1763.12	1777.28
Ovarian	1062.38	1062.42	1063.98	1061.53	1062.90
Post-menopausal breast cancer	8841.88	8836.87	8838.85	8840.79	8837.10

* Multivariable adjustment for baseline age, ethnicity, alcohol, smoking and HRT (in women).
Abbreviations: SE, standard error; OBR, obesity-related; NOBR, non-obesity related; BMI, body mass index; AIC, Akaike information criterion; MV, multivariable.

Table S33: Comparison of the obese-years metric at Visit 2 and BMI at Visit 2 using Harrell's C-statistic, ARIC.

	Harrell's C-statistic (95% CI)								
**Characteristic**	**MV-adjusted obese-years**	**MV-adjusted BMI**	**Difference in c-statistic between BMI and obese-years**	**MV-adjusted obese -years with BMI**	**Difference in c-statistic between MV-adjusted obese-years with BMI combined compared with obese-years**	**Difference in c-statistic between MV-adjusted obese-years with BMI combined and MV-adjusted BMI**	**MV-adjusted cumulative obese degree**	**MV-adjusted cumulative obese duration**	**Difference in c-statistic between MV-adjusted duration and MV-adjusted degree**
**Men**									
All cancers	0.601 (0.592, 0.611)	0.603 (0.593, 0.613)	0.001 (-0.006, 0.008)	0.600 (0.591, 0.610)	-0.001 (-0.011, 0.008)	-0.003 (-0.012, 0.007)	0.604 (0.595, 0.614)	0.604 (0.595, 0.613)	-0.000 (-0.010, 0.010)
OBR-cancers	0.590 (0.572, 0.608)	0.592 (0.575, 0.610)	0.003 (-0.017, 0.023)	0.592 (0.575, 0.610)	0.005 (-0.014, 0.025)	0.003 (-0.003, 0.008)	0.600 (0.582, 0.618)	0.596 (0.578, 0.614)	-0.004 (-0.017, 0.010)
NOBR-cancers	0.610 (0.600, 0.620)	0.609 (0.599, 0.619)	-0.001 (-0.007, 0.005)	0.608 (0.598, 0.618)	-0.002 (-0.008, 0.005)	-0.001 (-0.005, 0.004)	0.610 (0.600, 0.620)	0.608 (0.599, 0.618)	-0.02 (-0.008, 0.004)
NOBR-cancers excluding lung and prostate	0.594 (0.579, 0.609)	0.593 (0.578, 0.608)	-0.001 (-0.006, 0.003)	0.593 (0.578, 0.608)	-0.001 (-0.006, 0.003)	-0.000 (-0.001, 0.001)	0.593 (0.578, 0.608)	0.593 (0.579, 0.608)	0.000 (-0.002, 0.003)
**Specific cancer sites**									
Colorectal	0.653 (0.626, 0.681)	0.650 (0.624, 0.678)	-0.002 (-0.036, 0.031)	0.658 (0.629, 0.686)	0.003 (-0.030, 0.034)	0.005 (-0.007, 0.017)	0.672 (0.646, 0.700)	0.658 (0.631, 0.686)	-0.015 (-0.030, 0.000)
Kidney	0.609 (0.558, 0.665)	0.610 (0.560, 0.665)	0.001 (-0.010, 0.011)	0.611 (0.561, 0.667)	0.002 (-0.006, 0.010)	0.001 (-0.005, 0.007)	0.609 (0.559, 0.664)	0.618 (0.569, 0.670)	0.008 (-0.019, 0.036)
Bladder	0.700 (0.659, 0.745)	0.700 (0.657, 0.745)	-0.000 (-0.025, 0.024)	0.706 (0.663, 0.752)	0.005 (-0.012, 0.023)	0.006 (-0.011, 0.022)	0.698 (0.656, 0.742)	0.697 (0.656, 0.740)	-0.001 (-0.013, 0.012)
Pancreas	0.598 (0.552, 0.647)	0.599 (0.552, 0.650)	0.001 (-0.027, 0.029)	0.618 (0.571, 0.669)	0.020 (-0.009, 0.048)	0.019 (0.012, 0.025)	0.584 (0.538, 0.633)	0.614 (0.563, 0.669)	0.030 (-0.007, 0.067)
Lung	0.721 (0.704, 0.739)	0.732 (0.715, 0.7504)	0.010 (-0.002, 0.023)	0.7312 (0.714, 0.751)	0.011 (-0.002, 0.023)	0.000 (-0.000, 0.001)	0.722 (0.704, 0.740)	0.722 (0.704, 0.740)	0.000 (-0.002, 0.003)
Prostate	0.612 (0.599, 0.626)	0.615 (0.602, 0.628)	0.003 (-0.002, 0.007)	0.613 (0.600, 0.626)	0.002 (-0.002, 0.005)	-0.001 (-0.005, 0.002)	0.615 (0.602, 0.628)	0.614 (0.601, 0.627)	-0.001 (-0.004, 0.002)
Metastatic prostate	0.634 (0.586, 0.686)	0.619 (0.572, 0.669)	-0.015 (-0.036, 0.006)	0.632 (0.584, 0.684)	-0.002 (-0.006, 0.002)	0.013 (-0.008, 0.035)	0.621 (0.574, 0.671)	0.622 (0.576, 0.672)	0.002 (-0.015, 0.018)
**Women**									
All cancers	0.574 (0.564, 0.584)	0.584 (0.574, 0.594)	0.010 (-0.002, 0.023)	0.585 (0.575, 0.594)	0.011 (0.001, 0.022)	0.001 (-0.010, 0.012)	0.579 (0.570, 0.588)	0.580 (0.570, 0.590)	0.001 (-0.007, 0.009)
OBR-cancers	0.549 (0.538, 0.560)	0.575 (0.564, 0.586)	0.026 (0.010, 0.043)	0.563 (0.549, 0.577)	0.026 (0.011, 0.041)	-0.000 (-0.004, 0.004)	0.562 (0.551, 0.573)	0.567 (0.556, 0.578)	0.005 (-0.006, 0.017)
NOBR-cancers	0.639 (0.625, 0.653)	0.639 (0.626, 0.653)	0.001 (-0.002, 0.003)	0.640 (0.626, 0.654)	0.001 (-0.001, 0.004)	0.001 (-0.001, 0.002)	0.638 (0.625, 0.652)	0.640 (0.626, 0.653)	0.001 (-0.000, 0.003)
NOBR-cancers excluding lung	0.591 (0.575, 0.608)	0.599 (0.583, 0.615)	0.008 (-0.004, 0.019)	0.599 (0.583, 0.615)	0.008 (-0.003, 0.019)	0.001 (-0.004, 0.005)	0.593 (0.577, 0.610)	0.598 (0.582, 0.614)	0.004 (-0.004, 0.013)
**Specific cancer sites**									
Colorectal	0.557 (0.531, 0.584)	0.592 (0.566, 0.618)	0.035 (-0.004, 0.074)	0.590 (0.564, 0.617)	0.033 (-0.000, 0.066)	-0.002 (-0.020, 0.016)	0.575 (0.550, 0.602)	0.579 (0.554, 0.605)	0.004 (-0.018, 0.026)
Pancreas	0.640 (0.596, 0.687)	0.648 (0.598, 0.701)	0.008 (-0.042, 0.058)	0.661 (0.612, 0.714)	0.021 (-0.017, 0.059)	0.013 (-0.011, 0.037)	0.657 (0.608, 0.710)	0.637 (0.593, 0.685)	-0.020 (-0.051, 0.010)
Kidney	0.645 (0.591, 0.705)	0.668 (0.612, 0.728)	0.022 (-0.028, 0.072)	0.665 (0.610, 0.726)	0.020 (-0.026, 0.066)	-0.002 (-0.012, 0.007)	0.664 (0.606, 0.727)	0.655 (0.600, 0.716)	-0.008 (-0.031, 0.015)
Lung	0.755 (0.735, 0.776)	0.755 (0.734, 0.776)	-0.001 (-0.006, 0.004)	0.755 (0.735, 0.777)	0.000 (-0.005, 0.005)	0.001 (-0.001, 0.002)	0.756 (0.735, 0.777)	0.755 (0.734, 0.776)	-0.001 (-0.004, 0.001)
Endometrial	0.598 (0.567, 0.631)	0.683 (0.646, 0.722)	0.084 (0.033, 0.135)	0.681 (0.644, 0.721)	0.083 (0.031, 0.135)	-0.001 (-0.005, 0.003)	0.664 (0.627, 0.704)	0.633 (0.596, 0.671)	-0.032 (-0.063, -0.001)

Ovarian	0.593 (0.552, 0.637)	0.580 (0.539, 0.625)	-0.013 (-0.048, 0.023)	0.593 (0.552, 0.638)	0.001 (-0.019, 0.021)	0.014 (-0.011, 0.038)	0.578 (0.536, 0.622)	0.578 (0.537, 0.622)	0.000 (-0.034, 0.034)
Post-menopausal breast	0.594 (0.579, 0.609)	0.603 (0.588, 0.617)	0.009 (-0.005, 0.023)	0.603 (0.588, 0.617)	0.010 (-0.005, 0.025)	0.001 (-0.004, 0.006)	0.594 (0.579, 0.609)	0.600 (0.586, 0.615)	0.006 (-0.004, 0.017)

* Multivariable adjustment for baseline age, ethnicity, alcohol, smoking and HRT (in women).

Abbreviations: SE, standard error; OBR, obesity-related; NOBR, non-obesity related; BMI, body mass index; AIC, Akaike information criterion.

Key: Green – significant difference in C-statistic.

Analysis using predicted BMI from the at least 1 observed BMI readings subgroup

a) **Analysis of overweight-years exposure**

Table S34: Summary of the exposure metrics.

**Characteristic**	**Men**<br>**Mean (SD)**
**Baseline BMI, (kg/m^2)**	27.70 (4.30)
**End of cancer follow up, (years)**	18.00 (8.00)
**Total cumulative overweight years, ($kg\text{-}years/m^2$)**	49.00 (64.00)
**Total cumulative overweight degree, (kg/m^2)**	53.00 (67.00)
**Total cumulative overweight duration, (years)**	16.00 (12.00)
**Total cumulative obese-years, ($kg\text{-}years/m^2$)**	8.00 (26.00)
**Total cumulative obese-degree, (kg/m^2)**	9.00 (28.00)
**Total cumulative obese-duration, (years)**	3.00 (7.00)
	**Women**<br>**Mean (SD)**
**Baseline BMI, (kg/m^2)**	28.20 (6.20)
**End of cancer follow up, (years)**	20.00 (7.00)
**Total cumulative overweight years, ($kg\text{-}years/m^2$)**	85.00 (100.00)
**Total cumulative overweight degree, (kg/m^2)**	89.00 (104.00)
**Total cumulative overweight duration, (years)**	19.00 (13.00)
**Total cumulative obese-years, ($kg\text{-}years/m^2$)**	24.00 (54.00)
**Total cumulative obese-degree, (kg/m^2)**	26.00 (57.00)
**Total cumulative obese-duration, (years)**	7.00 (10.00)
**Abbreviations:** N, number of participants; SD, standard deviation; BMI, body mass index.	

Table S35: Incidence of cancer (events/1000 Person-Years) according to overweight years at Visit 2 and BMI at Visit 2 stratified by gender, ethnicity, smoking, and HRT (women only) in the ARIC cohort.

	0 overweight-years (kg-years/m^2)			>0-100 overweight-years (kg-years/m^2)			>100 overweight-years (kg-years/m^2)			Baseline BMI (kg /m^2)		
	N	PYFU	IR (95% CI)	N	PYFU	IR (95% CI)	N	PYFU	IR (95% CI)	N	PYFU	IR (95% CI)
**Men**												
**Whole sample**	461	53953.51	8.54 (7.75, 9.33)	1339	173390.70	7.72 (7.31, 8.14)	384	42263.20	9.09 (8.16, 10.01)	2184	269607.40	8.10 (7.76, 8.44)
**Ethnicity**												
**White**	345	42992.23	8.02 (7.17, 8.88)	1050	140426.00	7.48 (7.02, 7.93)	288	33465.45	8.61 (7.60, 9.62)	1683	216883.70	7.76 (7.39, 8.13)
**Black**	116	10961.28	10.58 (8.61, 12.56)	289	32964.68	8.77 (7.74, 9.79)	96	8797.75	10.91 (8.67, 13.16)	501	52723.71	9.50 (8.66, 10.34)
**Smoking**												
**Ever**	368	39933.97	9.22 (8.26, 10.17)	1004	125013.20	8.03 (7.53, 8.53)	280	30153.68	9.29 (8.18, 10.39)	1652	195100.80	8.47 (8.05, 8.88)
**Never**	93	14019.54	6.63 (5.25, 8.02)	335	48377.52	6.93 (6.18, 7.69)	104	12109.51	8.59 (6.89, 10.28)	533	74506.58	7.15 (6.53, 7.76)
**Women**												
**Whole sample**	359	77046.69	4.66 (4.17, 5.15)	829	170472.80	4.86 (4.53, 5.20)	636	117300.40	5.42 (5.00, 5.85)	1824	364819.90	5.00 (4.77, 5.23)
**Ethnicity**												
**White**	315	68000.61	4.63 (4.11, 5.15)	622	122840.30	5.06 (4.66, 5.47)	407	74008.82	5.50 (4.96, 6.04)	1344	264849.70	5.07 (4.80, 5.35)
**Black**	44	9046.08	4.86 (3.37, 6.36)	207	47632.52	4.35 (3.74, 4.95)	229	43291.60	5.29 (4.59, 5.99)	480	99970.20	4.80 (4.37, 5.24)
**Smoking**												
**Ever**	228	42103.31	5.42 (4.70, 6.13)	460	83641.25	5.50 (4.99, 6.01)	309	51149.80	6.04 (5.35, 6.72)	997	176894.40	5.63 (5.28, 5.99)
**Never**	131	34943.38	3.75 (3.09, 4.41)	369	86831.55	4.25 (3.81, 4.69)	327	66150.62	4.95 (4.40, 5.49)	827	187925.60	4.40 (4.10, 4.70)
**HRT**												
**Ever**	158	34324.50	4.60 (3.87, 5.33)	329	64660.89	5.09 (4.54, 5.65)	148	32284.68	4.59 (3.83, 5.35)	635	131270.10	4.84 (4.46, 5.22)
**Never**	201	42722.19	4.71 (4.05, 5.37)	500	105811.90	4.72 (4.30, 5.14)	488	85015.73	5.74 (5.22, 6.25)	1189	233549.80	5.09 (4.80, 5.38)
**Abbreviations**: N, number of events; PYFR, person-years of follow-up; IR, incidence rate of all cancers; CI, confidence interval.												

Table S36: Hazard ratio of cancers per standard deviation of overweight-years at Visit 2 and BMI at Visit 2, ARIC.

Outcome	Overweight-years (kg-years/m²) (per SD)		BMI (kg /m²) (per SD)	
	Age-adjusted HR (95% CI)	MV-adjusted HR (95% CI)	Age-adjusted HR (95% CI)	MV-adjusted HR (95% CI)
**Men**				
All Cancers	1.03 (0.99,1.07)	1.03 (0.99,1.07)	1.02 (0.98,1.06)	1.02 (0.98,1.07)
OBR-cancers	1.15 (1.06,1.25)	1.15 (1.05,1.25)	1.15 (1.05,1.27)	1.15 (1.05,1.26)
NOBR-cancers	1.00 (0.95,1.05)	1.00 (0.95,1.05)	0.99 (0.94,1.04)	0.99 (0.94,1.04)
NOBR-cancers excluding lung and prostate	1.01 (0.93,1.09)	1.02 (0.94,1.10)	1.05 (0.96,1.14)	1.06 (0.97,1.15)
**Specific cancer sites**				
Colorectal	1.32 (1.18,1.47)	1.31 (1.18,1.46)	1.29 (1.13,1.48)	1.29 (1.12,1.47)
Kidney	0.99 (0.77,1.28)	0.99 (0.77,1.27)	1.02 (0.80,1.31)	1.02 (0.80,1.31)
Bladder	1.16 (0.95,1.42)	1.17 (0.95,1.44)	1.14 (0.91,1.44)	1.15 (0.91,1.45)
Pancreas	0.95 (0.72,1.24)	0.95 (0.72,1.24)	1.11 (0.86,1.42)	1.11 (0.87,1.41)
Lung	0.90 (0.80,1.01)	0.91 (0.81,1.03)	0.77 (0.68,0.88)	0.79 (0.70,0.90)
Prostate	1.03 (0.96,1.10)	1.02 (0.95,1.09)	1.03 (0.96,1.11)	1.02 (0.95,1.10)
Metastatic prostate cancer	1.16 (0.91,1.46)	1.15 (0.91,1.45)	1.06 (0.81,1.38)	1.05 (0.81,1.37)
**Women**				
All Cancers	1.07 (1.02,1.12)	1.09 (1.04,1.14)	1.11 (1.06,1.16)	1.14 (1.08,1.19)
OBR-cancers	1.14 (1.08,1.20)	1.14 (1.08,1.20)	1.17 (1.11,1.24)	1.18 (1.11,1.25)
NOBR-cancers	0.96 (0.89,1.04)	1.00 (0.92,1.08)	0.99 (0.92,1.07)	1.06 (0.97,1.15)
NOBR-cancers excluding lung	1.01 (0.92,1.1)	1.03 (0.94,1.13)	1.07 (0.98,1.17)	1.13 (1.02,1.24)
**Specific cancer sites**				
Colorectal	1.08 (0.94,1.24)	1.05 (0.91,1.21)	1.18 (1.03,1.36)	1.14 (0.98,1.32)
Pancreas	1.39 (1.13,1.70)	1.31 (1.06,1.62)	1.28 (1.00,1.63)	1.15 (0.88,1.50)
Kidney	1.32 (1.07,1.62)	1.27 (1.02,1.57)	1.43 (1.15,1.77)	1.36 (1.08,1.72)
Lung	0.86 (0.75,1.00)	0.93 (0.80,1.07)	0.83 (0.72,0.96)	0.92 (0.79,1.07)
Endometrial	1.44 (1.24,1.66)	1.50 (1.28,1.75)	1.58 (1.36,1.84)	1.73 (1.48,2.03)
Ovarian	1.06 (0.83,1.36)	1.12 (0.87,1.44)	1.01 (0.79,1.30)	1.10 (0.85,1.43)
Post-menopausal breast cancer	1.06 (0.98,1.15)	1.07 (0.99,1.17)	1.08 (1.00,1.18)	1.11 (1.02,1.21)

* Multivariable adjustment for baseline age, ethnicity, alcohol, smoking and HRT (in women).
Abbreviations: OBR, obesity-related; NOBR, non-obesity related; CI, confidence interval; HR, hazard ratio; BMI, body mass index; MV, multivariable; SD, standard deviation.

Table S37: Hazard ratio of specific cancers per 100 overweight-years and per 5-unit (kg/m^2) BMI in the ARIC cohort.

Outcomes	Overweight-years (per 100 (kg-years/m^2))		BMI (per 5 unit [kg/m^2])	
	Age-adjusted HR (95% CI)	MV-adjusted HR (95% CI)	Age-adjusted HR (95% CI)	MV-adjusted HR (95% CI)
**Men**				
All Cancers	1.05 (0.98,1.12)	1.05 (0.98,1.12)	1.02 (0.97,1.08)	1.02 (0.97,1.08)
OBR-cancers	1.24 (1.09,1.42)	1.24 (1.09,1.42)	1.18 (1.06,1.32)	1.18 (1.06,1.32)
NOBR-cancers	1.00 (0.92,1.08)	1.00 (0.93,1.08)	0.98 (0.93,1.04)	0.99 (0.93,1.05)
NOBR-cancers excluding lung and prostate	1.01 (0.89,1.15)	1.03 (0.90,1.17)	1.06 (0.96,1.16)	1.07 (0.97,1.17)
**Specific cancer sites**				
Colorectal	1.55 (1.31,1.83)	1.54 (1.30,1.82)	1.35 (1.16,1.58)	1.34 (1.15,1.57)
Kidney	0.99 (0.66,1.47)	0.98 (0.66,1.47)	1.03 (0.77,1.37)	1.03 (0.77,1.37)
Bladder	1.26 (0.92,1.73)	1.28 (0.93,1.78)	1.17 (0.89,1.53)	1.18 (0.90,1.55)
Pancreas	0.92 (0.60,1.40)	0.92 (0.60,1.40)	1.13 (0.84,1.51)	1.13 (0.84,1.50)
Lung	0.85 (0.70,1.02)	0.86 (0.72,1.04)	0.74 (0.64,0.86)	0.76 (0.66,0.88)
Prostate	1.05 (0.94,1.17)	1.03 (0.93,1.15)	1.04 (0.95,1.13)	1.03 (0.95,1.11)
Metastatic prostate	1.26 (0.87,1.83)	1.24 (0.86,1.81)	1.07 (0.78,1.46)	1.06 (0.78,1.45)
**Women**				
All Cancers	1.07 (1.02,1.12)	1.09 (1.04,1.14)	1.09 (1.05,1.13)	1.11 (1.07,1.15)
OBR-cancers	1.14 (1.08,1.20)	1.14 (1.08,1.20)	1.14 (1.09,1.19)	1.15 (1.09,1.20)
NOBR-cancers	0.96 (0.89,1.04)	1.00 (0.92,1.08)	0.99 (0.93,1.06)	1.05 (0.98,1.12)
NOBR-cancers excluding lung	1.01 (0.92,1.10)	1.03 (0.94,1.14)	1.06 (0.98,1.14)	1.1 (1.02,1.19)
**Specific cancer sites**				
Colorectal	1.08 (0.94,1.24)	1.05 (0.91,1.21)	1.15 (1.02,1.28)	1.11 (0.98,1.25)
Pancreas	1.39 (1.14,1.71)	1.31 (1.06,1.63)	1.22 (1.00,1.49)	1.12 (0.90,1.39)
Kidney	1.32 (1.07,1.62)	1.27 (1.02,1.57)	1.34 (1.12,1.59)	1.28 (1.06,1.55)
Lung	0.86 (0.75,1.00)	0.92 (0.80,1.07)	0.86 (0.76,0.97)	0.93 (0.82,1.05)
Endometrial	1.44 (1.24,1.67)	1.50 (1.29,1.75)	1.45 (1.29,1.64)	1.57 (1.38,1.78)
Ovarian	1.06 (0.83,1.36)	1.12 (0.87,1.44)	1.01 (0.82,1.24)	1.08 (0.87,1.34)
Post-menopausal breast cancer	1.06 (0.98,1.15)	1.07 (0.99,1.17)	1.07 (1.00,1.14)	1.09 (1.01,1.17)

* Multivariable adjustment for baseline age, ethnicity, alcohol, smoking and HRT (in women).
Abbreviations: OBR, obesity-related; NOBR, non-obesity related; CI, confidence interval; HR, hazard ratio; BMI, body mass index; MV, multivariable.

Table S38: Hazard ratios of cancers per standard deviation overweight degree and duration at Visit 2, in ARIC.

Outcome	Degree of Overweight (kg /m^2) (per SD)		Duration of Overweight (years) (per SD)	
	Age-adjusted HR (95% CI)	MV-adjusted HR (95% CI)	Age-adjusted HR (95% CI)	MV-adjusted HR (95% CI)
**Men**				
All Cancers	1.04 (0.98,1.10)	1.03 (0.97,1.10)	0.97 (0.92,1.03)	0.98 (0.93,1.04)
OBR-cancers	1.10 (0.97,1.25)	1.10 (0.97,1.25)	0.96 (0.85,1.08)	0.96 (0.85,1.09)
NOBR-cancers	1.02 (0.95,1.09)	1.01 (0.95,1.09)	0.98 (0.92,1.04)	0.99 (0.93,1.05)
NOBR-cancers excluding lung and prostate	0.95 (0.85,1.08)	0.96 (0.85,1.09)	0.95 (0.85,1.05)	0.95 (0.86,1.05)
**Specific cancer sites**				
Colorectal	1.29 (1.10,1.50)	1.29 (1.10,1.50)	1.09 (0.91,1.31)	1.10 (0.92,1.31)
Kidney	0.95 (0.65,1.38)	0.94 (0.65,1.37)	0.81 (0.58,1.13)	0.80 (0.57,1.12)
Bladder	1.13 (0.85,1.51)	1.15 (0.86,1.54)	1.05 (0.78,1.40)	1.05 (0.79,1.40)
Pancreas	0.75 (0.48,1.17)	0.75 (0.48,1.17)	0.91 (0.66,1.25)	0.92 (0.67,1.27)
Lung	1.10 (0.95,1.27)	1.11 (0.95,1.28)	0.95 (0.83,1.09)	0.97 (0.84,1.11)
Prostate	1.02 (0.93,1.13)	1.01 (0.92,1.12)	1.02 (0.94,1.11)	1.04 (0.95,1.13)
Metastatic prostate cancer	1.23 (0.89,1.70)	1.22 (0.88,1.68)	1.21 (0.87,1.68)	1.23 (0.88,1.71)
**Women**				
All Cancers	0.99 (0.93,1.06)	0.99 (0.93,1.06)	0.98 (0.93,1.04)	0.99 (0.93,1.05)
OBR-cancers	1.03 (0.95,1.12)	1.03 (0.95,1.12)	1.04 (0.96,1.12)	1.04 (0.96,1.12)
NOBR-cancers	0.93 (0.83,1.04)	0.93 (0.82,1.04)	0.92 (0.84,1.01)	0.93 (0.84,1.02)
NOBR-cancers excluding lung	0.91 (0.79,1.05)	0.91 (0.79,1.04)	0.94 (0.84,1.05)	0.94 (0.84,1.06)
**Specific cancer sites**				
Colorectal	0.90 (0.72,1.12)	0.91 (0.73,1.14)	1.03 (0.86,1.25)	1.03 (0.85,1.25)
Pancreas	1.45 (1.07,1.96)	1.46 (1.08,1.98)	1.26 (0.89,1.77)	1.25 (0.89,1.77)
Kidney	1.05 (0.74,1.49)	1.07 (0.76,1.51)	1.08 (0.77,1.52)	1.08 (0.77,1.52)
Lung	0.97 (0.79,1.18)	0.96 (0.78,1.18)	0.89 (0.76,1.05)	0.90 (0.77,1.06)
Endometrial	1.03 (0.80,1.34)	1.02 (0.78,1.33)	1.05 (0.80,1.37)	1.04 (0.80,1.36)
Ovarian	1.13 (0.79,1.61)	1.11 (0.77,1.59)	1.14 (0.83,1.58)	1.15 (0.83,1.58)
Post-menopausal breast cancer	1.01 (0.89,1.14)	1.00 (0.89,1.14)	1.03 (0.92,1.14)	1.03 (0.92,1.14)

* Multivariable adjustment for baseline age, ethnicity, alcohol, smoking and HRT (in women).
* Degree of overweight is the cumulative sum of the number of BMI units ≥ 25 kg/m^2 over the exposure period.
* Duration of overweight is the cumulative sum of the duration overweight (BMI ≥ 25 kg/m^2) over the exposure period.
Abbreviations: OBR, obesity-related; NOBR, non-obesity related; CI, confidence interval; HR, hazard ratio; BMI, body mass index; MV, multivariable.

Table S39: Hazard ratio of cancers by overweight degree and duration per 10 units and per 10 years, respectively at Visit 2, ARIC.

Outcome	Degree of Overweight (per 10 (kg /m^2))		Duration of Overweight (per 10 years)	
	Age-adjusted HR (95% CI)	MV-adjusted HR (95% CI)	Age-adjusted HR (95% CI)	MV-adjusted HR (95% CI)
**Men**				
All Cancers	1.01 (1.00,1.01)	1.01 (1.00,1.01)	0.98 (0.93,1.02)	0.98 (0.94,1.03)
OBR-cancers	1.01 (1.00,1.03)	1.01 (1.00,1.03)	0.96 (0.87,1.07)	0.97 (0.87,1.07)
NOBR-cancers	1.00 (0.99,1.01)	1.00 (0.99,1.01)	0.98 (0.93,1.03)	0.99 (0.94,1.04)
NOBR-cancers excluding lung and prostate	0.99 (0.97,1.01)	0.99 (0.98,1.01)	0.95 (0.87,1.04)	0.96 (0.88,1.04)
**Specific cancer sites**				
Colorectal	1.04 (1.02,1.06)	1.04 (1.01,1.06)	1.08 (0.93,1.25)	1.08 (0.93,1.26)
Kidney	0.99 (0.94,1.05)	0.99 (0.94,1.05)	0.84 (0.63,1.11)	0.83 (0.62,1.1)
Bladder	1.02 (0.98,1.06)	1.02 (0.98,1.07)	1.04 (0.81,1.33)	1.04 (0.82,1.33)
Pancreas	0.96 (0.89,1.02)	0.96 (0.89,1.02)	0.92 (0.70,1.21)	0.93 (0.71,1.23)
Lung	1.01 (0.99,1.04)	1.02 (0.99,1.04)	0.96 (0.85,1.07)	0.97 (0.87,1.09)
Prostate	1.00 (0.99,1.02)	1.00 (0.99,1.02)	1.02 (0.95,1.1)	1.03 (0.96,1.11)
Metastatic prostate cancer	1.03 (0.98,1.08)	1.03 (0.98,1.08)	1.17 (0.88,1.56)	1.19 (0.90,1.58)
**Women**				
All Cancers	1.00 (0.99,1.01)	1.00 (0.99,1.01)	0.99 (0.94,1.03)	0.99 (0.95,1.04)
OBR-cancers	1.00 (0.99,1.01)	1.00 (0.99,1.01)	1.03 (0.97,1.09)	1.03 (0.97,1.09)
NOBR-cancers	0.99 (0.98, 1.00)	0.99 (0.98, 1.00)	0.94 (0.87,1.01)	0.94 (0.88,1.01)
NOBR-cancers excluding lung	0.99 (0.98, 1.00)	0.99 (0.98, 1.00)	0.95 (0.87,1.04)	0.96 (0.88,1.05)
**Specific cancer sites**				
Colorectal	0.99 (0.97,1.01)	0.99 (0.97,1.01)	1.03 (0.89,1.19)	1.02 (0.89,1.18)
Pancreas	1.04 (1.01,1.07)	1.04 (1.01,1.07)	1.19 (0.92,1.55)	1.19 (0.91,1.55)
Kidney	1.00 (0.97,1.04)	1.01 (0.97,1.04)	1.06 (0.82,1.38)	1.06 (0.82,1.38)
Lung	1.00 (0.98,1.02)	1.00 (0.98,1.02)	0.92 (0.81,1.04)	0.92 (0.82,1.05)
Endometrial	1.00 (0.98,1.03)	1.00 (0.98,1.03)	1.03 (0.84,1.27)	1.03 (0.84,1.27)
Ovarian	1.01 (0.98,1.05)	1.01 (0.98,1.05)	1.11 (0.87,1.42)	1.11 (0.87,1.42)
Post-menopausal breast cancer	1.00 (0.99,1.01)	1.00 (0.99,1.01)	1.02 (0.94,1.11)	1.02 (0.94,1.11)

* Multivariable adjustment for baseline age, ethnicity, alcohol, smoking and HRT (in women).
* Degree of overweight is the cumulative sum of the number of BMI units ≥ 25 kg/m^2 over the exposure period.
* Duration of overweight is the cumulative sum of the duration overweight (BMI ≥ 25 kg/m^2) over the exposure period.

Abbreviations: OBR, obesity-related; NOBR, non-obesity related; CI, confidence interval; HR, hazard ratio; BMI, body mass index; MV, multivariable.

Table S40: Comparison of associations of overweight-years at Visit 2 and BMI at Visit 2 with cancer by Akaike information criterion in the ARIC cohort.

	AIC				
**Outcomes**	**MV-adjusted overweight-years**	**MV-adjusted BMI**	**MV-adjusted overweight - years with BMI**	**MV-adjusted degree**	**MV-adjusted duration**
**Men**					
All cancers	33863.89	33864.99	33865.88	33863.78	33865.84
OBR-cancers	6658.39	6659.22	6659.15	6657.94	6666.22
NOBR-cancers	27205.35	27205.19	27207.03	27205.35	27205.04
NOBR-cancers excluding lung and prostate	9475.81	9474.38	9475.92	9475.75	9475.96
**Specific cancer sites**					
Colorectal	2838.93	2847.17	2840.56	2838.67	2851.74
Kidney	1106.83	1106.80	1108.70	1106.83	1105.98
Bladder	1140.79	1141.51	1142.72	1140.76	1141.98
Pancreas	1000.17	999.71	999.73	1000.22	1000.33
Lung	5008.44	4996.50	4996.96	5008.18	5003.27
Prostate	12591.20	12591.20	12593.14	12591.21	12590.58
Metastatic prostate	850.60	851.64	852.34	850.67	850.38
**Women**					
All cancers	29456.34	29443.46	29443.56	29457.03	29463.43
OBR-cancers	18305.03	18296.54	18296.13	18306.02	18311.53
NOBR-cancers	11087.64	11087.56	11085.96	11089.28	11088.92
NOBR-cancers excluding lung	7457.97	7454.51	7452.63	7459.63	7459.97
Colorectal	2917.33	2916.06	2916.59	2918.32	2917.4
Pancreas	878.70	884.80	879.93	880.43	883.19
Kidney	967.97	968.01	968.03	969.67	971.22
Lung	3567.10	3568.72	3568.82	3568.87	3567.18
Endometrial	1790.15	1773.32	1774.14	1789.06	1799.96
Ovarian	1057.35	1059.00	1059.32	1058.70	1058.32
Post-menopausal breast cancer	8940.79	8940.20	8940.54	8942.45	8942.57

* Multivariable adjustment for baseline age, ethnicity, alcohol, smoking and HRT (in women).
Abbreviations: SE, standard error; OBR, obesity-related; NOBR, non-obesity related; BMI, body mass index; AIC, Akaike information criterion; MV, multivariable.

Table S41: Comparison of the overweight-years metric at Visit 2 and BMI at Visit 2 using Harrell's C-statistic, ARIC

Characteristic	Harrell's C-statistic (95% CI)								
	MV-adjusted overweight-years	MV-adjusted BMI	Difference in c-statistic between BMI and overweight-years	MV-adjusted overweight-years with BMI	Difference in c-statistic between MV-adjusted overweight-years with BMI combined compared with overweight-years	Difference in c-statistic between MV-adjusted overweight-years with BMI combined and MV-adjusted BMI	MV-adjusted cumulative overweight degree	MV-adjusted cumulative overweight duration	Difference in c-statistic between MV-adjusted duration and MV-adjusted degree
**Men**									
All cancers	0.600 (0.592, 0.609)	0.600 (0.591, 0.609)	-0.001 (-0.006, 0.004)	0.605 (0.575, 0.637)	0.000 (-0.003, 0.004)	0.001 (-0.005, 0.006)	0.600 (0.591, 0.609)	0.599 (0.590, 0.609)	-0.001 (-0.005, 0.004)
OBR-cancers	0.591 (0.574, 0.608)	0.590 (0.573, 0.607)	-0.001 (-0.011, 0.010)	0.588 (0.572, 0.606)	-0.000 (-0.008, 0.008)	0.001 (-0.007, 0.009)	0.590 (0.574, 0.608)	0.584 (0.567, 0.601)	-0.006 (-0.016, 0.004)
NOBR-cancers	0.606 (0.594, 0.617)	0.607 (0.595, 0.619)	0.001 (-0.005, 0.007)	0.605 (0.592, 0.617)	-0.002 (-0.007, 0.004)	-0.003 (-0.010, 0.004)	0.605 (0.595, 0.616)	0.605 (0.594, 0.617)	-0.000 (-0.009, 0.008)
NOBR-cancers excluding lung and prostate	0.591 (0.577, 0.606)	0.592 (0.577, 0.606)	0.000 (-0.003, 0.004)	0.592 (0.578, 0.607)	0.001 (-0.004, 0.005)	0.000 (-0.002, 0.003)	0.591 (0.577, 0.606)	0.591 (0.577, 0.606)	0.000 (-0.002, 0.002)
**Specific cancer sites**									
Colorectal	0.654 (0.628, 0.681)	0.640 (0.564, 0.727)	-0.014 (-0.032, 0.004)	0.650 (0.624, 0.677)	-0.000 (-0.010, 0.009)	0.014 (-0.002, 0.029)	0.652 (0.626, 0.680)	0.640 (0.614, 0.667)	-0.012 (-0.033, 0.009)
Kidney	0.594 (0.547, 0.646)	0.578 (0.530, 0.630)	-0.016 (-0.059, 0.027)	0.600 (0.552, 0.652)	0.006 (-0.035, 0.047)	0.022 (0.004, 0.040)	0.592 (0.545, 0.643)	0.614 (0.568, 0.663)	0.021 (-0.014, 0.057)
Bladder	0.688 (0.647, 0.730)	0.687 (0.647, 0.730)	-0.000 (-0.020, 0.020)	0.689 (0.649, 0.732)	0.001 (-0.002, 0.006)	0.002 (-0.016, 0.019)	0.687 (0.647, 0.7306)	0.682 (0.643, 0.723)	-0.006 (-0.019, 0.007)
Pancreas	0.542 (0.500, 0.587)	0.544 (0.501, 0.590)	0.002 (-0.0270, 0.030)	0.576 (0.531, 0.624)	0.034 (-0.003, 0.070)	0.032 (0.009, 0.055)	0.541 (0.499, 0.586)	0.536 (0.494, 0.581)	-0.0052 (-0.015, 0.004)
Lung	0.723 (0.705, 0.741)	0.732 (0.714, 0.750)	0.009 (-0.000, 0.019)	0.733 (0.715, 0.751)	0.010 (-0.001, 0.022)	0.001 (-0.002, 0.005)	0.723 (0.705, 0.741)	0.728 (0.711, 0.746)	0.005 (0.000, 0.011)
Prostate	0.602 (0.589, 0.615)	0.600 (0.587, 0.613)	-0.002 (-0.007, 0.004)	0.602 (0.589, 0.616)	0.000 (-0.005, 0.005)	0.005 (-0.004, 0.007)	0.603 (0.589, 0.616)	0.599 (0.586, 0.613)	-0.003 (-0.009, 0.003)
Metastatic prostate	0.600 (0.549, 0.656)	0.594 (0.547, 0.646)	-0.006 (-0.044, 0.033)	0.603 (0.550, 0.661)	0.003 (-0.015, 0.021)	0.008 (-0.037, 0.053)	0.600 (0.549, 0.656)	0.594 (0.541, 0.651)	-0.007 (-0.035, 0.022)
**Women**									
All cancers	0.581 (0.569, 0.593)	0.584 (0.572, 0.596)	0.003 (-0.006, 0.013)	0.585 (0.549, 0.624)	0.005 (-0.010, 0.021)	0.002 (-0.009, 0.013)	0.583 (0.571, 0.591)	0.581 (0.569, 0.594)	-0.002 (-0.012, 0.008)
OBR-cancers	0.561 (0.550, 0.572)	0.570 (0.559, 0.581)	0.009 (-0.008, 0.025)	0.567 (0.557, 0.578)	0.012 (0.001, 0.022)	0.003 (-0.008, 0.013)	0.563 (0.552, 0.573)	0.562 (0.552, 0.572)	-0.001 (-0.015, 0.014)
NOBR-cancers	0.636 (0.622, 0.650)	0.637 (0.623, 0.651)	0.001 (-0.007, 0.009)	0.640 (0.627, 0.655)	0.003 (-0.006, 0.012)	0.002 (-0.004, 0.009)	0.637 (0.623, 0.651)	0.636 (0.623, 0.651)	-0.000 (-0.012, 0.011)
NOBR-cancers excluding lung	0.594 (0.578, 0.611)	0.601 (0.585, 0.617)	0.007 (-0.004, 0.018)	0.605 (0.588, 0.622)	0.009 (-0.006, 0.024)	0.002 (-0.007, 0.011)	0.594 (0.578, 0.611)	0.596 (0.579, 0.612)	0.001 (-0.004, 0.006)
**Specific cancer sites**									
Colorectal	0.576 (0.550, 0.602)	0.590 (0.562, 0.613)	0.011 (-0.010, 0.032)	0.583 (0.558, 0.609)	0.008 (-0.021, 0.036)	-0.004 (-0.018, 0.011)	0.576 (0.551, 0.602)	0.582 (0.557, 0.608)	0.006 (-0.010, 0.022)
Pancreas	0.654 (0.603, 0.710)	0.638 (0.590, 0.693)	-0.017 (-0.07, 0.014)	0.659 (0.610, 0.712)	0.005 (-0.014, 0.024)	0.022 (-0.018, 0.061)	0.654 (0.602, 0.709)	0.628 (0.581, 0.678)	-0.026 (-0.057, 0.005)
Kidney	0.644 (0.593, 0.700)	0.655 (0.604, 0.710)	0.011 (-0.026, 0.047)	0.654 (0.603, 0.710)	0.010 (-0.019, 0.039)	-0.000 (-0.010, 0.009)	0.645 (0.593, 0.701)	0.655 (0.606, 0.708)	0.010 (-0.034, 0.055)
Lung	0.754 (0.733, 0.775)	0.753 (0.733, 0.774)	-0.000 (-0.004, 0.003)	0.754 (0.734, 0.776)	-0.000 (-0.003, 0.002)	0.000 (-0.002, 0.002)	0.754 (0.733, 0.775)	0.752 (0.732, 0.774)	-0.001 (-0.006, 0.003)
Endometrial	0.646 (0.611, 0.684)	0.668 (0.631, 0.707)	0.022 (-0.014, 0.057)	0.668 (0.631, 0.707)	0.022 (-0.013, 0.056)	0.000 (-0.001, 0.002)	0.648 (0.613, 0.686)	0.623 (0.591, 0.656)	-0.026 (-0.054, 0.003)
Ovarian	0.575 (0.534, 0.620)	0.562 (0.522, 0.605)	-0.013 (-0.051, 0.024)	0.574 (0.533, 0.618)	-0.002 (-0.010, 0.007)	0.012 (-0.019, 0.042)	0.575 (0.534, 0.619)	0.586 (0.546, 0.629)	0.012 (-0.024, 0.047)

Post-menopausal breast cancer	0.580 (0.565, 0.595)	0.590 (0.575, 0.605)	0.010 (-0.006, 0.026)	0.588 (0.572, 0.604)	0.009 (-0.005, 0.023)	-0.002 (-0.009, 0.007)	0.581 (0.566, 0.596)	0.580 (0.566, 0.597)	-0.001 (-0.014, 0.012)

Abbreviations: SE, standard error; OBR, obesity-related; NOBR, non-obesity related; BMI, body mass index; AIC, Akaike information criterion.
Key: Green – significant difference in C-statistic.

b) Analysis of obese-years exposure

Table S42: Incidence of cancer (events/1000 Person-Years) according to obese-years at Visit 2 and BMI at Visit 2 by gender, ethnicity, smoking, HRT (women only) in the ARIC cohort.

	0 obese-years (kg-years/m^2)			>0-100 obese-years (kg-years/m^2)			>100 obese-years (kg-years/m^2)			Baseline BMI (kg /m^2)		
	N	PYFU	IR (95% CI)	N	PYFU	IR (95% CI)	N	PYFU	IR (95% CI)	N	PYFU	IR (95% CI)
Men												
**Whole sample**	1577	197023.00	8.00 (7.61, 8.40)	577	68773.53	8.39 (7.70, 9.08)	30	3810.86	7.87 (4.91, 10.84)	2184	269607.40	8.10 (7.76, 8.44)
**Ethnicity**												
**White**	1239	160840.60	7.70 (7.27, 8.14)	426	53258.19	8.00 (7.23, 8.77)	18	2784.94	6.46 (3.27, 9.66)	1683	216883.70	7.76 (7.39, 8.13)
**Black**	338	36182.44	9.34 (8.33, 10.35)	151	15515.35	9.73 (8.15, 11.32)	12	1025.92	11.7 (4.50, 18.89)	501	52723.71	9.50 (8.66, 10.34)
**Smoking**												
**Ever**	1205	143525.00	8.39 (7.92, 8.87)	430	49197.11	8.74 (7.90, 9.58)	17	2378.72	7.15 (3.51, 10.79)	1652	195100.80	8.47 (8.05, 8.88)
**Never**	372.4	53498.02	6.96 (6.24, 7.68)	147	19576.43	7.51 (6.27, 8.75)	13	1432.14	9.08 (3.73, 14.43)	532	74506.58	7.15 (6.53, 7.76)
Women												
**Whole sample**	1020	211612.00	4.82 (-4.63, 14.27)	634	125127.50	5.07 (4.67, 5.47)	170	28080.38	6.05 (5.13, 6.98)	1824	364819.90	5.00 (4.77, 5.23)
**Ethnicity**												
**White**	315	68000.61	4.63 (4.11, 5.15)	622	122840.30	5.06 (4.66, 5.47)	407	74008.82	5.50 (4.96, 6.04)	1344	264849.70	5.07 (4.80, 5.35)
**Black**	196	42989.17	4.56 (3.91, 5.21)	212	43470.80	4.88 (4.21, 5.55)	72	13510.23	5.33 (4.06, 6.60)	480	99970.20	4.80 (4.37, 5.24)
**Smoking**												
**Ever**	603	108399.30	5.56 (5.11, 6.01)	316	56984.72	5.54 (4.92, 6.16)	78	11510.35	6.78 (5.23, 8.33)	997	176894.40	5.63 (5.28, 5.99)
**Never**	417	103212.70	4.04 (3.65, 4.43)	318	68142.82	4.67 (4.15, 5.19)	92	16570.03	5.55 (4.39, 6.72)	827	187925.60	4.40 (4.10, 4.70)
**HRT**												
**Ever**	428	85945.53	4.97 (4.50, 5.45)	181	39409.48	4.59 (3.91, 5.27)	27	5915.06	4.54 (2.73, 6.36)	635	131270.10	4.84 (4.46, 5.22)
**Never**	593	125666.50	4.72 (4.33, 5.10)	453	85718.05	5.28 (4.79, 5.78)	143	22165.32	6.46 (5.37, 7.54)	1189	233549.80	5.09 (4.80, 5.38)

* Multivariable adjustment for baseline age, ethnicity, alcohol, smoking and HRT (in women).
Abbreviations: N, number of events; PYFR, person-years of follow-up; IR, incidence rate of all cancers; BMI, body mass index.

Table S43: Hazard ratio of cancers by obese-years at Visit 2 and BMI at Visit 2 in ARIC.

Outcome	Obese-years (kg-years/m^2) (per SD)		BMI (kg /m^2) (per SD)	
	Age-adjusted HR (95% CI)	MV-adjusted HR (95% CI)	Age-adjusted HR (95% CI)	MV-adjusted HR (95% CI)
**Men**				
All Cancers	1.04 (1.00,1.09)	1.04 (1.00,1.08)	1.02 (0.98,1.06)	1.02 (0.98,1.07)
OBR-cancers	1.14 (1.07,1.22)	1.14 (1.06,1.22)	1.15 (1.05,1.27)	1.15 (1.05,1.26)
NOBR-cancers	1.01 (0.97,1.06)	1.01 (0.96,1.06)	0.99 (0.94,1.04)	0.99 (0.94,1.04)
NOBR-cancers excluding lung and prostate	1.02 (0.94,1.10)	1.02 (0.94,1.11)	1.05 (0.96,1.14)	1.06 (0.97,1.15)
**Specific cancer sites**				
Colorectal	1.22 (1.13,1.32)	1.22 (1.12,1.32)	1.29 (1.13,1.48)	1.29 (1.12,1.47)
Kidney	1.06 (0.86,1.32)	1.06 (0.85,1.31)	1.02 (0.80,1.31)	1.02 (0.80,1.31)
Bladder	1.08 (0.89,1.31)	1.10 (0.90,1.35)	1.14 (0.91,1.44)	1.15 (0.91,1.45)
Pancreas	0.88 (0.62,1.26)	0.88 (0.61,1.25)	1.11 (0.86,1.42)	1.11 (0.87,1.41)
Lung	0.94 (0.82,1.07)	0.95 (0.83,1.08)	0.77 (0.68,0.88)	0.79 (0.70,0.90)
Prostate	1.03 (0.97,1.10)	1.02 (0.96,1.09)	1.03 (0.96,1.11)	1.02 (0.95,1.10)
Metastatic prostate	1.11 (0.90,1.36)	1.10 (0.89,1.35)	1.06 (0.81,1.38)	1.05 (0.81,1.37)
**Women**				
All Cancers	1.07 (1.02,1.11)	1.08 (1.03,1.13)	1.11 (1.06,1.16)	1.14 (1.08,1.19)
OBR-cancers	1.11 (1.06,1.17)	1.11 (1.05,1.17)	1.17 (1.11,1.24)	1.18 (1.11,1.25)
NOBR-cancers	0.99 (0.91,1.07)	1.02 (0.94,1.10)	0.99 (0.92,1.07)	1.06 (0.97,1.15)
NOBR-cancers excluding lung	1.03 (0.94,1.13)	1.05 (0.96,1.15)	1.07 (0.98,1.17)	1.13 (1.02,1.24)
**Specific cancer sites**				
Colorectal	1.05 (0.91,1.20)	1.02 (0.88,1.17)	1.18 (1.03,1.36)	1.14 (0.98,1.32)
Pancreas	1.27 (1.08,1.51)	1.21 (1.02,1.45)	1.28 (1.00,1.63)	1.15 (0.88,1.50)
Kidney	1.27 (1.08,1.50)	1.23 (1.04,1.46)	1.43 (1.15,1.77)	1.36 (1.08,1.72)
Lung	0.89 (0.76,1.04)	0.94 (0.80,1.10)	0.83 (0.72,0.96)	0.92 (0.79,1.07)
Endometrial	1.31 (1.16,1.47)	1.35 (1.20,1.53)	1.58 (1.36,1.84)	1.73 (1.48,2.03)
Ovarian	1.07 (0.86,1.35)	1.12 (0.89,1.41)	1.01 (0.79,1.30)	1.10 (0.85,1.43)
Post-menopausal breast cancer	1.04 (0.96,1.13)	1.05 (0.97,1.14)	1.08 (1.00,1.18)	1.11 (1.02,1.21)

* Multivariable adjustment for baseline age, ethnicity, alcohol, smoking and HRT (in women).
Abbreviations: OBR, obesity-related; NOBR, non-obesity related; CI, confidence interval; HR, hazard ratio; BMI, body mass index; MV, multivariable; SD, standard deviation.

Table S44: Hazard ratio of cancers by obese-years per 100 units at Visit 2 and BMI per 5 units (kg/m^2) at Visit 2 in ARIC.

Outcomes	Obese-years (per 100 kg-years/m^2)		BMI (per 5 unit [kg/m^2])	
	Age-adjusted HR (95% CI)	MV-adjusted HR (95% CI)	Age-adjusted HR (95% CI)	MV-adjusted HR (95% CI)
**Men**				
All Cancers	1.19 (1.02,1.39)	1.18 (1.01,1.38)	1.02 (0.97,1.08)	1.02 (0.97,1.08)
OBR-cancers	1.69 (1.30,2.21)	1.68 (1.28,2.19)	1.18 (1.06,1.32)	1.18 (1.06,1.32)
NOBR-cancers	1.05 (0.87,1.27)	1.04 (0.86,1.26)	0.98 (0.93,1.04)	0.99 (0.93,1.05)
NOBR-cancers excluding lung and prostate	1.07 (0.77,1.47)	1.1 (0.80,1.51)	1.06 (0.96,1.16)	1.07 (0.97,1.17)
**Specific cancer sites**				
Colorectal	2.20 (1.61,3.01)	2.18 (1.59,3.00)	1.35 (1.16,1.58)	1.34 (1.15,1.57)
Kidney	1.27 (0.54,2.98)	1.25 (0.54,2.91)	1.03 (0.77,1.37)	1.03 (0.77,1.37)
Bladder	1.37 (0.63,2.96)	1.47 (0.66,3.25)	1.17 (0.89,1.53)	1.18 (0.90,1.55)
Pancreas	0.61 (0.15,2.48)	0.59 (0.14,2.44)	1.13 (0.84,1.51)	1.13 (0.84,1.5)
Lung	0.77 (0.46,1.30)	0.80 (0.47,1.38)	0.74 (0.64,0.86)	0.76 (0.66,0.88)
Prostate	1.15 (0.88,1.48)	1.09 (0.84,1.41)	1.04 (0.95,1.13)	1.03 (0.95,1.11)
Metastatic prostate	1.51 (0.67,3.4)	1.44 (0.63,3.27)	1.07 (0.78,1.46)	1.06 (0.78,1.45)

**Women**				
All Cancers	1.13 (1.04,1.22)	1.15 (1.06,1.25)	1.09 (1.05,1.13)	1.11 (1.07,1.15)
OBR-cancers	1.22 (1.11,1.33)	1.21 (1.10,1.33)	1.14 (1.09,1.19)	1.15 (1.09,1.20)
NOBR-cancers	0.98 (0.85,1.13)	1.04 (0.9,1.2)	0.99 (0.93,1.06)	1.05 (0.98,1.12)
NOBR-cancers excluding lung	1.06 (0.90,1.25)	1.10 (0.93,1.3)	1.06 (0.98,1.14)	1.10 (1.02,1.19)
**Specific cancer sites**				
Colorectal	1.09 (0.84,1.41)	1.03 (0.79,1.34)	1.15 (1.02,1.28)	1.11 (0.98,1.25)
Pancreas	1.57 (1.15,2.15)	1.43 (1.03,1.99)	1.22 (1.00,1.49)	1.12 (0.90,1.39)
Kidney	1.56 (1.15,2.12)	1.47 (1.07,2.02)	1.34 (1.12,1.59)	1.28 (1.06,1.55)
Lung	0.80 (0.59,1.07)	0.89 (0.66,1.20)	0.86 (0.76,0.97)	0.93 (0.82,1.05)
Endometrial	1.65 (1.32,2.05)	1.76 (1.40,2.21)	1.45 (1.29,1.64)	1.57 (1.38,1.78)
Ovarian	1.08 (0.93,1.25)	1.09 (0.94,1.27)	1.07 (1.00,1.14)	1.09 (1.01,1.17)
Post-menopausal breast cancer	0.94 (0.46,1.95)	0.97 (0.46,2.03)	0.97 (0.71,1.34)	0.99 (0.71,1.39)

* Multivariable adjustment for baseline age, ethnicity, alcohol, smoking and HRT (in women).
Abbreviations: OBR, obesity-related; NOBR, non-obesity related; CI, confidence interval; HR, hazard ratio; BMI, body mass index; MV, multivariable.

Table S45: Hazard ratio of cancers by obesity degree and duration at Visit 2, ARIC.

Outcome	Degree of Obesity (kg /m^2) (per SD)		Duration of Obesity (years) (per SD)	
	Age-adjusted HR (95% CI)	MV-adjusted HR (95% CI)	Age-adjusted HR (95% CI)	MV-adjusted HR (95% CI)
**Men**				
All Cancers	1.06 (1.01,1.11)	1.05 (1.00,1.10)	1.06 (1.01,1.12)	1.06 (1.01,1.11)
OBR-cancers	1.12 (1.02,1.22)	1.11 (1.02,1.22)	1.11 (1.00,1.23)	1.10 (0.99,1.23)
NOBR-cancers	1.03 (0.97,1.09)	1.03 (0.97,1.09)	1.05 (0.99,1.11)	1.05 (0.99,1.11)
NOBR-cancers excluding lung and prostate	0.99 (0.89,1.10)	0.99 (0.90,1.10)	1.01 (0.91,1.11)	1.02 (0.92,1.12)
**Specific cancer sites**				
Colorectal	1.17 (1.04,1.31)	1.17 (1.04,1.31)	1.21 (1.05,1.39)	1.21 (1.05,1.38)
Kidney	1.07 (0.83,1.40)	1.07 (0.82,1.39)	1.11 (0.83,1.47)	1.10 (0.83,1.47)
Bladder	1.00 (0.76,1.32)	1.02 (0.78,1.35)	1.13 (0.88,1.45)	1.14 (0.89,1.46)
Pancreas	0.74 (0.46,1.19)	0.73 (0.45,1.18)	0.86 (0.60,1.24)	0.85 (0.59,1.23)
Lung	1.09 (0.96,1.24)	1.10 (0.96,1.27)	1.16 (1.03,1.30)	1.15 (1.02,1.30)
Prostate	1.03 (0.95,1.12)	1.01 (0.93,1.10)	1.03 (0.95,1.12)	1.02 (0.94,1.11)
Metastatic prostate	1.12 (0.86,1.45)	1.10 (0.84,1.43)	1.14 (0.85,1.54)	1.13 (0.83,1.52)
**Women**				
All Cancers	1.00 (0.95,1.07)	1.00 (0.94,1.06)	1.01 (0.95,1.07)	1.00 (0.95,1.07)
OBR-cancers	1.01 (0.94,1.08)	1.01 (0.93,1.08)	1.01 (0.94,1.09)	1.01 (0.94,1.10)
NOBR-cancers	0.99 (0.90,1.10)	0.98 (0.88,1.09)	0.99 (0.90,1.09)	0.99 (0.89,1.09)
NOBR-cancers excluding lung	0.98 (0.86,1.11)	0.97 (0.85,1.10)	0.96 (0.85,1.09)	0.96 (0.85,1.08)
**Specific cancer sites**				
Colorectal	0.88 (0.72,1.08)	0.89 (0.73,1.09)	0.89 (0.73,1.09)	0.90 (0.74,1.10)
Pancreas	1.24 (0.96,1.59)	1.25 (0.98,1.61)	1.38 (1.04,1.83)	1.40 (1.06,1.85)
Kidney	1.07 (0.82,1.41)	1.09 (0.83,1.42)	1.04 (0.76,1.41)	1.06 (0.78,1.44)
Lung	1.00 (0.83,1.21)	0.99 (0.81,1.21)	1.06 (0.89,1.25)	1.05 (0.89,1.25)
Endometrial	0.97 (0.79,1.2)	0.97 (0.78,1.2)	0.99 (0.78,1.26)	0.97 (0.76,1.23)
Ovarian	1.13 (0.83,1.52)	1.11 (0.82,1.51)	0.89 (0.62,1.28)	0.87 (0.61,1.25)
Post-menopausal breast cancer	0.98 (0.87,1.09)	0.97 (0.87,1.09)	1.04 (0.93,1.16)	1.04 (0.93,1.15)

* Multivariable adjustment for baseline age, ethnicity, alcohol, smoking and HRT (in women).
* Degree of obese is the cumulative sum of the number of BMI units ≥ 30 kg/m^2 over the exposure period.
* Duration of obese is the cumulative sum of the duration overweight (BMI ≥ 30 kg/m^2) over the exposure period.
Abbreviations: OBR, obesity-related; NOBR, non-obesity related; CI, confidence interval; HR, hazard ratio; BMI, body mass index; MV, multivariable.

Table S46: Hazard ratio of cancers by obesity degree and duration per 10 units and per 10 years, respectively at Visit 2, ARIC.

Outcome	Degree of Obesity (per 10 (kg/m^2))		Duration of Obesity (per 10 years)	
	Age-adjusted HR (95% CI)	MV-adjusted HR (95% CI)	Age-adjusted HR (95% CI)	MV-adjusted HR (95% CI)
**Men**				
All Cancers	1.02 (1.00,1.04)	1.02 (1.00,1.04)	1.10 (1.02,1.18)	1.09 (1.01,1.18)
OBR-cancers	1.04 (1.01,1.08)	1.04 (1.01,1.08)	1.16 (0.99,1.36)	1.16 (0.99,1.36)
NOBR-cancers	1.01 (0.99,1.03)	1.01 (0.99,1.03)	1.08 (0.99,1.17)	1.07 (0.98,1.17)
NOBR-cancers excluding lung and prostate	1.00 (0.96,1.03)	1.00 (0.96,1.04)	1.01 (0.87,1.18)	1.02 (0.88,1.19)
**Specific cancer sites**				
Colorectal	1.06 (1.02,1.11)	1.06 (1.01,1.10)	1.33 (1.08,1.63)	1.32 (1.08,1.63)
Kidney	1.03 (0.93,1.13)	1.03 (0.93,1.13)	1.17 (0.76,1.79)	1.16 (0.76,1.77)
Bladder	1.00 (0.91,1.11)	1.01 (0.91,1.12)	1.20 (0.83,1.74)	1.21 (0.84,1.75)
Pancreas	0.89 (0.75,1.07)	0.89 (0.74,1.06)	0.80 (0.46,1.37)	0.79 (0.46,1.37)
Lung	1.03 (0.99,1.08)	1.04 (0.99,1.09)	1.24 (1.04,1.48)	1.24 (1.03,1.48)
Prostate	1.01 (0.98,1.04)	1.01 (0.97,1.04)	1.05 (0.92,1.19)	1.03 (0.91,1.17)
Metastatic prostate	1.04 (0.94,1.15)	1.04 (0.94,1.14)	1.22 (0.78,1.91)	1.19 (0.76,1.87)
**Women**				
All Cancers	1.00 (0.99,1.01)	1.00 (0.99,1.01)	1.01 (0.95,1.07)	1.00 (0.95,1.07)
OBR-cancers	1.00 (0.99,1.01)	1.00 (0.99,1.01)	1.01 (0.94,1.10)	1.01 (0.94,1.10)
NOBR-cancers	1.00 (0.98,1.02)	1.00 (0.98,1.02)	0.99 (0.90,1.09)	0.99 (0.89,1.09)
NOBR-cancers excluding lung	1.00 (0.97,1.02)	0.99 (0.97,1.02)	0.96 (0.85,1.09)	0.96 (0.85,1.08)
**Specific cancer sites**				
Colorectal	0.98 (0.94,1.01)	0.98 (0.94,1.02)	0.89 (0.73,1.09)	0.90 (0.74,1.10)
Pancreas	1.04 (0.99,1.09)	1.04 (1.00,1.09)	1.38 (1.04,1.83)	1.40 (1.06,1.86)
Kidney	1.01 (0.96,1.06)	1.01 (0.97,1.06)	1.04 (0.76,1.41)	1.06 (0.78,1.44)
Lung	1.00 (0.97,1.03)	1.00 (0.96,1.03)	1.06 (0.89,1.25)	1.05 (0.89,1.25)
Endometrial	1.00 (0.96,1.03)	0.99 (0.96,1.03)	0.99 (0.78,1.27)	0.97 (0.76,1.23)
Ovarian	1.02 (0.97,1.08)	1.02 (0.96,1.08)	0.89 (0.62,1.28)	0.87 (0.61,1.25)
Post-menopausal breast cancer	1.00 (0.98,1.02)	1.00 (0.98,1.02)	1.04 (0.93,1.16)	1.04 (0.93,1.15)

* Multivariable adjustment for baseline age, ethnicity, alcohol, smoking and HRT (in women).
* Degree of obese is the cumulative sum of the number of BMI units ≥ 30 kg/m^2 over the exposure period.
* Duration of obese is the cumulative sum of the duration overweight (BMI ≥ 30 kg/m^2) over the exposure period.
Abbreviations: OBR, obesity-related; NOBR, non-obesity related; CI, confidence interval; HR, hazard ratio; BMI, body mass index; MV, multivariable.

Table S47: Comparison of associations of obese-years at Visit 2 and BMI at Visit 2 with cancer by Akaike information criterion in the ARIC cohort.

	AIC				
**Characteristic**	**MV-adjusted obese-years**	**MV-adjusted BMI**	**MV-adjusted obese -years with BMI**	**MV-adjusted degree**	**MV-adjusted duration**
**Men**					
All cancers	33861.75	33864.99	33863.70	33861.36	33860.46
OBR-cancers	6656.42	6659.22	6656.89	6655.56	6657.35
NOBR-cancers	27205.16	27205.19	27206.56	27205.12	27204.44
NOBR-cancers excluding lung and prostate	9475.65	9474.38	9476.35	9475.57	9474.89
**Specific cancer sites**					
Colorectal	2843.94	2847.17	2843.65	2843.44	2842.33
Kidney	1106.60	1106.80	1108.59	1106.58	1106.44
Bladder	1142.11	1141.51	1143.44	1142.20	1140.63
Pancreas	999.70	999.71	999.19	999.88	1000.31
Lung	5010.19	4996.50	4997.08	5010.16	5010.74
Prostate	12591.19	12591.20	12593.09	12591.18	12591.06
Metastatic prostate	851.15	851.64	853.14	851.23	851.13
**Women**					
All cancers	29459.07	29443.46	29445.46	29458.28	29458.13
OBR-cancers	18312.41	18296.54	18298.50	18311.68	18311.31
NOBR-cancers	11089.01	11087.56	11089.40	11088.95	11088.86
NOBR-cancers excluding lung	7459.02	7454.51	7456.22	7458.86	7459.24
**Specific cancer sites**					
Colorectal	2918.69	2916.06	2916.76	2918.66	2918.65
Pancreas	882.09	884.80	883.98	882.21	880.19
Kidney	969.59	968.01	969.66	969.44	970.54
Lung	3569.38	3568.72	3570.70	3569.39	3569.93
Endometrial	1795.90	1773.32	1775.20	1794.44	1796.96
Ovarian	1058.63	1059.00	1060.60	1058.60	1059.48
Post-menopausal breast cancer	8944.00	8940.20	8942.04	8943.97	8941.49

* Multivariable adjustment for baseline age, ethnicity, alcohol, smoking and HRT (in women).
Abbreviations: SE, standard error; OBR, obesity-related; NOBR, non-obesity related; BMI, body mass index; AIC, Akaike information criterion; MV, multivariable.

Table S48: Comparison of the obese-years metric at Visit 2 and BMI at Visit 2 using Harrell's C-statistic, ARIC

	Harrell's C-statistic (95% CI)								
Characteristic	MV-adjusted obese-years	MV-adjusted BMI	Difference in c-statistic between BMI and obese-years	MV-adjusted obese - years with BMI	Difference in c-statistic between MV-adjusted obese-years with BMI combined compared with obese-years	Difference in c-statistic between MV-adjusted obese-years with BMI combined and MV-adjusted BMI	MV-adjusted cumulative obese degree	MV-adjusted cumulative obese duration	Difference in c-statistic between MV-adjusted duration and MV-adjusted degree
**Men**									
All cancers	0.604 (0.593, 0.616)	0.599 (0.588, 0.610)	-0.006 (-0.019, 0.008)	0.602 (0.591, 0.613)	-0.002 (-0.010, 0.006)	0.003 (-0.008, 0.014)	0.605 (0.594, 0.616)	0.605 (0.593, 0.616)	-0.000 (-0.011, 0.011)
OBR-cancers	0.593 (0.574, 0.612)	0.591 (0.574, 0.608)	-0.003 (-0.016, 0.011)	0.632 (0.614, 0.651)	0.003 (-0.006, 0.012)	0.005 (-0.003, 0.014)	0.595 (0.577, 0.613)	0.597 (0.579, 0.615)	0.002 (-0.006, 0.010)
NOBR-cancers	0.607 (0.597, 0.618)	0.605 (0.594, 0.617)	-0.002 (-0.013, 0.009)	0.608 (0.546, 0.676)	0.000 (-0.009, 0.009)	0.002 (-0.007, 0.010)	0.606 (0.595, 0.618)	0.606 (0.594, 0.618)	0.000 (-0.007, 0.007)
NOBR-cancers excluding lung and prostate	0.590 (0.576, 0.605)	0.591 (0.576, 0.605)	0.000 (-0.003, 0.004)	0.590 (0.576, 0.605)	0.000 (-0.004, 0.004)	0.000 (-0.001, 0.001)	0.590 (0.576, 0.605)	0.590 (0.576, 0.605)	0.000 (-0.002, 0.002)
**Specific cancer sites**									
Colorectal	0.656 (0.628, 0.685)	0.642 (0.615, 0.669)	-0.014 (-0.037, 0.008)	0.656 (0.629, 0.684)	-0.002 (-0.014, 0.011)	0.013 (-0.002, 0.027)	0.658 (0.630, 0.688)	0.664 (0.637, 0.693)	0.006 (-0.010, 0.022)
Kidney	0.611 (0.561, 0.666)	0.603 (0.554, 0.656)	-0.008 (-0.024, 0.007)	0.612 (0.562, 0.667)	0.001 (-0.003, 0.005)	0.009 (-0.009, 0.027)	0.612 (0.561, 0.666)	0.604 (0.554, 0.658)	-0.008 (-0.017, 0.001)
Bladder	0.679 (0.638, 0.724)	0.678 (0.636, 0.723)	-0.001 (-0.028, 0.026)	0.680 (0.638, 0.725)	0.000 (-0.024, 0.024)	0.002 (-0.003, 0.006)	0.679 (0.638, 0.723)	0.677 (0.635, 0.721)	-0.003 (-0.013, 0.008)
Pancreas	0.547 (0.503, 0.594)	0.551 (0.506, 0.599)	0.004 (-0.029, 0.037)	0.583 (0.536, 0.634)	0.036 (-0.007, 0.080)	0.032 (0.012, 0.053)	0.545 (0.502, 0.597)	0.538 (0.495, 0.585)	-0.007 (-0.017, 0.001)
Lung	0.722 (0.704, 0.740)	0.731 (0.713, 0.750)	0.010 (-0.001, 0.020)	0.733 (0.714, 0.752)	0.011 (-0.001 ,0.023)	0.001 (-0.001, 0.004)	0.723 (0.704, 0.740)	0.721 (0.703, 0.739)	-0.001 (-0.002, 0.001)
Prostate	0.605 (0.592, 0.619)	0.606 (0.593, 0.619)	0.001 (-0.004, 0.005)	0.603 (0.590, 0.617)	-0.001 (-0.006, 0.004)	-0.001 (-0.006, 0.003)	0.605 (0.592, 0.619)	0.606 (0.593, 0.620)	0.001 (-0.004, 0.006)
Metastatic prostate	0.595 (0.546, 0.649)	0.587 (0.538, 0.640)	-0.008 (-0.035, 0.018)	0.599 (0.549, 0.653)	0.004 (-0.008, 0.015)	0.012 (-0.021, 0.045)	0.594 (0.544, 0.647)	0.594 (0.544, 0.649)	0.000 (-0.016, 0.016)
**Women**									
All cancers	0.579 (0.567, 0.588)	0.583 (0.573, 0.594)	0.006 (-0.006, 0.017)	0.586 (0.541, 0.634)	0.008 (-0.005, 0.020)	0.002 (-0.006, 0.010)	0.578 (0.567, 0.588)	0.578 (0.568, 0.589)	0.000 (-0.008, 0.009)
OBR-cancers	0.552 (0.542, 0.563)	0.573 (0.563, 0.584)	0.021 (0.007, 0.035)	0.675 (0.658, 0.692)	0.021 (0.005, 0.037)	-0.000 (-0.007, 0.006)	0.552 (0.541, 0.562)	0.555 (0.545, 0.566)	0.003 (-0.007, 0.014)
NOBR-cancers	0.634 (0.620, 0.649)	0.635 (0.621, 0.650)	0.001 (-0.008, 0.010)	0.638 (0.623, 0.653)	0.002 (-0.007, 0.012)	0.001 (-0.003, 0.006)	0.635 (0.621, 0.650)	0.635 (0.621, 0.649)	-0.000 (-0.008, 0.007)
NOBR-cancers excluding lung	0.593 (0.576, 0.610)	0.598 (0.582, 0.615)	0.005 (-0.004, 0.015)	0.595 (0.578, 0.612)	0.007 (-0.004, 0.018)	0.002 (-0.004, 0.008)	0.592 (0.576, 0.609)	0.594 (0.577, 0.610)	0.001 (-0.012, 0.014)
**Specific cancer sites**									
Colorectal	0.568 (0.542, 0.595)	0.586 (0.561, 0.612)	0.018 (-0.008, 0.044)	0.585 (0.559, 0.612)	0.017 (-0.018, 0.052)	-0.001 (-0.020, 0.018)	0.569 (0.543, 0.595)	0.569 (0.544, 0.596)	0.001 (-0.003, 0.005)
Pancreas	0.647 (0.594, 0.705)	0.637 (0.584, 0.694)	-0.011 (-0.032, 0.011)	0.648 (0.595, 0.705)	0.001 (-0.007, 0.008)	0.011 (-0.015, 0.037)	0.648 (0.594, 0.706)	0.654 (0.601, 0.710)	0.006 (-0.018, 0.030)
Kidney	0.652 (0.595, 0.715)	0.663 (0.611, 0.720)	0.011 (-0.033, 0.055)	0.664 (0.611, 0.722)	0.012 (-0.024, 0.047)	0.001 (-0.009, 0.011)	0.653 (0.596, 0.716)	0.641 (0.585, 0.702)	-0.013 (-0.032, 0.006)
Lung	0.754 (0.734, 0.775)	0.753 (0.732, 0.775)	-0.001 (-0.005, 0.003)	0.754 (0.733, 0.776)	-0.001 (-0.005, 0.003)	0.000 (-0.001, 0.002)	0.754 (0.733, 0.775)	0.753 (0.733, 0.774)	-0.000 (-0.003, 0.002)
Endometrial	0.637 (0.600, 0.677)	0.669 (0.631, 0.708)	0.031 (-0.013, 0.075)	0.668 (0.630, 0.707)	0.031 (-0.016, 0.077)	-0.001 (-0.005, 0.003)	0.639 (0.602, 0.679)	0.624 (0.588, 0.662)	-0.015 (-0.030, -0.001)

Ovarian	0.568 (0.526, 0.613)	0.563 (0.523, 0.606)	-0.005 (-0.037, 0.027)	0.569 (0.528, 0.613)	0.001 (-0.009, 0.011)	0.006 (-0.019, 0.031)	0.567 (0.526, 0.612)	0.559 (0.519, 0.602)	-0.008 (-0.042, 0.025)
Post-menopausal breast cancer	0.577 (0.562, 0.592)	0.590 (0.575, 0.605)	0.013 (-0.004, 0.030)	0.589 (0.574, 0.604)	0.0132 (-0.005, 0.032)	0.000 (-0.003, 0.003)	0.577 (0.563, 0.592)	0.583 (0.568, 0.598)	0.006 (-0.006, 0.017)

* Multivariable adjustment for baseline age, ethnicity, alcohol, smoking and HRT (in women).
Abbreviations: SE, standard error; OBR, obesity-related; NOBR, non-obesity related; BMI, body mass index; AIC, Akaike information criterion.
Key: Green – significant difference in C-statistic.

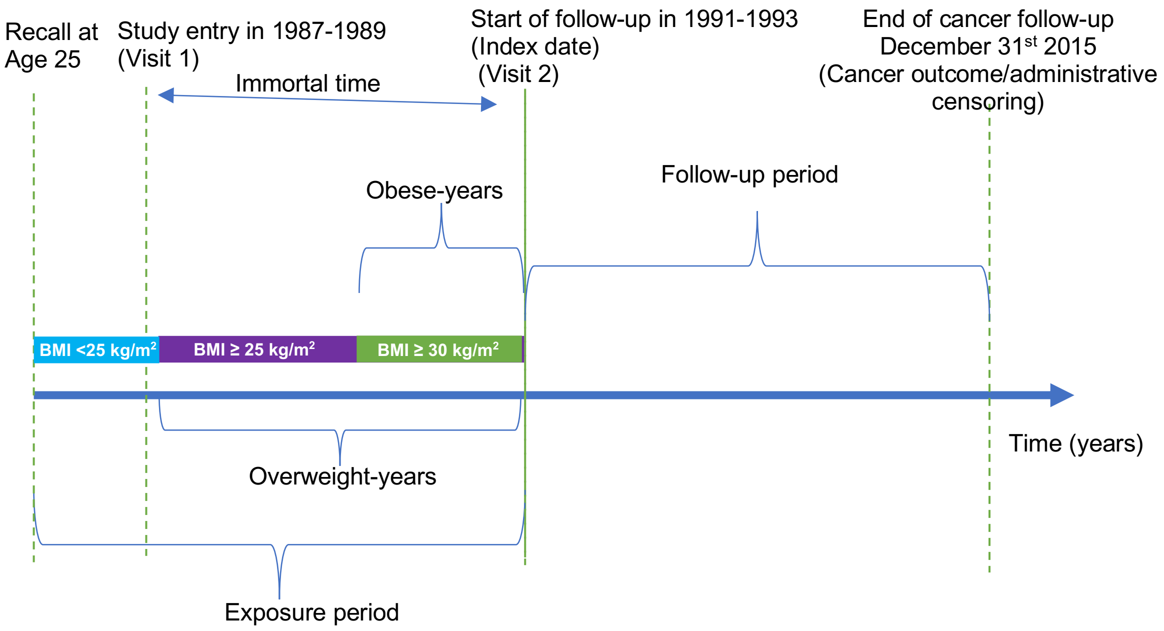

Figure S1: Diagram of the exposure and cancer follow-up period of this study.

The period of exposure was defined from age 25 years old until Visit 2. In the exposure period, overweight-years were calculated for any BMI readings ≥ 25 kg/m^2 including those ≥ 30 kg/m^2. Obese-years were calculated for any BMI readings ≥ 30 kg/m^2 in the exposure period. For both time-fixed overweight years and BMI, the period of follow-up was from Visit 2 until the end of cancer follow-up on Dec 31, 2015. The index date (baseline) was defined at Visit 2.
